# Role of the androgen receptor in melanoma aggressiveness

**DOI:** 10.1038/s41419-025-07350-4

**Published:** 2025-01-21

**Authors:** Marzia Di Donato, Costanza Maria Cristiani, Mariaelena Capone, Cinzia Garofalo, Gabriele Madonna, Lucia Carmela Passacatini, Margaret Ottaviano, Paolo Antonio Ascierto, Ferdinando Auricchio, Ennio Carbone, Antimo Migliaccio, Gabriella Castoria

**Affiliations:** 1 Department of Precision Medicine, University of Campania ‘L. Vanvitelli’- Via L. De Crecchio 7, 80138 Naples, Italy; 2 Neuroscience Research Center, Department of Medical and Surgical Sciences - ‘Magna Graecia’ University of Catanzaro, 88100 Catanzaro, Italy; 3 Department of Melanoma, Cancer Immunotherapy and Development Therapeutics, Istituto Nazionale Tumori IRCCS- Fondazione “G. Pascale”, Napoli, Italy; 4 Department of Experimental and Clinical Medicine, ‘Magna Graecia’ University of Catanzaro, 88100 Catanzaro, Italy; 5 Physiology and Pharmacology of Pain, IRCCS San Raffaele Roma, 00166 Rome, Italy

**Keywords:** Melanoma, Cell invasion, Cancer

## Abstract

Malignant melanoma represents the fifth most common cancer in the world and its incidence is rising. Novel therapies targeting receptor tyrosine kinases, kinases and immune checkpoints have been employed with a significant improvement of the overall survival and long-term disease containment. Nevertheless, the disease often progresses and becomes resistant to the therapies. As such, the discovery of new targets and drugs for advanced melanoma still remains a difficult task. Gender disparities, with a female advantage in melanoma incidence and outcome, have been reported. Although emerging studies support the pro-tumorigenic role of androgen/androgen receptor axis in melanoma, the molecular bases of such evidence are still under intense investigation. We now report that ligand activation of the androgen receptor drives melanoma invasiveness and its escape from natural killer-mediated cytotoxic effect. By combining different experimental approaches, we observe that melanoma escape is mediated by the androgen-triggered shedding of the surface molecule MICA. Specific blockade of ADAM10 or androgen receptor impairs the androgen-induced MICA shedding and melanoma immune-escape. Further, the increase in MICA serum levels correlates with a poor outcome in melanoma patients treated with the anti-PD-1 monoclonal antibody, pembrolizumab. At last, melanoma cells depleted of the androgen receptor become more responsive to the most commonly used immunocheckpoint inhibitors, suggesting that the receptor dampens the immunotherapy efficacy. Taken together, our findings identify the androgen receptor as a diagnostic guidance in melanoma and support the repositioning of AR blockers in clinical management of patients.

## Introduction

Cutaneous melanoma is a malignant tumor arising from transformed melanocytes. Because of its increasing incidence and the high tendency to spread, melanoma is considered as the deadliest skin cancer. Patients with the occurrence of metastasis exhibit a 5-year survival rate of 23%, making metastasis the leading cause of melanoma-associated death [1].

Melanoma staging and therapy are based on biopsy and lymph-nodes evaluation. Surgical resection still represents the first-line approach for primary and/or surrounding lymph nodes-confined melanomas, with a success rate of almost 90%. Nevertheless, the treatment of the advanced disease might require several approaches including chemotherapy, immunotherapy, targeted therapy and radiotherapy, which have shown promising outcomes in efficacy and safety. Novel therapies targeting receptor tyrosine kinases (RTKs) or signaling effectors (B-RAF and MEK1/2) as well as immune checkpoints, including programmed death (PD)-1/PD-ligand (L)-1 pathway, cytotoxic T-lymphocyte-associated antigen (CTLA-4) and lymphocyte-activation gene (LAG)-3 have been recently used in metastatic melanoma patients, with a significant improvement in both the overall survival (OS) and long-term containment of disease [2]. Nevertheless, not all patients exhibit long-term response to these treatments and some of them develop resistance [3, 4].

Despite the recent efforts in identifying the potential factors affecting the progression and the phenotype of this malignancy, the question about the sex-related disparity in the disease’s incidence and outcome remains still pending. Although melanoma is not considered a classical sex hormone-responsive cancer, several evidence shows that gender represents an independent prognostic factor, with females exhibiting a higher survival rate [5]. Women are less subject to disease progression and often show thinner, less ulcered melanomas, frequently localized on lower extremities [6–11]. Again, women develop metastases later, as compared with men, and frequently exhibit only a local recurrence, still maintaining a survival advantage [8–10]. Nevertheless, the sex-related differences in terms of melanoma staging and OS are lost when women reach menopause, suggesting the protective role of female hormones in the disease’s progression [7–10].

Expression of the androgen receptor (AR) correlates with a poor prognosis in cutaneous melanoma patients and its over-expression promotes invasion in melanoma cultured cells and in vivo [12]. Sustained AR signaling promotes melanoma tumorigenesis [12, 13] and its ligand activation enhances the metastatic potential in melanoma cells and mouse model [14]. Genetic knockdown or pharmacologic inhibition of AR induces senescence and limits tumorigenesis in patient-derived specimens and melanoma cells from male and female patients [13]. Furthermore, AR antagonists improve the response to BRAF/MEK-targeted therapy in preclinical models of melanoma [15]. Thus, AR seems to play an oncogenic role in this disease and represents a disadvantage for therapy response and patients’ survival. The evidence so far collected, however, leaves still open the question about the intersection between AR and immune system in melanoma.

A complex network between melanoma and immune cells contributes to tumor progression and metastasis, and the high mutational burden of malignant melanoma makes it one of the most immunogenic cancers [16]. However, as melanoma cells have a great degree of plasticity, they can quickly develop escape mechanisms. Androgens inhibit and impair the inflammatory and immune response [17, 18]. The recent findings that AR antagonists promote the response to immune checkpoint inhibitors in castrate-resistant prostate cancer (CRPC) [19, 20] further support the role of AR in modulating the immune response.

Several cell types are involved in immune mechanisms. Among them, natural killer (NK) cells represent the cytotoxic arm of the innate immune system, with a widely recognized ability to target melanoma cells, especially the cancer stem cell (CSC) subpopulation, which is prone to metastasize [21, 22]. Relevant to our study, NK cells are more effective in limiting liver metastases of female mice, as compared with the male counterpart in a preclinical model of melanoma [23]. Thus, the ligand activation of AR might drive melanoma aggressiveness by promoting its invasion and NK immune-escape. The present study in melanoma patients and patient’s derived cells aims to answer these questions and provide new hints in melanoma progression and therapeutic guidance.

## Materials and methods

### Chemicals and cell culture

R1881 and bicalutamide (Sigma-Aldrich, St. Louis, MO, United States) were used at 10 nM and 10 μM, respectively. The FAK inhibitor defactinib (VS6063, Selleckchem, Munich, Germany), the Mitogen-activated protein kinase kinase (MEKK) inhibitor PD98059 (Selleckhem) and the selective ADAM10 inhibitor GI254023X (Selleckhem) were used at 10 μM. The Carbobenzoxy-Leu-Leu-leucinal MG132 (Selleckem) was used at 10 μM and pre-incubated for 6 h. Cell media and supplements were from Gibco (Thermofisher; Waltham, MA USA). Atezolizumab (1380723-44-3, TargetMol, Wellesley Hills, MA, United States), Pembrolizumab (1374853-91-4, MedChemExpress, Sollentuna, Sweden) and Ipilimumab (A2001, Selleckchem) were used at 5, 10 and 35 μg/ml.

The cells used throughout the manuscript were maintained at 37 °C in humidified 5% CO_2_ atmosphere and routinely monitored for Mycoplasma contamination and expression of sex steroid receptors, as reported [24]. Melanoma cells (Table 1), obtained from surgical specimens of patients with documented diagnosis of melanoma upon informed consent, were provided by IRCCS ‘Istituto Nazionale dei Tumori’, Milan (2009), Istituto Nazionale Tumori – IRCCS “Fondazione G. Pascale,” Naples (2018) and ‘San Raffaele’ University Hospital, Milan (2013). They were all established as primary short term cell cultures starting from tumor samples and cultured for no more than two weeks after thawing in RPMI-1640 medium, supplemented with penicillin (100 IU/mL), streptomycin (100 mg/mL) and 10% FBS. Seventy-two hours before stimulation, growing cells at 70% confluence were made quiescent using phenol red-free RPMI medium, containing 1% charcoal-stripped serum (CSS), penicillin (100 U/ml) and streptomycin (100 U/mL). Human prostate cancer-derived LNCaP cells, human breast cancer-derived MCF-7 and T47D from Cell Bank Interlab Cell Line Collection (ICLC, Genova, Italy) were cultured as previously reported [25].

**Table 1:** Cell lines and derivation.

Cell line	Derivation
MEL-CNF	Primary melanoma
WM115-4	Primary melanoma
MEL-CAL	Subcutaneous metastasis
AMM16	Lymph node metastasis
MEL35	Primary melanoma
MEL-STA	Primary melanoma
WM266-4	Lymph node metastasis
LCM-Mel	Lymph node metastasis
LCP-Mel	Primary melanoma
AMM8	Primary melanoma

### Melanoma patients

A total of 29 *naïve* stage IV melanoma patients (both female and male) were enrolled at Istituto Nazionale Tumori Fondazione “G. Pascale” of Naples (Italy), granted ethical permission. All the patients were treated with pembrolizumab (anti-PD1). Written informed consent was obtained from each patient, according with the Declaration of Helsinki. Blood samples from each patient were collected at the starting point and after three months of treatment. Table 2 summarizes the clinical characteristics of enrolled patients.

**Table 2:** Characteristic of pembrolizumab-treated stage IV melanoma patients.

Variables	*n* (%)
Number of patients	29
Median age	65.9 (38–87)
Sex	
Male	15 (51.7)
Female	14 (48.3)
M category	
M1a	11 (37.9)
M1b	6 (20.7)
M1c	8 (27.6)
M1d	4 (13.8)
LDH level	
Elevated	6 (20.7)
Normal	20 (69.0)
Not Available	3 (10.3)
Subsequent therapy	11 (37.9)
B-RAF status	
Wild type	22 (75.9)
Mutated	7 (24.1)
Median OS (months)	23.17 (1.10–62.50)
Median PFS (months)	22.33 (1.10–62.50)

### Enzyme-linked immunosorbent assay in patients’ sera and cell culture media

Blood-derived sera were used for MICA and MICB quantitative determination by enzyme-linked immunosorbent assay (ELISA). ELH-MICA-1 (RayBiotech, Peachtree Corners, GA, USA) and ELH-MICB-1 kits (RayBiotech) were used to quantify MICA and MICB, respectively. Quiescent AMM16 cells (1 × 10^6^ in in 100 mm plates) were left unstimulated or stimulated with R1881, in the absence or presence of the indicated compounds. After 6 h, cell culture media were collected and MICA or MICB were assayed according to the manufacturer’s instructions. Data were analyzed using the curve-fitting statistical software GraphPad Prism.

### 3D cultures in ECM

Melanoma cells (1.5 × 10^4^) were mixed with 250 µL of phenol red-free growth factor reduced Matrigel (10 mg/mL; BD Biosciences, Erembodegem, Belgium) and 50 µL of spheroid plating medium for each well in a 24 multiwell plate. Spheroid plating medium was made using phenol red-free DMEM/F12 medium, 3% CSS, B27 supplement (50× stock solution; Thermo Fisher Scientific), 100 U/mL penicillin, 100 U/mL streptomycin, GlutaMAX 100× (Gibco), 10 mM HEPES, 1 mM nicotinamide (Merck), 1.25 mM N-acetylcysteine (Sigma-Aldrich), and 10 µM Y-27632 (Merck-Millipore, Temecula, CA, USA). Spheroids were generated as reported [25, 26] and 3 days later, the spheroid plating medium was replaced with a similar medium, devoid of Y-27632 [26]. On 4th day, spheroids were left untreated or treated for additional 15 days with the indicated drugs. The medium was changed every 3 days. Different fields were analyzed using a Leica DMIRB (Leica) microscope equipped with C-Plan ×40 objective (Leica), and phase-contrast images were acquired using a DFC 450 C camera (Leica). The relative spheroid size was calculated using the Application Suite software (Leica) and expressed as a fold of increase over the basal spheroid size measured on the 4th day.

### Wound scratch, migration and invasion analyses; contrast-phase, immunofluorescence (IF) and confocal microscopy

In wound scratch analysis, 1.7 × 10^5^ cells were seeded in a 24-well plate, made quiescent, wounded using 10 µl sterile pipette tips and left un-stimulated or stimulated for the indicated times with R1881 (10 nM), in the absence or presence of the indicated compounds. Cytosine arabinoside (50 µM; Sigma-Aldrich) was included in the cell medium to avoid cell proliferation. Different fields were analyzed using DMIRB inverted microscope (Leica) equipped with N-Plan 10× objective (Leica). After 14 h h, phase-contrast images were captured using a DFC 450 C camera (Leica) and acquired using Application Suite Software (Leica). Images are representative of at least three different experiments. The wound gap was calculated using Image J Software and expressed as % of the decrease in the wound area [26]. Migration and invasion assays were done [27], using 3 × 10^4^ cells in collagen (migration)- or growth factor-reduced/phenol red-free Matrigel (invasion) precoated Boyden’s chambers (8 μm polycarbonate membrane; Corning; Corning, NY, USA). The indicated stimuli were added to the upper and the lower chambers. Cytosine arabinoside was included in the cell medium. After 7 h or 18 h, respectively, non-migrating or non-invading cells were removed from the membrane upper surface using a sterile cotton swab. Membranes were fixed for 20 min in 4% paraformaldehyde, stained with Hoechst, removed with forceps from the companion plate and mounted. Migrating or invading cells from at least 30 fields/each membrane were scored and quantified [27]. Data are representative of at least three different experiments.

The Cyto3D live–dead assay kit (TheWell Bioscience, North Brunswick, NJ, United States) was used to detect cell death in melanoma cells. Dead (stained with propidium iodide) and total (stained with acridine orange) cells were visualized by using a MICA-Leica Mycrosystems (Wetzlar, Germany) IF microscopy, equipped with PL FLUOTAR 10x/0,32 objective (Leica). The red/green mean fluorescence intensity corresponding to propidium iodide (Texas red) or acridine orange (green fluorescence protein; GFP), respectively was calculated using NIH Image J Software.

MICA staining by confocal microscopy was done in AMM16 cells. Cells on coverslips were fixed for 20 min with paraformaldehyde (3%, w/v, in PBS; Merck, Saint Louis, MO, United States) and incubated for 1 h with PBS containing foetal bovine serum (FBS; 1%, vol/vol). Coverslips were then incubated overnight with diluted (1:50 in PBS) rabbit polyclonal anti-MICA/B antibody (64899S, Cell Signaling, Danvers, MA, United States). Diluted (1:300 in PBS containing 0.01% BSA) fluorescein-conjugated AffiniPure anti-rabbit immunoglobulin G (IgG; Jackson ImmunoResearch Laboratories, West Grove, PA, United States) was used as secondary reagent. Nuclei were stained for 5 min with Hoechst 33258 (1 μg/ml; Merck). AR/ADAM10 co-localization in AMM16 was done by using diluted (1:70) rabbit Alexa Fluor 488-conjugate anti AR antibody (clone D6F11 from Cell Signaling; Danver, MA, USA) and diluted (1:50 in PBS) mouse monoclonal anti-ADAM10 antibody (sc-28358; Santa Cruz Biotechnology). Mouse antibody was detected using diluted (1:300 in PBS) goat anti-mouse Texas red-conjugated antibody (Jackson ImmunoResearch).

NKG2D staining was done in NK cells and images were acquired by confocal microscopy. NK cells fixed with paraformaldehyde (4%, w/v, in PBS), were permeabilized using Triton-X100 (0,1%, w/v Biorad in H_2_O) and incubated overnight with diluted (1:30 in PBS) rabbit polyclonal anti-NKG2D antibody (KLRK1-A11964, ABclonal, Düsseldorf, Germany). Diluted (1:200 in PBS containing 0,01%, w/v Triton and 1% w/v, FBS) goat anti rabbit Texas red-conjugated antibody (Jackson ImmunoResearch) was used as secondary reagent. Nuclei were stained for 5 min with Hoechst 33258 (1 μg/ml; Merck). Cells on coverslips (for MICA and AR/ADAM10 staining) or in IBIDI 8 well chamber (Ibidi, Lochhamer Schlag, Gräfelfing, Germany; for NKG2D staining) were analyzed using MICA-Leica Mycrosystems (Wetzlar, Germany) widefield and confocal microhub station, equipped with HC PL APO CS2 63.0 x NA 1.20 WD 220 water objective. Images were captured using MICA-Leica Mycrosystems and acquired using Leica Application Suite X (LASAX) software. The AR/ADAM10 co-localization ratio was analyzed using NIH Image J software.

### Small interfering (si) RNA experiments, lysates, western blot (WB), co-immunoprecipitation (Co-IP) and gelatine metallo-proteinase zymography

A pool of four target-specific 19–25 siRNAs (sc-29204, Santa Cruz Biotechnology, Dallas, TX, United States) was used in siRNA AR experiments. Non-targeting siRNA (ctrl siRNA; Santa Cruz Biotechnology), containing a scrambled sequence, was used as control. siRNA Alexa Fluor 488 was included to help identify the transfected cells. Lysate proteins were used in co-immunoprecipitation (Co-IP) experiments or electrophoretically separated by sodium dodecyl sulfate polyacrylamide gel electrophoresis (SDS-PAGE) [25]. The following antibodies were used in WB and Co-IP analyses: mouse monoclonal anti AR (441; Santa Cruz Biotechnology Dallas, TX, United States), anti PR (6A1; Cell Signaling, Beverly, MA, USA), anti tubulin (Sigma-Aldrich, St. Louis, MO, United States), anti FAK (610088 BD Bioscience, San Jose, CA, United States), anti P-Tyr397 FAK (611722 BD Bioscience), anti p-ERK (sc-7383; Santa Cruz Biotechnology), anti ERK (SC-1647; Santa Cruz Biotechnology), anti MT-MMP-1 (SC-373908; Santa Cruz Biotechnology), anti MT-MMP2 (SC-80213; Santa Cruz Biotechnology), anti ADAM-9 (sc-377233; Santa Cruz Biotechnology), anti ADAM-10 (sc-28358; Santa Cruz Biotechnology), anti ADAM-12 (sc-293225; Santa Cruz Biotechnology), anti MMP-1 (sc-21731 santa Cruz Biotechnology), anti MMP-7 (sc-515703 Santa Cruz Biotechnology), anti GAPDH (Elabsciences, Houston, Texas) antibodies; the rabbit anti ERα (HC-20; Santa Cruz Biotechnology), anti integrin β1 (Ab1952; Merck-Millipore, Saint Louis, MO, United States), anti MICA/B (64899S, Cell Signaling, Danvers, MA, United States), anti PD-1/CD279 (A11973, ABclonal Düsseldorf, Germany), anti PD-L1/CD274 (A1645, ABclonal) and anti-CD152/CTLA-4 (A24085, ABclonal) antibodies. The ECL system (GE Healthcare) was used to reveal immunoreactive proteins. Zymography assay in AMM16 cells was done as reported [25]. Full and uncropped western blots are presented in Supplemental Material.

### NK cells isolation and stimulation

Healthy donors PMBCs were isolated through Biocoll separating solution (Biochrom AG, Berlin, Germany) density gradient centrifugation. NK cells were then isolated using human NK Cell Isolation Kit (Miltenyi Biotec, Bergisch Gladbach, Germany). Their purity (>95%) was assayed by flow cytometry.

### Flow cytometry (FACS) analysis

Melanoma cells were firstly stained with Viobility 405/452 Fixable Dye (Miltenyi Biotec) to discriminate dead from live cells and then incubated at 4 °C for 30 minutes in the dark, using the following antibodies: FITC anti-human HLA-A,B,C (clone W6/32)/ IgG2a, APC anti-human CD274 (B7-H1, PD-L1; clone 29E.2A3)/ IgG2b, PE anti-human MICA/MICB (clone 6D4)/IgG2a, PerCP/Cy5.5 anti-human Galectin-9 (clone 9M1-3)/IgG1, APC anti-human CD155 (PVR) (clone SKII.4)/IgG1, PE anti-human CD112 (Nectin-2) (clone TX31)/IgG1 were from BioLegend (San Diego, California, USA); APC anti-human ULBP-1 (clone FAB1380A)/IgG2A, PE anti-human ULBP-2/5/6 (clone FAB1298P)/IgG2a, PE anti-human ULBP-3 (clone FAB1517P)/IgG2a were from R&D Systems (Minneapolis, Minnesota, USA); APC anti-human CD54 (ICAM-1) (clone HA58/IgG1, PE anti-human E-cadherin (clone 36/E)/IgG2a were from BD Bioscience. For NCR ligand detection, NKp30-Fc, NKp44-Fc and NKp46-Fc were obtained as described [28]. PE-conjugated F(ab′)2 goat anti-human IgG (Jackson ImmunoResearch Laboratories, Cambridgeshire, UK) was used as secondary antibody. The staining of NK cells was done using the following antibodies: APC CD56 (clone REA196)/IgG1, FITC CD3 (clone BW264/56)/IgG2a, PE CD152 (CTLA-4) (clone BNI3/IgG2a) from Miltenyi Biotec; BV510 CD3 (clone HIT3a)/IgG2a, BB700 CD56 (clone NCAM16.2)/IgG2b, PE-CF594 CD279 (PD-1) (clone EH12.1)/IgG1 from BD Bioscience. To discriminate dead lymphocites, BD Horizon™ Fixable Viability Stain 780 or 7-AAD (BD Bioscience) were used. After the staining, both melanoma cells and lymphocytes were washed with PBS and resuspended in FACS Flow. Data were acquired using FACS CANTO II or LSRFortessa™ (BD Biosciences). Throughout the experiments, isotype-matched controls were used to set up the negative values. Data were analyzed using FlowJo version 10 software analysis (Treestar US).

### Cytotoxicity assay

In cytotoxicity assay by FACS analysis [29], target cells were labeled with 150 mg/mL of fluorescent 5,6-carboxy-fluorescein-diacetate (CFDA)-mixed isomers (Invitrogen) for 30 min and then incubated in humidified 5% CO_2_ incubator at 37 °C in 96-well U-bottom plates with freshly purified allogenic NK effector cells at different effector:target (E:T) ratios. After 3 h of co-incubation in phenol red-free RPMI-1640 medium, cells were washed and fixed using BD CytoFix Fixation Buffer (BD Bioscience). Cells were washed with PBS and resuspended in FACS Flow (BD Biosciences). Target cell lysis was analyzed by flow cytometry using a FACSCANTO II and calculated according to the formula: % of specific lysis = (CT − TE)/CT ∗ 100, where CT indicates target cells’ mean fluorescence in control tubes and TE indicates target cells’ mean fluorescence in tubes containing effector cells.

Cytotoxic assay by colorimetric approach was performed by Cytotoxicity Detection Kit lactate dehydrogenase (LDH; 11 644 793 001, Roche). In melanoma-NK cells co-coltures, target cells were plated with freshly purified allogenic NK effector cells in phenol red-free RPMI-1640 medium at different E:T ratios in 96-well U-bottom plates. Co-coltures were untreated or treated with R1881 in the absence or presence of the indicated inhibitors and incubated in humidified 5% CO2 incubator at 37 °C. Six hours later, plates were centrifuged at 250 × *g* for 10 min. Supernatant were collected and transferred into an optically clear, 96-well, flat-bottom microplate. After 30 min of incubation with freshly prepared reaction mixture, LDH activity was determined by measuring the sample’s absorbance at 490 nm, using the multi-well reader (Enspire). Target cell lysis was calculated according to the formula: cytotoxicity (%) = effector − target cell mix − effector cell control) − low control/ (high control − low control) × 100.

Supernatants from siRNA ctrl- or siRNA AR-transfected melanoma cells were collected and transferred into an optically clear, 96-well, flat-bottom microplate. After 30 min of incubation with freshly prepared reaction mixture, LDH release was determined by measuring the sample’s absorbance at 490 nm, using the multi-well reader (Enspire).

### Melanoma-derived spheroids and co-coltures with NK cells

AMM16 cells (0.85 × 10^3^) were plated in each well of a 96 multiwell TC-plate, BIOFLOAT (Sarstedt, Numbrecht, Germany). Spheroids were grown in phenol red-free RPMI medium supplemented with 3% CSS, 100 U/mL penicillin, 100 U/mL streptomycin and GlutaMAX 100× (Gibco). At 3^rd^ day, spheroids were left untreated or treated for 10 days with the indicated compounds and medium was changed every 3 days. At 10^th^ day, the medium was replaced with phenol red-free RPMI medium. Spheroids were stained with Cyto3D® Live-Dead Assay Kit (The Well Bioscience, North Brunswick, USA) and NK cells were added (E:T ratio 25:1). Untreated or treated co-coltures were followed for 4 h. Images were acquired, before and after NK addition, using a MICA LEICA Mycrosystems (Leica) microhub microscope equipped with ×10 objective by confocal or thunder setting. The red/green mean fluorescence intensity corresponding to propidium iodide (dead cells) or acridine orange (total cells) respectively was calculated using NIH Image J Software.

### DNA isolation, polymerase chain reaction (PCR), sequencing and data analysis

Genomic DNA was extracted from 1 × 10^6^ AMM16, WM266-4 and MEL-CAL melanoma cells using QIAamp DNA Blood Mini kit (Qiagen, Hilden, Germany). The purity and concentration of DNA were measured by Qubit 2.0 (Thermo Fisher Scientific) using Qubit 1× dsDNA high sensitivity assay kit. hAR coding region (NM_000044) was amplified in 9 amplicons (between 300 and 900 nt in size). Each amplicon includes one exon, except for the exon 1 that was divided in 2 overlapping regions (1A and 1B), because of its big size (1616 nt). Primers amplify all the exons at the same PCR conditions, also including 20 nt at the splicing junctions (Table 3). The exon 1 needed a primer annealing exactly at its 5’ junction. PCR was done from 50 ng of the genomic DNA, using the AmpliTaqGold (Thermo Fisher Scientific).The 9 amplicons were analyzed by Sanger’s sequencing. PCR products were transferred to a BigDye Terminator v3.1 Cycle Sequencing Kit (Thermo Fisher Scientific) mixture for sequencing reaction, after Illustra ExoProStar 1-STEP Kit purification (GE Healthcare, Chicago, Il, USA), and run into a capillary electrophoresis system (3500xL Dx Genetic Analyzer; Thermo Fisher Scientific). Additional primers were designed to better define 3’ and 5’ junction of exon 1. Electropherograms of each amplicon were analyzed using Mutation Surveyor v5.1.0 (Softgenetics, State College, PA, USA). DNA sequences are deposited in unstructured repository (10.6084/m9.figshare.22550095.v1).

**Table 3:** Primers used.

Primer	Fw	Rev
AR_Ex1A	CGACTACCGCATCATCACAG	GCTCCAACGCCTCCACAC
AR_Ex1B	GCACTTCGACCATTTCTGAC	GAACACAGAGTGACTCTGC
AR_Ex2	GAGCAATGAATAATAGTCATTTATGCC	CTCTATTTCTGAGATGATAAAATCC
AR_Ex3	CTGTTCTAGAAATACCCGAAG	CTCTATTTCTGAGATGATAAAATCC
AR_Ex4	GTTGCATTGTGTGTTTTTGACC	GGTCCATAGGAGCGTTCAC
AR_Ex5	CAGGGACTCAGACTTAGC	CTTCACTGTCACCCCATCAC
AR_Ex6	GGATGGCAATCAGAGACATTC	GCTTTTCCCTAATAATGTTTTAATGG
AR_Ex7	GTGGTCAGAAAACTTGGTGC	GTGCCAGACTCTAGAGAAG
AR_Ex8	GACCAAAAATCAGAGGTTGGG	GAGGAGTAGTGCAGAGTTATAAC

### Data mining

RNA-sequencing and reverse-phase protein array data from the The Cancer Genome Atlas (TCGA) project were downloaded from UCSC Xena (http://xena.ucsc.edu/). Clinical information were obtained from the study “human skin cutaneous melanoma (SKCM)”. AR gene-expression analysis by RNA-Seq was performed by considering 481 specimens. Transcriptomic and survival analysis related to MICA, MICB and ADAM10 were derived from Gene Expression Profiling Interactive Analysis (GEPIA 2; http://gepia2.cancer-pku.cn/#index).

### Statistical analysis

All the experiments were done in triplicate and data are presented as mean ± standard deviation. The comparisons were all performed using the paired two-tailed Student’s *t* test on the GraphPad Prism 5.0 software. Spearman’s test was used to assess the correlation between sera concentration of MICB and survival in patients. Mean value of MICA serum concentration was calculated within the cohort and used as threshold to generate Kaplan–Meier (KM curves) to compare patients’ survival. The log-rank test was used to compare KM curves and calculate the *p*-value. The statistical computations were always done using the GraphPad Prism 5.0 software. *p*-values < 0.05 were considered statistically significant, throughout all the analyses.

## Results

### The ligand-mediated activation of AR impairs NK cell-mediated cytotoxicity against melanoma cells

**Fig. 1:**
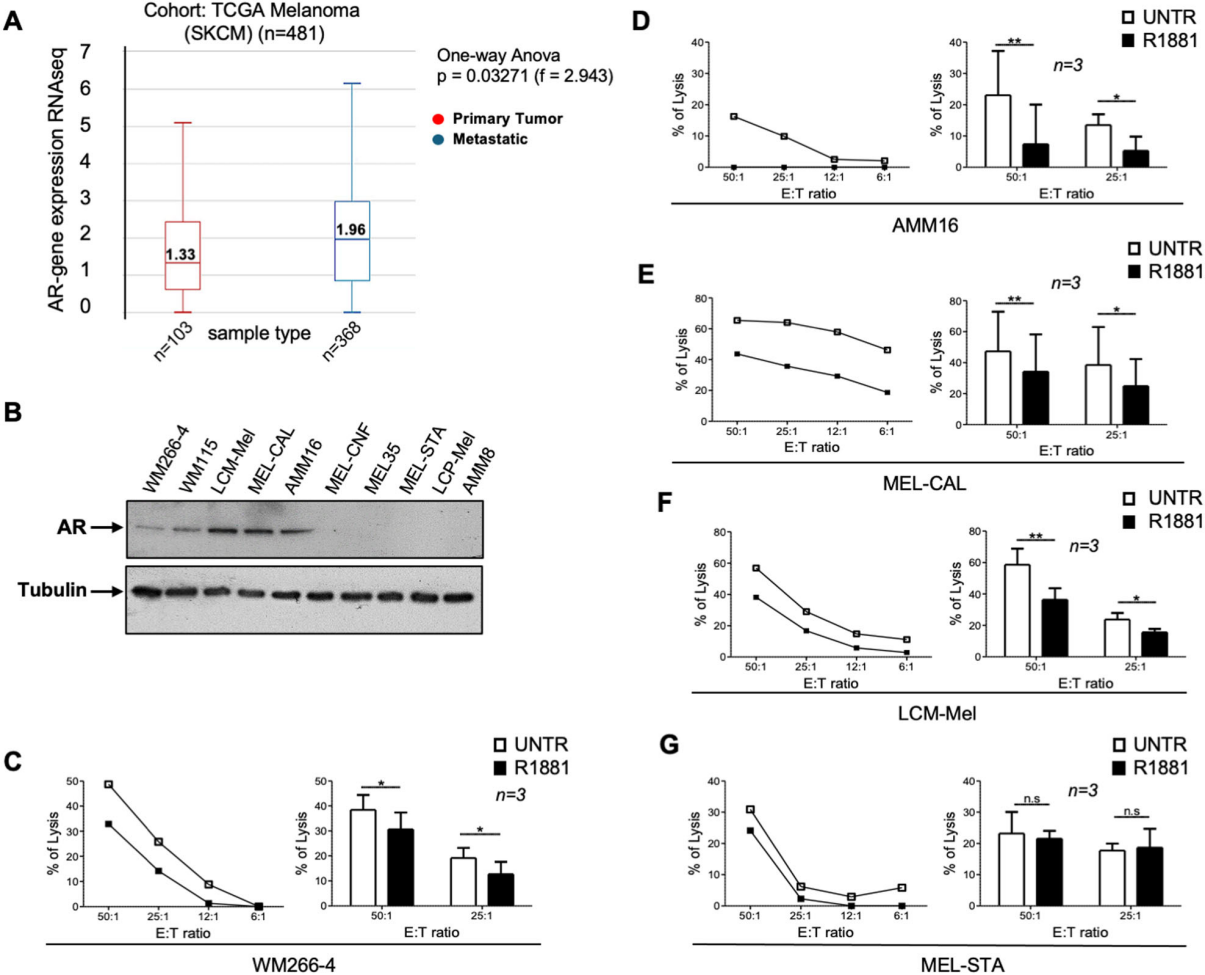
AR is expressed in metastatic melanoma and its ligand activation prevents NK cell-mediated killing. **A** AR gene expression levels in primary melanoma and in melanoma metastatic specimens (*n* = 481). AR was higher expressed in metastatic lesions (blu; *n* = 368; median 1.96) than in primary tumors (red; *n* = 103; median 1.33). One-way ANOVA with *p* = 0.03271 (f = 2.943). **B** WB analysis for AR in the indicated cell lines. Tubulin was revealed as a loading control. Representative experiment (left) and statistical analysis (right) of cytotoxicity assays done in untreated (white dots and bars) or 10 nM R1881-treated (black dots and bars) AR positive WM266-4 (**C**), AMM16 (**D**), Mel-CAL (**E**), LCM-Mel (**F**) and AR negative MEL-STA (**G**) cells with NK cells used at different E:T ratios. **C**–**G** Data from at least three independent experiments (*n* = *3*) were represented as means ± SD. **p-value < 0.01; *p-value < 0.05.

By interrogating the Genomic Data Commons (GDC) Cancer Genome Atlas (TGCA) SKCM for AR gene expression in primary and metastatic melanoma, we found that AR gene, analyzed by RNA-seq in a cohort of 481 patients, was more expressed in metastatic rather than primary tumor (Fig. 1A). WB analysis in a panel of melanoma-derived cells (Table 1) confirms that AR is prevalently expressed in cells derived from lymph node or subcutaneous metastasis (MEL-CAL, AMM16, WM266-4 and LCM-mel cells). With the only exception of WM115 cells, AR was almost undetectable in cells derived from a primary lesion (Fig. 1B and Supplementary Fig. S1A). Neither estrogen, nor progesterone receptors (ERα or PR, respectively) were detected in melanoma cells, while being expressed in MCF-7 or T47D cells (Supplementary Fig. S1B). At last, the Sanger sequencing indicates that AMM16, WM266-4 and MEL-CAL melanoma cells, which are mostly employed throughout this paper, harbor the wild-type version of AR (Supplementary Fig. S1C and data repository at 10.6084/m9.figshare.22550095.v1).

As NK cells less efficiently kill melanoma cells from male mice as compared with the female counterpart [23], we hypothesized that androgens interfere with NK cell functions. Therefore, we analyzed the cytotoxicity, using freshly purified resting allogenic NK cells as effectors and AR-positive WM266-4 (Fig. 1C), AMM16 (Fig. 1D), MEL-CAL (Fig. 1E) and LCM-Mel (Fig. 1F) melanoma cells as targets. Regardless the employed melanoma cells, 6h-androgen stimulation protected them against the NK-mediated killing (Fig. 1C–F). As cytotoxicity was unaffected by androgen-stimulation of AR-negative MEL-STA cells (Fig. 1G), we supposed the AR involvement in this response.

### MICA/B as putative targets in NK-cell mediated killing of melanoma cells

**Fig. 2:**
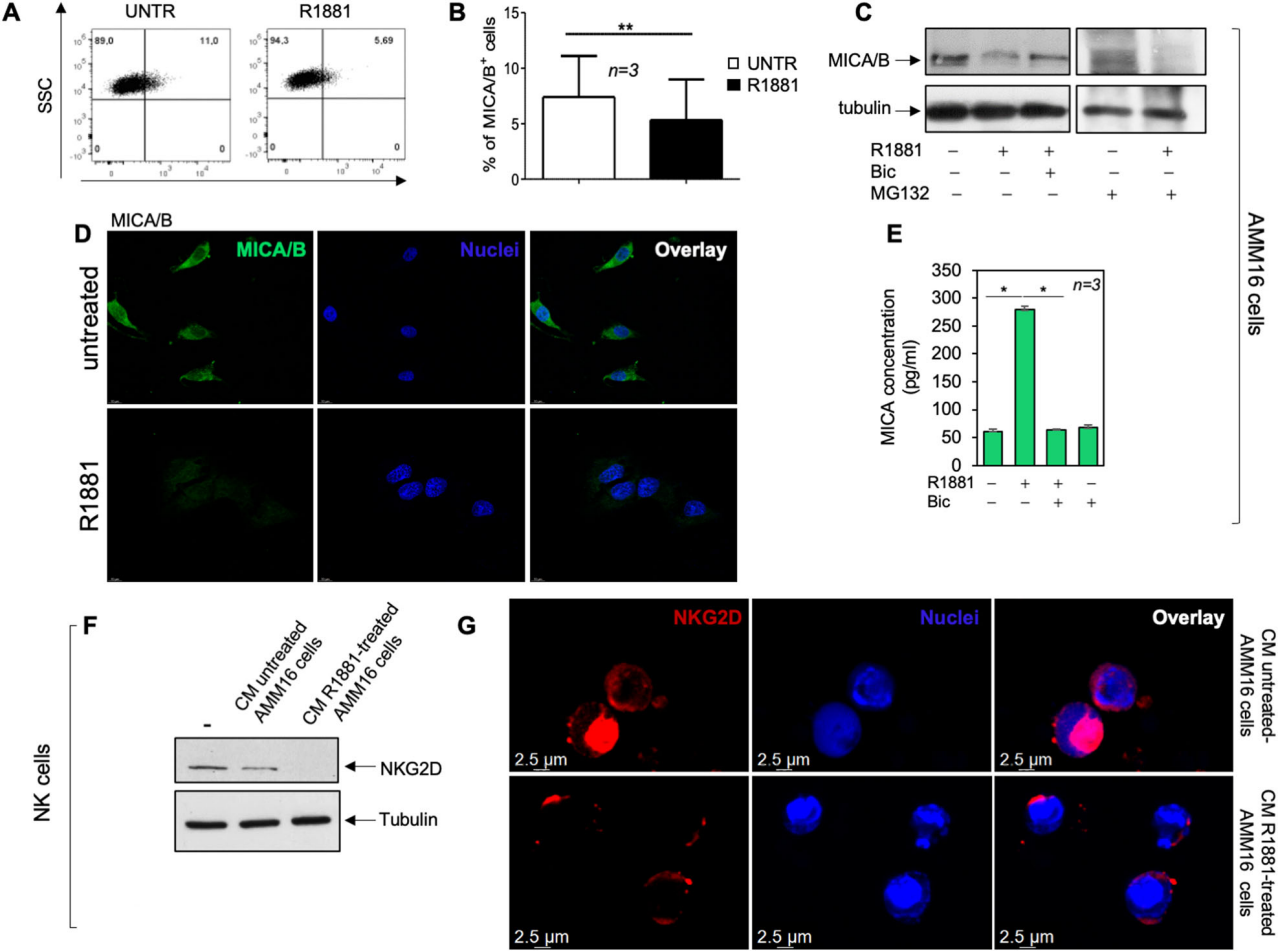
The androgen promotes the MICA/B shedding in melanoma cells, leading to the reduction of the NKG2D expression in NK cells. **A**, **B** Frequency of MICA/B in untreated (white bars) or R1881-treated (black bars) AMM16 cells. **B** Data from three independent experiments are represented as mean ± SD. *n* = 3; **p-value < 0.01. **C** AMM16 were untreated or treated for 6 h with R1881, in absence or presence of bicalutamide (Bic). When indicated, cells were pretreated with MG132. WB using the indicated antibodies was done. **D** Quiescent AMM16 cells on coverslips were left untreated or treated for 6 h with R1881. They were stained for MICA/B. Images captured by confocal microscope show the staining of MICA/B (green) and nuclei (blu). Merged images are presented in the right panels. They are representative of three independent experiments. Bar, 10 μm. **E** Cells were unstimulated or stimulated with R1881, in the absence or presence of Bic for 6 h. sMICA was assayed and quantified by ELISA. Graph is representative of three independent experiments; *p-value < 0.05. **F**, **G** NK cells were untreated or treated with AMM16 cells derived CM, as indicated. **F** WB with the antibodies against the indicated proteins was done. **G** NK cells were stained as reported in Methods section. Images captured by confocal microscopy show the staining of NKG2D (red) and nuclei (blu). Merged images are presented in the right panels. They are representative of three independent experiments. Bar, 2.5 μm.

To analyze the mechanism responsible for the androgen-induced NK immune escape, we firstly analyzed the expression of various surface molecules involved in NK cell activation and immune synapse formation in AMM16 cells (Supplementary Table 1S). Androgen challenge of these cells induces a significant reduction in both the frequency (Fig. 2A, B) and expression (Fig. 2C) of the major histocompatibility complex (MHC) class I chain-related protein A/B (MICA/B). The antiandrogen, bicalutamide reverts this effect (Fig. 2C), while the proteosome inhibitor MG132 [30] leaves unaffected the overall levels of MICA/B (Fig. 2C). Thus, pharmacological blockade of AR restores MICA/B expression and the ubiquitin-proteasome pathway is not involved in MICA/B reduction. Confocal microscopy analysis further confirmed these findings, as a deep and specific signal from MICA/B fluorescence is seen in the extra-nuclear compartment of untreated melanoma cells. Hormone treatment induces the loss of fluorescent signal (Fig. 2D). No fluorescence can be visualized in untreated AMM16 cells stained with FITC-conjugated secondary antibody alone, indicating the specificity of this approach (Supplementary Fig. S1D).

MICA/B undergoes shedding in melanoma cells [31]. As such, we analyzed the hormone effect on MICA/B release in AMM16 cells. MICA (Fig. 2E) and MICB (Supplementary Fig. S2A) are both released into the supernatants collected from untreated cells. Androgen stimulation significantly increases the MICA release, and bicalutamide reverts this effect (Fig. 2E). By contrast, hormone stimulation leaves unaffected MICB shedding (Supplementary Fig. S2A).

In its soluble form, the binding of MICA/B to the NKG2D receptor leads to its endocytosis and degradation by lysosomes. In such a way, the tumor immune surveillance can be blocked [32–34]. As in many advanced tumors, immune escape might be induced by MICA/B shedding and/or downregulation of NKG2D receptor in NK cells, we challenged NK cells with conditioned media (CM) derived from untreated or androgen-treated AMM16 cells and then analyzed the effect of soluble MICA on NKG2D expression. An overall reduction of NKG2D expression can be detected in NK cells treated with CM derived from androgen-treated melanoma cells. Treatment of NK cells with CM from untreated melanoma cells does not significantly modify NKG2D expression, as compared with the basal condition (NK cells in unconditioned medium; Fig. 2F). Further, we analyzed by confocal microscopy the expression of NKG2D on NK cell’s surface. Images in Fig. 2G reveal an intense ring-shaped structure corresponding to NKG2D staining in NK cells incubated with CM from untreated AMM16 cells. By contrast, a decrease in fluorescence can be visualized in NK cells treated with CM derived from androgen-challenged AMM16 cells. The absence of fluorescent signal from cells stained with Texas red-conjugated secondary antibody alone, indicates the specificity of our approach (Supplementary Fig. S2B). Thus, MICA shedding and NKG2D downregulation are involved in the androgen-induced immune escape of melanoma cells.

### Serum soluble MICA/B (sMICA/B) levels correlate with poor prognosis in advanced melanoma patients

**Fig. 3:**
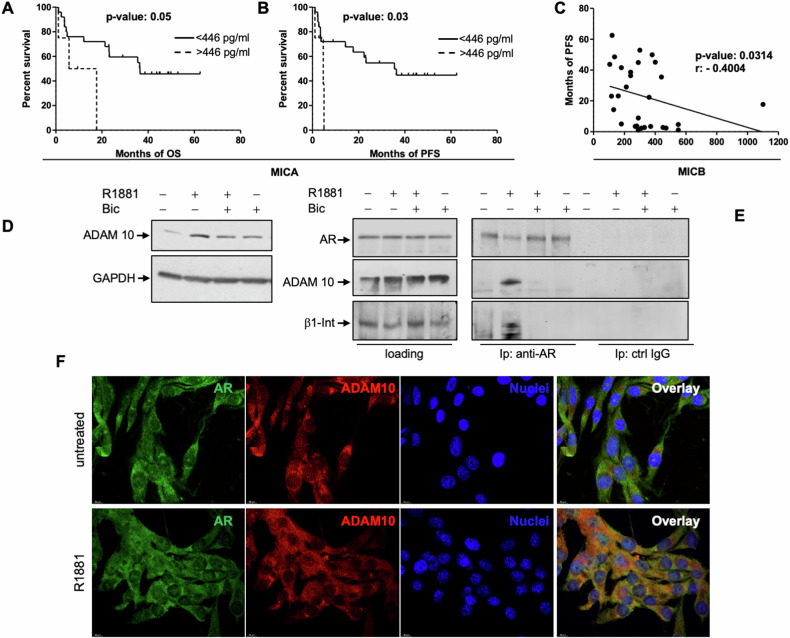
sMICA/B correlates with poor survival in melanoma patients. Androgens trigger ADAM10 activation and AR/β1 integrin/ADAM 10 complex assembly. **A**, **B** Variables correlating with survival in 29 Pembrolizumab (anti-PD1)-treated stage IV melanoma patients. MICA serum concentration was estimated by ELISA and expressed as pg/ml. The Kaplan–Meier curves for OS (**A**) and PFS (**B**) were generated. High and low levels of MICA correspond to dotted and solid lines, respectively. The mean value of MICA serum concentration (< or > of 438 pg/ml) was calculated on the entire cohort, chosen as cut-off and reported on the right of the panels. Log-rank test was used to compare curves. p-values are reported on the top of each panel. **C** Correlation between PFS and serum concentration of MICB in 29 Pembrolizumab (anti-PD1)-treated stage IV melanoma patients was calculated by Spearman correlation test. *p*-value and r coefficient are reported on the top of the panel. In **D**–**F**, AMM16 cells were employed and the presented results are representative of three different experiments. **D** Cells were unstimulated or stimulated for 6 h with R1881, in the absence or presence of bicalutamide (Bic). Lysate proteins were analyzed for ADAM10 expression. The filter was re-probed with anti-GAPDH antibody, as loading control. **E** Cells were unchallenged or challenged for 20 min with R1881, in the absence or presence of Bic. Lysate proteins were immune-precipitated using the anti-AR (anti-AR) or control (ctrl IgG) antibodies. WB analysis using antibodies against the indicated proteins was done to reveal co-immunoprecipitated proteins. **F** Cells on coverslips were left untreated or treated for 20 min with R1881 and, then, stained for AR and ADAM10. Images captured by confocal microscope show the staining of AR (green) and ADAM10 (red). Merged images are presented in the right panels. Bar, 10 μm. AR/ADAM10 co-localization ratio calculated by NIH Image J was 0.6 ± 0.06 in untreated cells and 3.11 ± 0.44 in R1881-stimulated cells. *p* < 0.05.

An increase in soluble forms of MICA/B (sMICA/B) has been detected in serum from melanoma patients, which correlates with poor survival in response to ipilimumab (anti-CTLA-4 mAb) treatment [35]. Therefore, we evaluated the correlation between sMICA/B with the OS and progression-free survival (PFS) in a cohort of 29 stage-IV melanoma patients treated with pembrolizumab (anti-PD-1 mAb). Kaplan–Meyer (KM) curves were generated using the mean values of sMICA as well as sMICB, calculated within the patients’ cohort, as cut-off. High levels of sMICA negatively correlate with both OS (Fig. 3A) and PFS (Fig. 3B). Additionally, Spearman test shows a negative correlation between serum levels of sMICB and PFS (Fig. 3C). These results are consistent with data set from GDC TGCA SKCM, showing that high levels of MICA correlate with poor OS (Supplementary Fig. S2C) and disease free survival (Supplementary Fig. S2D). They also show that MICA/B are higher expressed in melanoma specimens than normal tissues (Supplementary Fig. S2E, F).

### Androgen-induced MICA shedding in melanoma cells and NK cell-mediated cytotoxicity: the role of ADAM10

Membrane type matrix metalloproteases (MT-MMPs) and disintegrin-metalloproteinases (ADAMs) control MICA shedding [36–39]. Among MMPs or ADAMs analyzed in Supplementary Fig. S2G and Fig. 3D, the androgen/AR axis almost exclusively modulates ADAM10. Six-h hormone stimulation increases ADAM10 expression and bicalutamide reverts this effect (Fig. 3D). These findings are consistent with GDC TGCA SKCM data set (Supplementary Fig. S2H), showing that ADAM10 transcripts are higher in melanoma than in normal samples.

ADAMs contain a disintegrin domain, which represents a potential integrin ligand [40–43]. Given these and our previous findings on the androgen-induced AR/β1 integrin complex assembly in different cell types [24, 25, 44, 45], we analyzed the hormone effect on AR/ADAM10/β1-integrin complex assembly by Co-IP experiments in AMM16 cells. Irrespective of experimental conditions, similar amounts of AR, ADAM10 and β1 integrin were detected in loaded proteins (left section in Fig. 3E). The right section in Fig. 3E shows that 10 nM R1881 rapidly (within 20 min) and robustly stimulates the assembly of AR/ADAM10/β1 integrin complex, which is perturbed by bicalutamide addition. The antiandrogen does not affect the complex assembly when used alone, and the absence of eluted proteins in Co-IP done with control antibodies (ctrl IgG) confirms the specificity of our approach. We next used confocal microscopy analysis to support these data. Images in Fig. 3F show that androgens significantly (see the legend to Fig. 3) and rapidly (within 20 min) increase the AR/ADAM10 co-localization in the extra-nuclear compartment of AMM16 cells. Control images captured from melanoma cells stained with FITC-conjugated anti AR antibodies and Texas red-coupled secondary antibody indicate the specificity of our approach (Supplementary Fig. S3A).

**Fig. 4:**
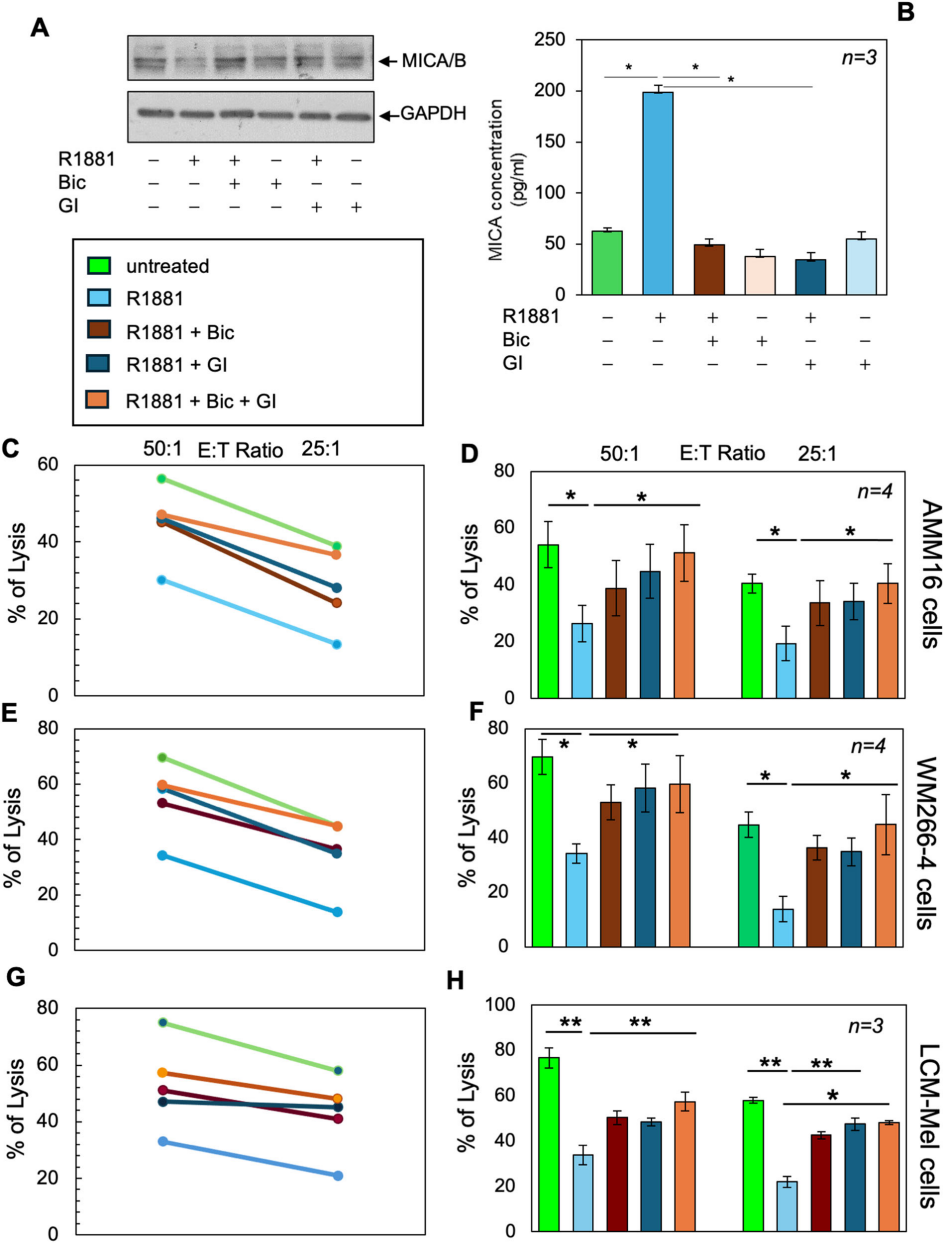
AR and ADAM inhibition impairs the MICA shedding and the androgen-induced melanoma escape from NK cells. **A**, **B** Cells were unstimulated or stimulated for 6 h with the indicated compounds. **A** Lysate proteins were analyzed for MICA/B expression using the anti-MICA/B antibody. The filter was re-probed using anti-GAPDH antibody, as loading control. **B** sMICA was quantified by ELISA. Data from three (*n* = *3*) different experiments are expressed as mean ± SD; *p < 0.05. Representative experiment (**C**, **E**, **G**) and statistical analysis (**D**, **F**, **H**) of cytotoxicity assays performed in untreated or treated AMM16 (**C**, **D**), WM266-4 (**E**, **F**) and LCM-Mel (**G**, **H**) cells co-coltured for 6 h with NK cells used at different E:T ratios (50:1; 25:1). **D**, **F**, **H** Data derived from different experiments (*n* = 3) or (*n* = *4*) are expressed as mean ± SD; *p < 0.05, **p < 0.01.

We next analyzed the effect of the ADAM-specific hydroxamate-based MMP inhibitor, GI254023X, [46] on MICA shedding and its related implications. GI254023X efficiently inhibits the androgen-induced MICA shedding as shown by WB analysis of lysate proteins (Fig. 4A) and ELISA assay of supernatants (Fig. 4B) from AMM16 cells. The inhibitor leaves unaffected MICB release (Supplementary Fig. S3B), thus indicating that the hormone-induced AR/ADAM10 complex only controls MICA shedding.

**Fig. 5:**
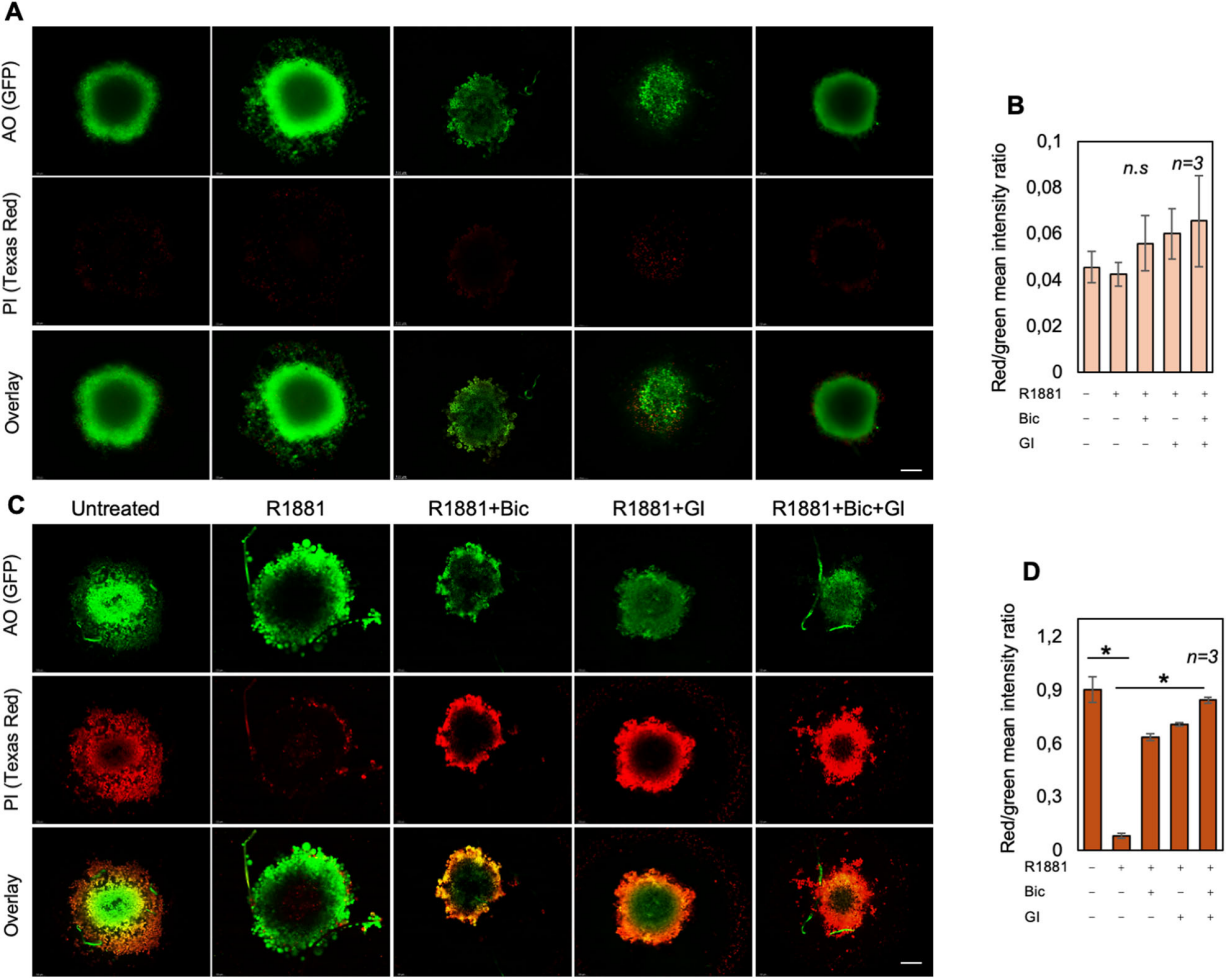
AR and ADAM inhibition impairs the androgen-induced melanoma escape from NK cells. **A**, **C** AMM16-derived spheroids were untreated or treated as indicated in the figure and stained. Images of spheroids before (**A**) and after (**C**) the NK cells addition are presented. The green fluorescence from the acridin orange (AO) staining indicates total cells, while the red fluorescence from the propidium iodide (PI) staining indicates dead cells. **C** Spheroids were co-coltured for 4 h with NK cells. Images are representative of three different experiments. Bar, 100 µm. **B**, **D** The graphs represent the dead cells/total cells. Values of dead and total cells were analyzed using NIH Image J. They derive from red fluorescence mean/green fluorescence mean intensity and are expressed as mean ± SD of 3 different experiments (*n* = 3); *p < 0.05.

We next verified whether ADAM10 is involved in immune-escape of melanoma cells. Therefore, we performed cytotoxicity assays using freshly purified resting allogenic NK cells as effectors, and AMM16 (Fig. 4C, D), WM266-4 (Fig. 4E, F) or LCM-MEL (Fig. 4G, H) melanoma cells as targets. As in Fig. 1C–F, 6 h androgen stimulation protects melanoma cells against the NK cell-mediated killing. Bicalutamide or GI254023X partially restore the cytotoxicity when used alone, while their combination (bic *plus* GI) potentiates this effect (Fig. 4C–H). Findings in AMM16 melanoma-derived spheroids further reinforce these data. Untreated or treated spheroids stained with live-dead reagent were then visualized by confocal microscopy before (Fig. 5A) or after (Fig. 5C) NK cell’s addition. In the absence of NK cells (Fig. 5A), or after only few minutes of their addition (Supplementary Fig. S3C), the mean intensity of red fluorescence from dead-cells is similar between the various experimental points. By contrast, it significantly increases after 4 h of co-colture with NK cells and results lower in the androgen-treated co-coltures (Fig. 5C), supporting the role for androgen/AR axis in the control of NK cell activity and melanoma aggressiveness.

### ADAM10 controls the androgen-induced migration, spheroids-size growth and signaling activation in melanoma cells

**Fig. 6:**
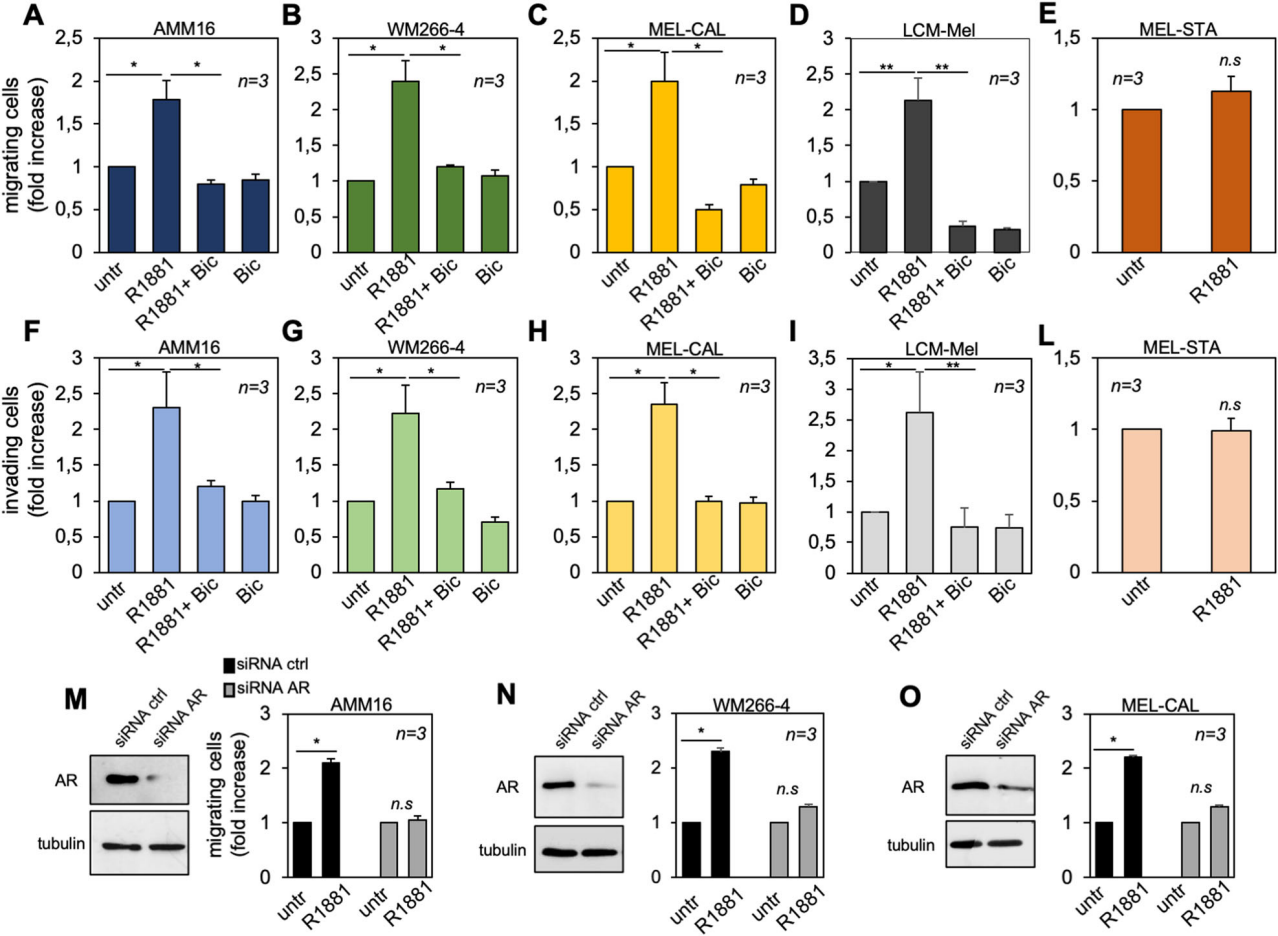
AR ligand-stimulation promotes migration and invasiveness of melanoma cells. AMM16 (**A**, **F**), WM266-4 (**B**, **G**), MEL-CAL (**C**, **H**), LCM-Mel (**D**, **I**) and MEL-STA (**E**, **L**) cells were left unchallenged or challenged with R1881, in the absence or presence of Bicalutamide (Bic). Cells were allowed to migrate (**A**–**E**) or invade (**F**–**L**), counted and data are expressed as fold increase. AMM16 (**M**), WM266-4 (**N**) and MEL-CAL (**O**) cells were transfected with siRNA Alexa Fluor 488 along with control siRNA (siRNA ctrl) or siRNA AR (siRNA AR). Lysate proteins were analyzed by WB using the indicated antibodies (left panels in **M**–**O**). Cells were left in the absence or presence of R1881 and then allowed to migrate. Migrating cells were scored and data expressed as fold increase. In **A**–**O**, data are expressed as mean ± SD of 3 different experiments (*n* = 3); *p < 0.05; **p < 0.01.

Tumor cell migration is associated with immune cell infiltration, immune escape and metastasis [47]. Therefore, we firstly analyzed the role of the androgen/AR axis in motility and invasiveness of melanoma cells. Androgen promotes AMM16 (Supplementary Fig. S4A), WM266-4 (Supplementary Fig. S4B) and MEL-CAL (Supplementary Fig. S4C) cell migration in wound scratch assay, while bicalutamide reverts the hormone effect. Quantitative data in Fig. S4 (panels D-F) show similar results. We further confirmed the hormone effect on migration (Fig. 6A–D) and invasiveness (Fig. 6F–I) of AR-expressing AMM16, WM266-4, MEL-CAL and LCM-Mel cells. Bicalutamide inhibits the androgen effects, leaving unaltered the migration or invasion of melanoma cells when used alone. Neither migration (Fig. 6E), nor invasiveness (Fig. 6L) are detected upon androgen-stimulation of AR-negative MEL-STA cells, pointing to AR requirement for these hormone effects. Subsequent experiments confirmed this issue, as depletion of AR by siRNA abolishes the androgen effect on AMM16 (Fig. 6M), WM266-4 (Fig. 6N) and MEL-CAL (Fig. 6O) cell motility, as compared with cells transfected with control siRNA.

**Fig. 7:**
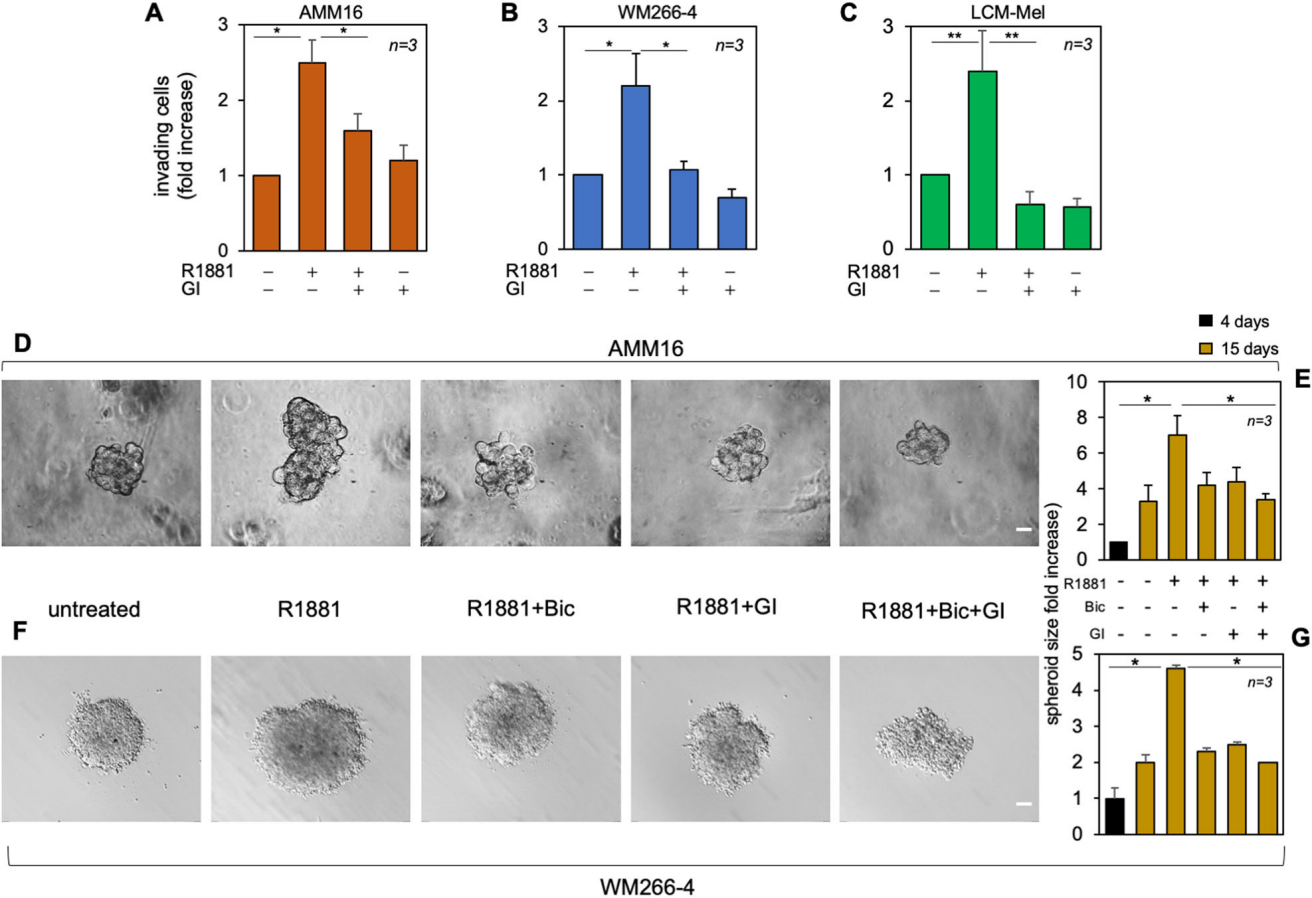
AR and ADAM10 mediate the androgen-triggered invasiveness and growth of melanoma-derived spheroids. **A** AMM16, **B** WM266-4 and **C** LCM-Mel cells were used for invasion assays. The indicated compounds were added to the upper and the lower chambers and cells were counted. Results from three different experiments were collected and expressed as fold increase. *p < 0.05; **p < 0.01. **D** AMM16- or **F** WM266-4 derived spheroids were generated by Matrigel embedding. After 4 days, spheroids were left untreated or treated with the indicated compounds for additional 15 days. Phase-contrast images captured at the 15th day are shown. They are representative of 3 different experiments. Bar, 100 μm. **E**, **G** Spheroid size was calculated using the Leica Suite software in basal conditions (4 days, not shown) or in cells unstimulated or stimulated for 15 days as indicated. Data are expressed as fold increase. In **A**–**C**, **E**, **G**, means and SD are shown. *n* represents the number of experiments. *p < 0.05; **p < 0,01.

As ADAM10 belongs to the MMP family [48], we next investigated the role of its proteolytic activity in the androgen-driven motility and spheroid growth in extracellular matrix (ECM). GI254023X inhibits the androgen-triggered motility (Supplementary Fig. S5A) and invasiveness (Fig. 7A–C) in the indicated melanoma cells. Bicalutamide or GI254023X or a combination of both dampen the androgen-induced increase of AMM16 (Fig. 7D) or WM266-4 (Fig. 7F) spheroid size, with a slight improvement of their inhibitory capacity when used in combo (Fig. 7E, G). In summary, pharmacologic blockade of AR or ADAM10 impairs melanoma-derived spheroid growth and inhibits the invasive phenotype induced by androgens in melanoma cells.

**Fig. 8:**
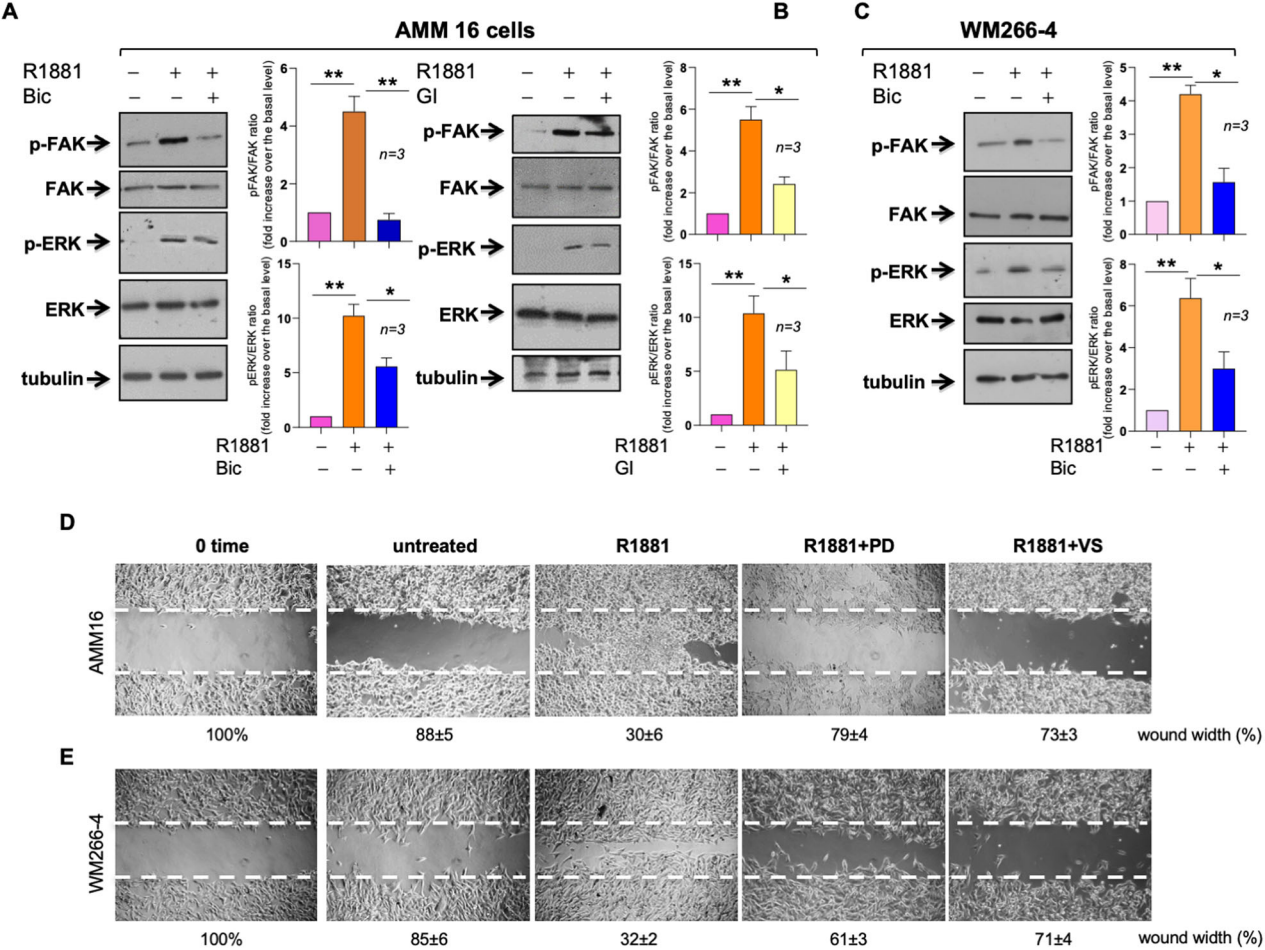
Signaling effectors involved in the androgen-mediated migration of melanoma cells. AMM16 (**A**, **B**) or WM266-4 (**C**) cells were left unchallenged or challenged for 20 min with R1881 in the absence or presence of the indicated compounds. Lysate proteins were analyzed by WB using the antibodies against the indicated proteins. p-FAK stands for Tyr397 p-FAK; p-ERK stands for p44 and p42 ERK. The filters were re-probed using anti tubulin antibody, as a loading control. **A**–**C** Expression levels of the proteins were analyzed by densitometry analysis, using NIH Image J Software. Graphical analysis, representative of three different experiments (*n* = *3*), is shown in the right sections. Means and SD are shown. *p < 0.05; **p < 0.01. AMM16 (**D**) and WM266-4 (**E**) cells were wounded and left untreated or treated with R1881, in the absence or presence of the indicated compounds. Phase contrast images are representative of three different experiments, each in triplicate. The wound area was calculated using Leica Suite software and data are expressed as the percentage of wound width over the control cells (analyzed at time 0).

When we analyzed the activity of the main signaling effectors involved in motility and adhesion, we observed that hormone stimulation rapidly triggers activation of the focal adhesion kinase (FAK) and extracellular-regulated kinase (Erk) in AMM16 (Fig. 8A, B) and WM266-4 (Fig. 8C) cells. Bicalutamide (Fig. 8A, C) and GI254023X (Fig. 8B) revert this effect, further supporting a role for ADAM10 in the androgen-induced motility of melanoma cells. As the FAK inhibitor, VS-6063 [49], and the MEK-1 inhibitor, PD98059 [50], impair the androgen-triggered motility in wound scratch assay (Fig. 8D, E), we concluded that the androgen/AR axis engages and activates the two effectors to drive melanoma cell locomotion.

### AR knockdown fosters the melanoma cell death in response to immune-checkpoint inhibitors (ICIs)

**Fig. 9:**
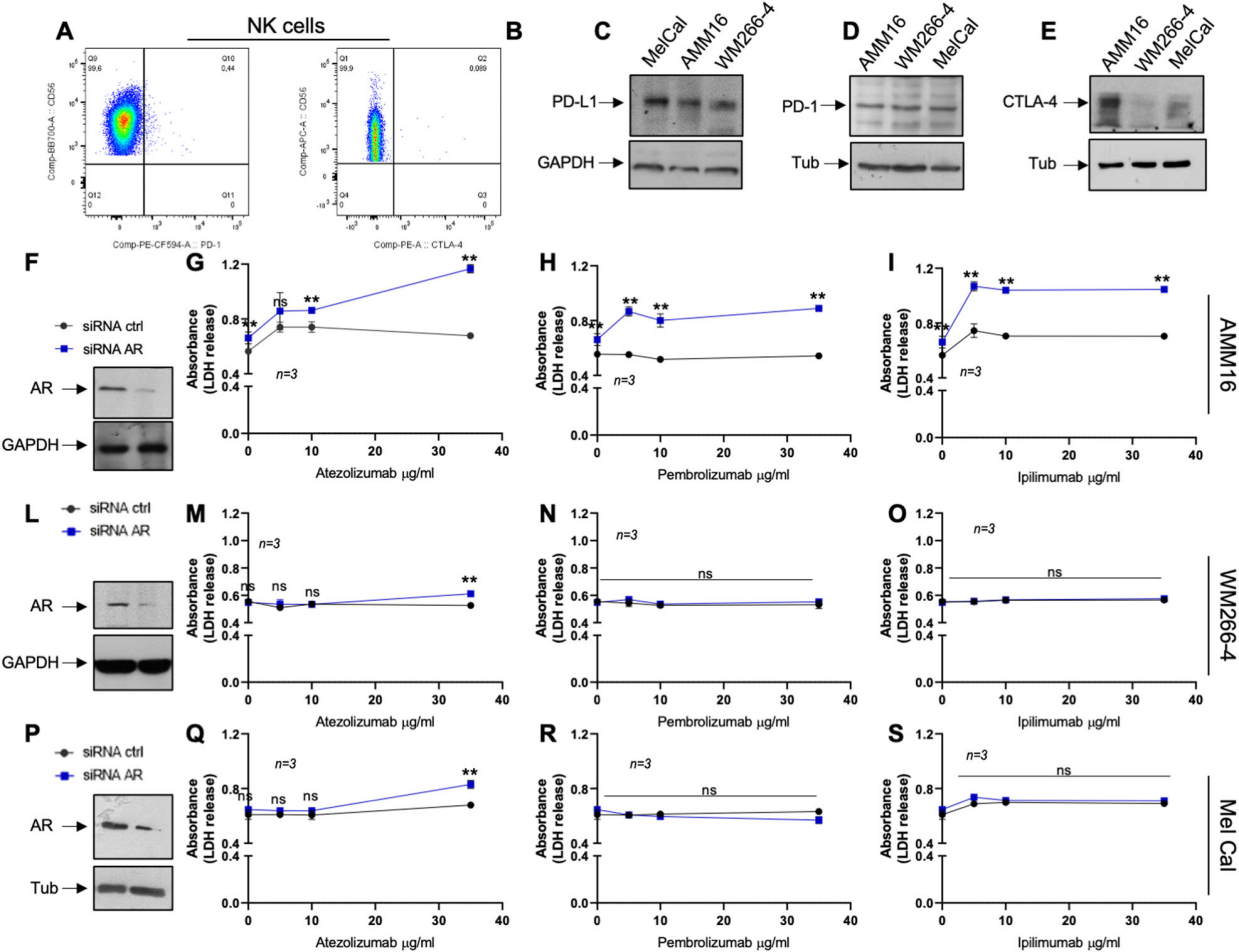
The effect of immune-checkpoint inhibitors (ICIs) on cell death of AR-depleted melanoma cells analyzed by LDH release determination. Surface expression of PD-1 (**A**) and CTLA-4 (**B**) on NK cells, identified as CD56^+^ CD3^−^ cells. WB analysis for PD-L1 (**C**), PD-1 (**D**) and CTLA-4 (**E**) expression in the indicated melanoma cell lines. Tubulin or GAPDH were revealed as loading controls. AMM16 (**F**), WM266-4 (**L**) and MEL-CAL (**P**) cells were transfected with control siRNA (siRNA ctrl) or AR siRNA (siRNA AR). Lysate proteins were analyzed by WB using the indicated antibodies. Melanoma cells were untreated or treated with 5, 10 or 35 μg/ml of Atezolizumab (**G**, **M**, **Q**), Pembrolizumab (**H**, **N**, **R**) or Ipilimumab (**I**, **O**, **S**) for 26 h. LDH release was determined as indicated in Methods section. In **G**–**I**, **M**–**O**, **Q**–**S**, data are expressed as mean ± SD of 3 different experiments each in triplicate; *p < 0.05; **p < 0.01.

**Fig. 10:**
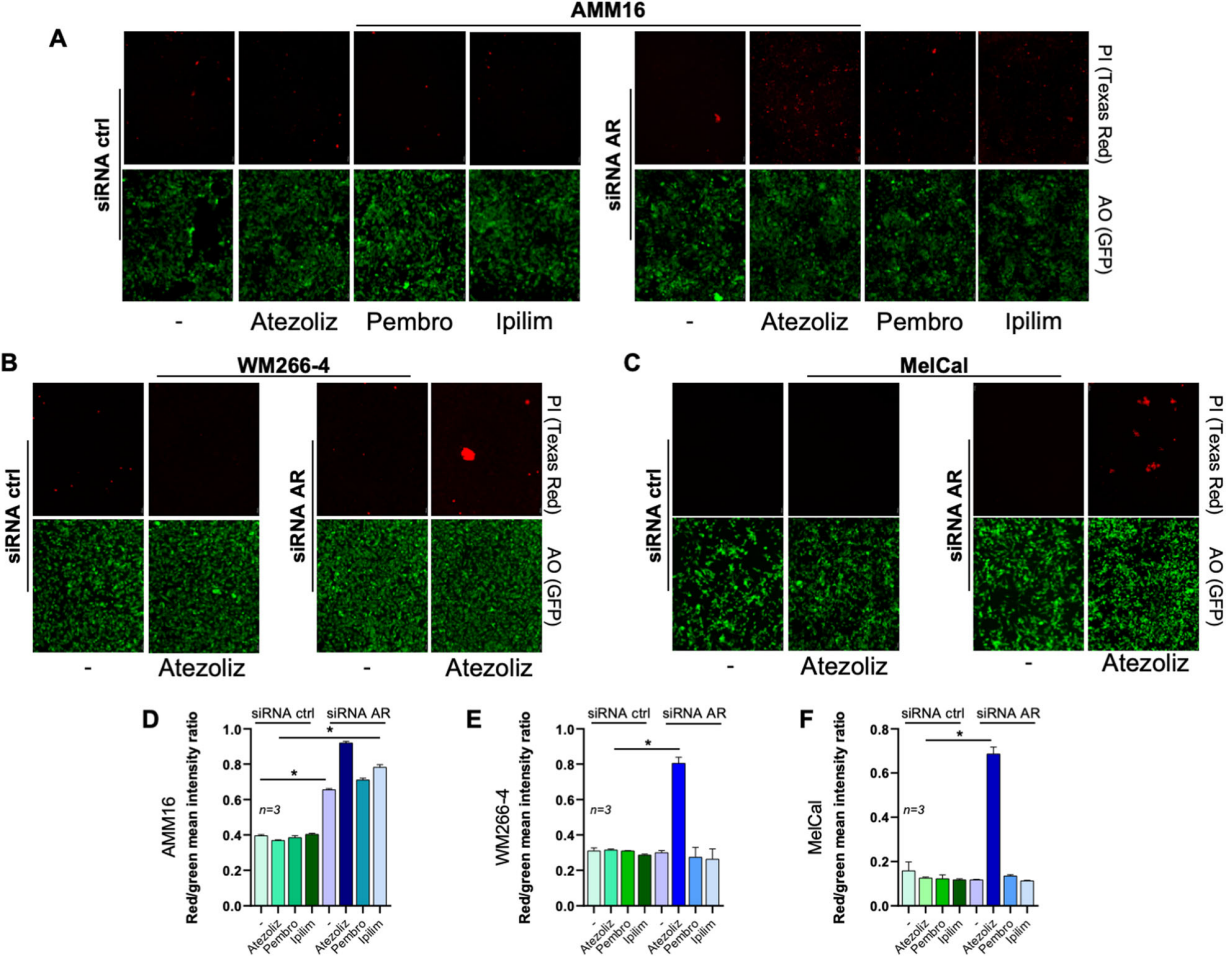
The effect of immune-checkpoint inhibitors (ICIs) on cell death of AR-depleted melanoma cells analyzed by Immunofluorescence. AMM16 (**A**, **D**), WM266-4 (**B**, **E**) and MelCal (**C**, **F**) cells were transfected with control siRNA (siRNA ctrl; left panels in **A**–**C**) or AR siRNA (siRNA AR; right panels in **A**–**C**) and treated with the indicated ICIs at 35 μg/ml. The red fluorescence from the propidium iodide (PI) and the green fluorescence from the acridin orange (AO) staining indicate dead and total cells, respectively. Images are representative of three different experiments. **D**–**F** The graphs represent the dead cells/total cells analyzed using NIH Image J. They derive from red fluorescence mean/green fluorescence mean intensity and are expressed as mean ± SD of 3 different experiments (*n* = 3); *p < 0.05.

Many findings have linked steroid receptors with inflammation, immune-responses and immunotherapy efficacy [51]. Therefore, we firstly analyzed expression of immune-checkpoints in NK and melanoma cells. As reported [52], FACS analysis in Fig. 9A, B confirms that resting NK cells do not express PD-1 and CTLA-4. MEL-CAL, AMM16 and WM266-4 melanoma cells express both PD-L1 (Fig. 9C) and PD-1 (Fig. 9D). Albeit at different extent, AMM16 and MEL-CAL cells only express CTLA-4 (Fig. 9E). These findings are consistent with previous data [53–57]. As such, ICIs cannot modulate NK cell cytotoxicity in co-culture setting. Therefore, we investigated the impact of AR in melanoma responsiveness to immunocheckpoint blockade. AMM16 (Fig. 9F), WM266-4 (Fig. 9L) and MEL-CAL (Fig. 9P) cells, depleted or not of AR, were treated with increasing concentrations of anti-PD-L1 (Atezolizumab; Fig. 9G, M, Q), or anti-PD1 (Pembrolizumab; Fig. 9H, N, R), or anti-CTLA4 (Ipilimumab Fig. 9I, O, S). Cell death triggered by each of these treatments was assayed by measuring the LDH release [58]. Although AR depletion already induces cell death in AMM16 cells, all the employed ICIs enhance this effect (Fig. 9G, H, I). A different scenario was detected in WM266-4 (Fig. 9M–O) and MEL-CAL (Fig. 9Q–S) cells, as the LDH release only increases in cells devoid of AR and treated with 35 μg/ml Atezolizumab (Fig. 9M, Q). These results were confirmed using a live–dead assay in IF. AR knockdown induces, indeed, cell death in AMM16 (Fig. 10A, D), but not in WM266-4 (Fig. 10B, E) and MEL-CAL (Fig. 10C, F) cells. Atezolizumab (at 35 μg/ml) increases the cell death in all the AR-depleted cells (Fig. 10A–F), while pembrolizumab and ipilimumab are only effective in AMM16 cells (Fig. 10A, D).

In summary, these and other findings in our study indicate that AR blockade by antagonists or somatic knockdown improves the responsiveness of melanoma cells to MMPs inhibitors or immunotherapeutic agents. As such, attenuation of melanoma invasiveness and immune escape can be observed, together with an increase in cell death.

## Discussion

Gender-related differences in melanoma incidence and outcome are well established [59], with a survival disadvantage among men [60–62]. Much evidence supports, indeed, a role for androgens in melanoma proliferation, invasiveness and drug-resistance [12–15]. Additionally, men display a less effective immune surveillance and a reduced ability to suppress the mutation-rich population of melanoma cells [59]. Therefore, it might be argued that androgens regulate the “immune fitness” of melanoma. Nevertheless, the underlying molecular aspects still remain debated. The present study aims to dampen this gap.

We now report that melanoma cells derived from subcutaneous or lymph node metastasis express the wild-type human AR, as indicated by biochemical and genetic analyses. By interrogating the GDC TGCA SKCM data set, we detect a correlation between increased expression of AR and metastatic disease. Our findings consistently show that AR is more expressed in metastatic melanoma cells as compared with those obtained from primary lesions. In addition to supporting a role for AR in melanoma progression, our results are consistent with previous findings showing that genetic or pharmacological manipulation of AR limits tumorigenesis, triggers senescence [13] and improves the response to B-RAF/MEK inhibitors [15] in preclinical and clinical melanoma models.

**Fig. 11:**
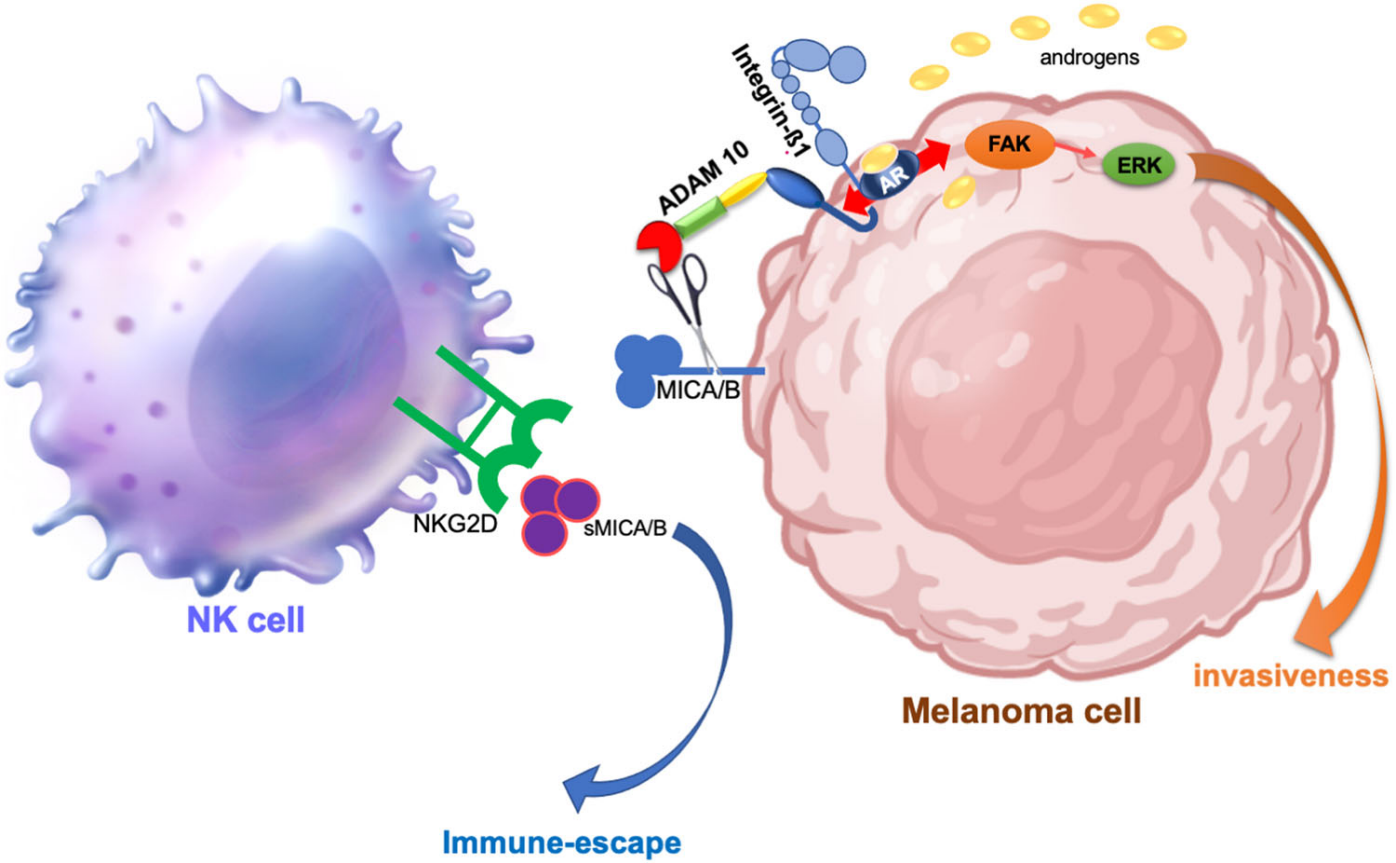
Androgen promotes melanoma immune-escape by NK cells and invasiveness: graphical representation. Androgen stimulation of melanoma cells triggers the assembly of the AR/ADAM10/β1 integrin complex. Such complex enables ADAM10 to act as MICA sheddase, on one hand. The MICA shedding, hence, causes the effective masking from NKG2D recognition allowing the immune-escape. On the other, the complex mediates the androgen-induced invasiveness of melanoma cells through the activation of FAK and ERK.

Further, we have explored the influence of androgens in the susceptibility of melanoma cells to NK cell recognition. Our results show that androgens reduce frequency and expression of the surface NKG2D ligand MICA/B in melanoma cells, with a simultaneous increase in sMICA levels. As such, down-regulation of NKG2D receptor surface expression on NK cells follows. As a net result, the resistance of melanoma cells to NK cell attack is enhanced. AR blockade by bicalutamide counteracts these effects, indicating that the reduction in MICA/B expression is consequent to the ligand-activation of AR. Consistent with results from GDC TGCA SKCM data base, we observe a correlation between high MICA/MICB serum levels and poor OS as well as PFS in a cohort of patients with advanced melanoma. Our and other findings [63–66] indicate that sMICA represents a valuable prognostic factor and a clinically actionable target [67–69] in many human cancers, including melanoma. Despite these clinical findings, the mechanism of MICA release by human cancer cells is not completely understood. We now observe that ADAM10 acts as sheddase of melanoma cell–associated MICA. Specific blockade of ADAM10 by GI254023X inhibits the hormone effect on MICA expression in melanoma cells and prevents its release, thus counteracting the androgen-induced immune escape. Additionally, hormone stimulation of melanoma cells increases the expression and activation of ADAM10. Findings on hormone regulation of ADAM10 are not completely novel, as androgen upregulation of ADAM mRNA has been previously reported in prostate cancer cells [70, 71]. Nonetheless, our study provides the first evidence of a direct relationship between androgens and MICA/B shedding by ADAM10 in melanoma, which is classically considered a ‘non-hormone dependent cancer’. Androgens rapidly and significantly enhance the assembly of a complex between AR/ADAM10/β1-integrin, as shown by Co-Ip experiments. Such complex activates ADAM10, thus driving MICA shedding, on one hand. On the other, experiments with GI254023X inhibitor strongly support the role of ADAM10 in the androgen-enhanced invasion of melanoma cells and size of derived spheroids (Fig. 11). Thus, ADAM10 might act as a crossroad in the hormonal control of immunosurveillance and invasion of melanoma cells. The finding that blockade of AR by somatic knockdown or antagonists reverse the androgen-elicited effects on melanoma cell locomotion and 3D models, further reinforce the role of receptor in the androgen signaling leading to melanoma aggressiveness.

Although immunotherapy represents a great promise in the treatment of melanoma,this approach is not always successful, especially among males that often acquire resistance to immunotherapy. Patient’s gender influences, indeed, the efficacy of immunotherapy in many solid tumors [72, 73], and combination of AR antagonist, enzalutamide and anti-PDL1 mAb exerts a remarkably antitumor effect in mouse model of castrate-resistant prostate cancer [19]. Although these findings suggest a role for AR in anticancer immunomodulation, many questions remain still unaswered. Although innate differences in estrogen- and androgen-activated pathways have been so far identified as mechanism of gender-related disparity to immunocheckpoint blockade in melanoma [74, 75], the molecular basis of the different outcome is still under investigation. However, it might be argued that while the complex network engaged by steroid receptors (AR, ER alpha or beta, PgR and others) has been deeply investigated in sex-hormone responsive malignancies, it remains still pending in melanoma. Further, driving factors of the sexual dimorphism in treatment outcomes are certainly attributable to the impact of steroid receptors in inflammation [51] or variations in the immune background [76, 77], tumor microenvironment [25, 78] and cell susceptibility to immune therapies [79].

Findings reported in this paper show that depletion of AR positively impact the efficacy of ICIs in melanoma cells, as the anti-PD-L1 mAb, atezolizumab, increases their death, provided the absence of AR. In our experimental setting, the efficacy of anti PD-1 mAb, permbrolizumab, and anti CTLA-4 mAb, ipilimumab, is restricted to just one melanoma cell line. Although interesting, these findings raise many questions. Firstly, how might the ICIs act on melanoma cells in the absence of immune cell populations? Through engagement of PD-1-expressing tumor-infiltrating lymphocytes, PD-L1 promotes melanoma immune escape and progression [80]. As in our experimental setting PD-L1 and PD-1 are both expressed in melanoma cells, the possibility that they interact each other *via* paracrine or autocrine interactions [55] cannot be ruled out. Thus, specific interference in this interaction by anti-PD-L1 or anti PD-1 antibodies would induce cell death and block tumor growth. Concerning CTLA-4, it should be noticed that despite its prevalence in the T cell lineage, its expression in melanoma has been demonstrated [56]. However, the possibility that melanoma cells express the CTLA-4 ligand, CD86, cannot be excluded. Thus, CD86 might promote cell-cell contacts through the binding to CTLA-4 [81]. Notwithstanding the relevance of our findings, we are aware that many data are still required to deeply investigate the role of AR in immunity. Moreover, new approaches of AR-based immunocheckpoint blockade, which combine specific modulation of AR with immunocheckpoint inhibitors, can be envisaged. Studies in ‘*in vivo’* models, genome wide screening, identification of novel subsets of AR-positive immune cells, design and synthesis of new AR inhibitors/modulators with strong efficacy and tissue selectivity might provide new hints in this direction to achieve a better therapeutic efficacy in melanoma patients.

## Supplementary information

Supplemental material

Table 1S

Supplemental material: Full and uncropped WB

## Data Availability

All data generated and analyzed during this study are included in this published article and supplemental materials. Supplemental figures and full and uncropped western blots were shown in Supplemental materials. DNA sequences are deposited in unstructured repository (10.6084/m9.figshare.22550095.v1).

## References

[1] Siegel RL, Miller KD, Fuchs HE, Jemal A. Cancer statistics. CA Cancer J Clin. 2022;72:7–33. 35020204 10.3322/caac.21708

[2] Ascierto PA, Agarwala SS, Blank C, Caracò C, Carvajal RD, Ernstoff MS, et al. Perspectives in Melanoma: meeting report from the Melanoma Bridge (December 2nd – 4th, 2021, Italy). J Transl Med. 2022;20:391. 36058945 10.1186/s12967-022-03592-4 PMC9440864

[3] Larkin J, Chiarion-Sileni V, Gonzalez R, Grob JJ, Cowey CL, Lao CD, et al. Combined Nivolumab and Ipilimumab or Monotherapy in Untreated Melanoma. N Engl J Med. 2015;373:23–34. 26027431 10.1056/NEJMoa1504030 PMC5698905

[4] Gide TN, Wilmott JS, Scolyer RA, Long GV. Primary and Acquired Resistance to Immune Checkpoint Inhibitors in Metastatic Melanoma. Clin Cancer Res. 2018;24:1260–70. 29127120 10.1158/1078-0432.CCR-17-2267

[5] Erturk K, Tas F. Effect of biology on the outcome of female melanoma patients. Mol Clin Oncol. 2017;7:1093–100. 29285381 10.3892/mco.2017.1446 PMC5740849

[6] Crocetti E, Mallone S, Robsahm TE, Gavin A, Agius D, Ardanaz E, et al. Survival of patients with skin melanoma in Europe increases further: Results of the EUROCARE-5 study. Eur J Cancer. 2015;51:2179–90. 26421821 10.1016/j.ejca.2015.07.039

[7] de Vries E, Nijsten TE, Visser O, Bastiaannet E, van Hattem S, Janssen-Heijnen ML, et al. Superior survival of females among 10,538 Dutch melanoma patients is independent of Breslow thickness, histologic type and tumor site. Ann Oncol. 2018;19:583–9. 10.1093/annonc/mdm498 17974555

[8] Kemeny MM, Busch E, Stewart AK, Menck HR. Superior survival of young women with malignant melanoma. Am J Surg. 1998;175:437–45. 9645769 10.1016/s0002-9610(98)00070-1

[9] Lasithiotakis K, Leiter U, Meier F, Eigentler T, Metzler G, Moehrle M, et al. Age and gender are significant independent predictors of survival in primary cutaneous melanoma. Cancer. 2008;112:1795–804. 18306371 10.1002/cncr.23359

[10] Mervic L, Leiter U, Meier F, Eigentler T, Forschner A, Metzler G, et al. Sex differences in survival of cutaneous melanoma are age dependent: an analysis of 7338 patients. Melanoma Res. 2011;21:244–52. 21540649 10.1097/CMR.0b013e32834577c8

[11] Scoggins CR, Ross MI, Reintgen DS, Noyes RD, Goydos JS, Beitsch PD, et al. Gender-related differences in outcome for melanoma patients. Ann Surg. 2006;243:693–700. 16633005 10.1097/01.sla.0000216771.81362.6b PMC1570554

[12] Wang Y, Ou Z, Sun Y, Yeh S, Wang X, Long J, et al. Androgen receptor promotes melanoma metastasis via altering the miRNA-539-3p/USP13/MITF/AXL signals. Oncogene. 2017;36:1644–54. 27869170 10.1038/onc.2016.330

[13] Ma M, Ghosh S, Tavernari D, Katarkar A, Clocchiatti A, Mazzeo L, et al. Sustained androgen receptor signaling is a determinant of melanoma cell growth potential and tumorigenesis. J Exp Med. 2021;218:e20201137. 33112375 10.1084/jem.20201137 PMC7596884

[14] Liu Q, Adhikari E, Lester DK, Fang B, Johnson JO, Tian Y, et al. Androgen drives melanoma invasiveness and metastatic spread by inducing tumorigenic fucosylation. Nat Commun. 2024;15:1148. 38326303 10.1038/s41467-024-45324-w PMC10850104

[15] Vellano CP, White MG, Andrews MC, Chelvanambi M, Witt RG, Daniele JR, et al. Androgen receptor blockade promotes response to BRAF/MEK-targeted therapy. Nature. *2022*;606:797–803. 35705814 10.1038/s41586-022-04833-8 PMC10071594

[16] Marzagalli M, Ebelt ND, Manuel ER. Unraveling the crosstalk between melanoma and immune cells in the tumor microenvironment. Semin Cancer Biol. 2019;59:236–50. 31404607 10.1016/j.semcancer.2019.08.002

[17] Kissick HT, Sanda MG, Dunn LK, Pellegrini KL, On ST, Noel JK, et al. Androgens alter T-cell immunity by inhibiting T-helper 1 differentiation. Proc Natl Acad Sci USA. 2014;111:9887–92. 24958858 10.1073/pnas.1402468111 PMC4103356

[18] Gubbels Bupp MR, Jorgensen TN. Androgen-Induced Immunosuppression. Front Immunol. 2018;9:794. 29755457 10.3389/fimmu.2018.00794 PMC5932344

[19] Guan X, Polesso F, Wang C, Sehrawat A, Hawkins RM, Murray SE, et al. Androgen receptor activity in T cells limits checkpoint blockade efficacy. Nature. 2022;606:791–6. 35322234 10.1038/s41586-022-04522-6 PMC10294141

[20] Pala L, De Pas T, Conforti F. Boosting anticancer immunotherapy through androgen receptor blockade. Cancer Cell. 2022;40:455–7. 35537411 10.1016/j.ccell.2022.04.007

[21] Garofalo C, De Marco C, Cristiani CM. NK Cells in the Tumor Microenvironment as New Potential Players Mediating Chemotherapy Effects in Metastatic Melanoma. Front Oncol. 2021;11:754541. 34712615 10.3389/fonc.2021.754541 PMC8547654

[22] Cristiani CM, Turdo A, Ventura V, Apuzzo T, Capone M, Madonna G, et al. Accumulation of Circulating CCR7+ Natural Killer Cells Marks Melanoma Evolution and Reveals a CCL19-Dependent Metastatic Pathway. Cancer Immunol Res. 2019;7:841–52. 30940644 10.1158/2326-6066.CIR-18-0651

[23] Dobos J, Mohos A, Tóvári J, Rásó E, Lőrincz T, Zádori G, et al. Sex-dependent liver colonization of human melanoma in SCID mice-role of host defense mechanisms. Clin Exp Metastasis. 2013;30:497–506. 23203681 10.1007/s10585-012-9554-5

[24] Castoria G, Giovannelli P, Di Donato M, Ciociola A, Hayashi R, Bernal F, et al. Role of non-genomic androgen signalling in suppressing proliferation of fibroblasts and fibrosarcoma cells. Cell Death Dis. 2014;5:e1548. 25476896 10.1038/cddis.2014.497 PMC4649827

[25] Di Donato M, Zamagni A, Galasso G, Di Zazzo E, Giovannelli P, Barone MV, et al. The androgen receptor/filamin A complex as a target in prostate cancer microenvironment. Cell Death Dis. 2021;12:127. 33500395 10.1038/s41419-021-03402-7 PMC7838283

[26] Di Donato M, Cernera G, Migliaccio A, Castoria G. Nerve Growth Factor Induces Proliferation and Aggressiveness in Prostate Cancer Cells. Cancers. 2019;11:784. 31174415 10.3390/cancers11060784 PMC6627659

[27] Giovannelli P, Di Donato M, Auricchio F, Castoria G, Migliaccio A. Androgens Induce Invasiveness of Triple Negative Breast Cancer Cells Through AR/Src/PI3-K Complex Assembly. Sci Rep. 2019;9:4490. 30872694 10.1038/s41598-019-41016-4 PMC6418124

[28] Pesce S, Thoren FB, Cantoni C, Prato C, Moretta L, Moretta A, et al. The Innate Immune Cross Talk between NK Cells and Eosinophils Is Regulated by the Interaction of Natural Cytotoxicity Receptors with Eosinophil Surface Ligands. Front Immunol. 2017;28:510. 10.3389/fimmu.2017.00510 PMC5408020 28503177

[29] McGinnes K, Chapman G, Penny R. Effects of interferon on natural killer (NK) cells assessed by fluorescent probes and flow cytometry. J Immunol Methods. 1998;107:129–36. 10.1016/0022-1759(88)90018-x 2449502

[30] Tsubuki S, Saito Y, Tomioka M, Ito H, Kawashima S. Differential inhibition of calpain and proteasome activities by peptidyl aldehydes of di-leucine and tri-leucine. J Biochem. 1996;119:572–6. 8830056 10.1093/oxfordjournals.jbchem.a021280

[31] Paschen A, Sucker A, Hill B, Moll I, Zapatka M, Nguyen XD, et al. Differential clinical significance of individual NKG2D ligands in melanoma: soluble ULBP2 as an indicator of poor prognosis superior to S100B. Clin Cancer Res. 2009;15:5208–15. 19671853 10.1158/1078-0432.CCR-09-0886

[32] Myers JA, Miller JS. Exploring the NK cell platform for cancer immunotherapy. Nat Rev Clin Oncol. 2021;18:85–100. 32934330 10.1038/s41571-020-0426-7 PMC8316981

[33] Pardoll DM. Distinct mechanisms of tumor resistance to NK killing: of mice and men. Immunity. 2015;42:605–6. 25902479 10.1016/j.immuni.2015.04.007 PMC5598348

[34] Bald T, Krummel MF, Smyth MJ, Barry KC. The NK cell-cancer cycle: advances and new challenges in NK cell-based immunotherapies. Nat Immunol. 2020;21:835–47. 32690952 10.1038/s41590-020-0728-z PMC8406687

[35] Koguchi Y, Hoen HM, Bambina SA, Rynning MD, Fuerstenberg RK, Curti BD, et al. Serum Immunoregulatory Proteins as Predictors of Overall Survival of Metastatic Melanoma Patients Treated with Ipilimumab. Cancer Res. 2015;75:5084–92. 26627641 10.1158/0008-5472.CAN-15-2303

[36] Waldhauer I, Goehlsdorf D, Gieseke F, Weinschenk T, Wittenbrink M, Ludwig A, et al. Tumor-associated MICA is shed by ADAM proteases. Cancer Res. 2008;68:6368–76. 18676862 10.1158/0008-5472.CAN-07-6768

[37] Wang X, Lundgren AD, Singh P, Goodlett DR, Plymate SR, Wu JD. An six-amino acid motif in the alpha3 domain of MICA is the cancer therapeutic target to inhibit shedding. Biochem Biophys Res Commun. 2009;387:476–81. 19615970 10.1016/j.bbrc.2009.07.062 PMC2737406

[38] Liu G, Atteridge CL, Wang X, Lundgren AD, Wu JD. The membrane type matrix metalloproteinase MMP14 mediates constitutive shedding of MHC class I chain-related molecule A independent of A disintegrin and metalloproteinases. J Immunol. 2010;184:3346–50. 20208009 10.4049/jimmunol.0903789 PMC3191873

[39] Blobel CP. Metalloprotease-Disintegrins: Links to Cell Adhesion and Cleavage of TNFα and Notch. Cell. 1997;90:589–92. 9288739 10.1016/s0092-8674(00)80519-x

[40] Bridges LC, Bowditch RD. ADAM-Integrin Interactions: potential integrin regulated ectodomain shedding activity. Curr Pharm Des. 2005;11:837–47. 15777238 10.2174/1381612053381747

[41] Zhang XP, Kamata T, Yokoyama K, Puzon-McLaughlin W, Takada Y. Specific interaction of the recombinant disintegrin-like domain of MDC-15 (metargidin, ADAM-15) with integrin alphavbeta3. J Biol Chem. 1998;273:7345–50. 9516430 10.1074/jbc.273.13.7345

[42] Eto K, Puzon-McLaughlin W, Sheppard D, Sehara-Fujisawa A, Zhang XP, Takada Y. RGD-independent binding of integrin alpha9beta1 to the ADAM-12 and -15 disintegrin domains mediates cell-cell interaction. J Biol Chem. 2000;275:34922–30. 10944520 10.1074/jbc.M001953200

[43] Krätzschmar J, Lum L, Blobel CP. Metargidin, a membrane-anchored metalloprotease-disintegrin protein with an RGD integrin binding sequence. J Biol Chem. 1996;271:4593–6. 8617717 10.1074/jbc.271.9.4593

[44] Castoria G, D’Amato L, Ciociola A, Giovannelli P, Giraldi T, Sepe L, et al. Androgen-induced cell migration: role of androgen receptor/filamin A association. PLoS One. 2011;6:e17218. 21359179 10.1371/journal.pone.0017218 PMC3040221

[45] Di Donato M, Bilancio A, D’Amato L, Claudiani P, Oliviero MA, Barone MV, et al. Cross-talk between androgen receptor/filamin A and TrkA regulates neurite outgrowth in PC12 cells. Mol Biol Cell. 2015;26:2858–72. 26063730 10.1091/mbc.E14-09-1352 PMC4571344

[46] Ludwig A, Hundhausen C, Lambert MH, Broadway N, Andrews RC, Bickett DM, et al. Metalloproteinase inhibitors for the disintegrin-like metalloproteinases ADAM10 and ADAM17 that differentially block constitutive and phorbol ester-inducible shedding of cell surface molecules. Comb Chem High Throughput Screen. 2005;8:161–71. 15777180 10.2174/1386207053258488

[47] Du W, Nair P, Johnston A, Wu PH, Wirtz D. Cell Trafficking at the Intersection of the Tumor-Immune Compartments. Annu Rev Biomed Eng. 2022;24:275–305. 35385679 10.1146/annurev-bioeng-110320-110749 PMC9811395

[48] Edwards DR, Handsley MM, Pennington CJ. The ADAM metalloproteinases. Mol Aspects Med. 2008;29:258–89. 18762209 10.1016/j.mam.2008.08.001 PMC7112278

[49] Kang Y, Hu W, Ivan C, Dalton HJ, Miyake T, Pecot CV, et al. Role of focal adhesion kinase in regulating YB-1-mediated paclitaxel resistance in ovarian cancer. J Natl Cancer Inst. 2013;105:14851–495. 10.1093/jnci/djt210 PMC3787907 24062525

[50] Dudley DT, Pang L, Decker SJ, Bridges AJ, Saltiel AR. A synthetic inhibitor of the mitogen-activated protein kinase cascade. Proc Natl Acad Sci USA. 1995;92:768. 7644477 10.1073/pnas.92.17.7686 PMC41210

[51] Zhao L, Hu H, Gustafsson JÅ, Zhou S. Nuclear Receptors in Cancer Inflammation and Immunity. Trends Immunol. 2020;41:172–85. 31982345 10.1016/j.it.2019.12.006

[52] Cristiani CM, Garofalo C, Passacatini LC, Carbone E. New avenues for melanoma immunotherapy: Natural Killer cells? Scand J Immunol. 2020;91:e12861. 31879979 10.1111/sji.12861

[53] Sunshine JC, Nguyen PL, Kaunitz GJ, Cottrell TR, Berry S, Esandrio J, et al. PD-L1 Expression in Melanoma: A Quantitative Immunohistochemical Antibody Comparison. Clin Cancer Res. 2017;23:4938–44. 28428193 10.1158/1078-0432.CCR-16-1821 PMC6175606

[54] Schatton T, Schütte U, Frank NY, Zhan Q, Hoerning A, Robles SC, et al. Modulation of T-cell activation by malignant melanoma initiating cells. Cancer Res. 2010;70:697–708. 20068175 10.1158/0008-5472.CAN-09-1592 PMC2883769

[55] Kleffel S, Posch C, Barthel SR, Mueller H, Schlapbach C, Guenova E, et al. Melanoma Cell-Intrinsic PD-1 Receptor Functions Promote Tumor Growth. Cell. 2015;162:242–56. 10.1016/j.cell.2015.08.052 PMC4700833 26359984

[56] Laurent S, Queirolo P, Boero S, Salvi S, Piccioli P, Boccardo S, et al. The engagement of CTLA-4 on primary melanoma cell lines induces antibody-dependent cellular cytotoxicity and TNF-alpha production. J Transl Med. 2013;11:108. 23634660 10.1186/1479-5876-11-108 PMC3663700

[57] Pistillo MP, Carosio R, Grillo F, Fontana V, Mastracci L, Morabito A, et al. Phenotypic characterization of tumor CTLA-4 expression in melanoma tissues and its possible role in clinical response to Ipilimumab. Clin Immunol. 2020;215:108428. 32344017 10.1016/j.clim.2020.108428

[58] Kumar P, Nagarajan A, Uchil PD. Analysis of Cell Viability by the Lactate Dehydrogenase Assay. Cold Spring Harb Protoc. 2018; 10.1101/pdb.prot095497. 10.1101/pdb.prot095497 29858337

[59] Gupta S, Artomov M, Goggins W, Daly M, Tsao H. Gender Disparity and Mutation Burden in Metastatic Melanoma. J Natl Cancer Inst. 2015;107:djv221. 26296643 10.1093/jnci/djv221 PMC4643631

[60] Joosse A, de Vries E, Eckel R, Nijsten T, Eggermont AM, Hölzel D, et al. Gender differences in melanoma survival: female patients have a decreased risk of metastasis. J Invest Dermatol. 2011;131:719–26. 21150923 10.1038/jid.2010.354

[61] Robinson JK, Mallett KA, Turrisi R, Stapleton J. Engaging Patients and Their Partners in Preventive Health Behaviors. Arch Dermatol. 2009;145:469–73. 19380671 10.1001/archdermatol.2009.2 PMC2907148

[62] Swetter SM, Johnson TM, Miller DR, Layton CJ, Brooks KR, Geller AC. Melanoma in middle-aged and older men: a multi-institutional survey study of factors related to tumor thickness. Arch Dermatol. 2009;145:397–404. 19380661 10.1001/archdermatol.2008.603

[63] Holdenrieder S, Stieber P, Peterfi A, Nagel D, Steinle A, Salih HR. Soluble MICA in malignant diseases. Int J Cancer. 2006;118:684–7. 16094621 10.1002/ijc.21382

[64] Holdenrieder S, Stieber P, Peterfi A, Nagel D, Steinle A, Salih HR. Soluble MICB in malignant diseases: analysis of diagnostic significance and correlation with soluble MICA. Cancer Immunol Immunother. 2006;55:1584–9. 16636811 10.1007/s00262-006-0167-1 PMC11030555

[65] Wu JD, Higgins LM, Steinle A, Cosman D, Haugk K, Plymate SR. Prevalent expression of the immunostimulatory MHC class I chain-related molecule is counteracted by shedding in prostate cancer. J Clin Invest. 2004;114:560–8. 15314693 10.1172/JCI22206 PMC503776

[66] Jinushi M, Takehara T, Tatsumi T, Hiramatsu N, Sakamori R, Yamaguchi S, et al. Impairment of natural killer cell and dendritic cell functions by the soluble form of MHC class I-related chain A in advanced human hepatocellular carcinomas. J Hepatol. 2005;43:1013–20. 16168521 10.1016/j.jhep.2005.05.026

[67] Salih HR, Rammensee H-G, Steinle A. Cutting edge: down-regulation of MICA on human tumors by proteolytic shedding. J Immunol. 2002;169:4098–102. 12370336 10.4049/jimmunol.169.8.4098

[68] Salih HR, Goehlsdorf D, Steinle A. Release of MICB molecules by tumor cells: mechanism and soluble MICB in sera of cancer patients. Hum Immunol. 2006;67:188–95. 16698441 10.1016/j.humimm.2006.02.008

[69] Waldhauer I, Steinle A. Proteol ytic release of soluble UL16-binding protein 2 from tumor cells. Cancer Res. 2006;66:2520–6. 16510567 10.1158/0008-5472.CAN-05-2520

[70] McCulloch DR, Harvey M, Herington AC. The expression of the ADAMs proteases in prostate cancer cell lines and their regulation by dihydrotestosterone. Mol Cell Endocrinol. 2000;167:11–21. 11000516 10.1016/s0303-7207(00)00305-1

[71] Sorrentino C, D’Angiolo R, Gentile G, Giovannelli P, Perillo B, Migliaccio A, et al. The Androgen Regulation of Matrix Metalloproteases in Prostate Cancer and Its Related Tumor Microenvironment. Endocrines. 2023;4:350–65.

[72] Conforti F, Pala L, Bagnardi V, De Pas T, Martinetti M, Viale G, et al. Cancer immunotherapy efficacy and patients’ sex: a systematic review and meta-analysis. Lancet Oncol. 2018;19:737–46. 29778737 10.1016/S1470-2045(18)30261-4

[73] Litchfield K, Reading JL, Puttick C, Thakkar K, Abbosh C, Bentham R, et al. Meta-analysis of tumor- and T cell-intrinsic mechanisms of sensitization to checkpoint inhibition. Cell. 2021;184:596–614.e14. 33508232 10.1016/j.cell.2021.01.002 PMC7933824

[74] Natale CA, Li J, Zhang J, Dahal A, Dentchev T, Stanger BZ, et al. Activation of G protein-coupled estrogen receptor signaling inhibits melanoma and improves response to immune checkpoint blockade. Elife. 2018;7:e31770. 29336307 10.7554/eLife.31770 PMC5770157

[75] Smalley KS. Why do women with melanoma do better than men? Elife. 2018;7:e33511. 29336304 10.7554/eLife.33511 PMC5770156

[76] Pinto JA, Vallejos CS, Raez LE, Mas LA, Ruiz R, Torres-Roman JS, et al. Gender and outcomes in non-small cell lung cancer: an old prognostic variable comes back for targeted therapy and immunotherapy? ESMO Open. 2018;3:e000344. 29682332 10.1136/esmoopen-2018-000344 PMC5905840

[77] Capone I, Marchetti P, Ascierto PA, Malorni W, Gabriele L. Sexual Dimorphism of Immune Responses: A New Perspective in Cancer Immunotherapy. Front Immunol. 2018;9:552. 29619026 10.3389/fimmu.2018.00552 PMC5871673

[78] Péqueux C, Raymond-Letron I, Blacher S, Boudou F, Adlanmerini M, Fouque MJ, et al. Stromal estrogen receptor-α promotes tumor growth by normalizing an increased angiogenesis. Cancer Res. 2012;72:3010–9. 22523036 10.1158/0008-5472.CAN-11-3768

[79] Zhao L, Huang S, Mei S, Yang Z, Xu L, Zhou N. Y et al. Pharmacological activation of estrogen receptor beta augments innate immunity to suppress cancer metastasis. Proc Natl Acad Sci USA. 2018;115:E3673–E3681. 29592953 10.1073/pnas.1803291115 PMC5910874

[80] Dong H, Strome SE, Salomao DR, Tamura H, Hirano F, Flies DB, et al. Tumor-associated B7-H1 promotes T-cell apoptosis: a potential mechanism of immune evasion. Nat Med. 2002;8:793–800. 12091876 10.1038/nm730

[81] Contardi E, Palmisano GL, Tazzari PL, Martelli AM, Falà F, Fabbi M, et al. CTLA-4 is constitutively expressed on tumor cells and can trigger apoptosis upon ligand interaction. Int J Cancer. 2005;117:538–50. 15912538 10.1002/ijc.21155

