# Supplementary material for: Role of the androgen receptor in melanoma aggressiveness
Source: Cell Death Dis. 2025 Jan 21;16(1):34. doi: 10.1038/s41419-025-07350-4 (PMC11751086; doi:10.1038/s41419-025-07350-4)
Supplement: Supplementary file 1 — Supplemental material [file 41419_2025_7350_MOESM1_ESM.docx]

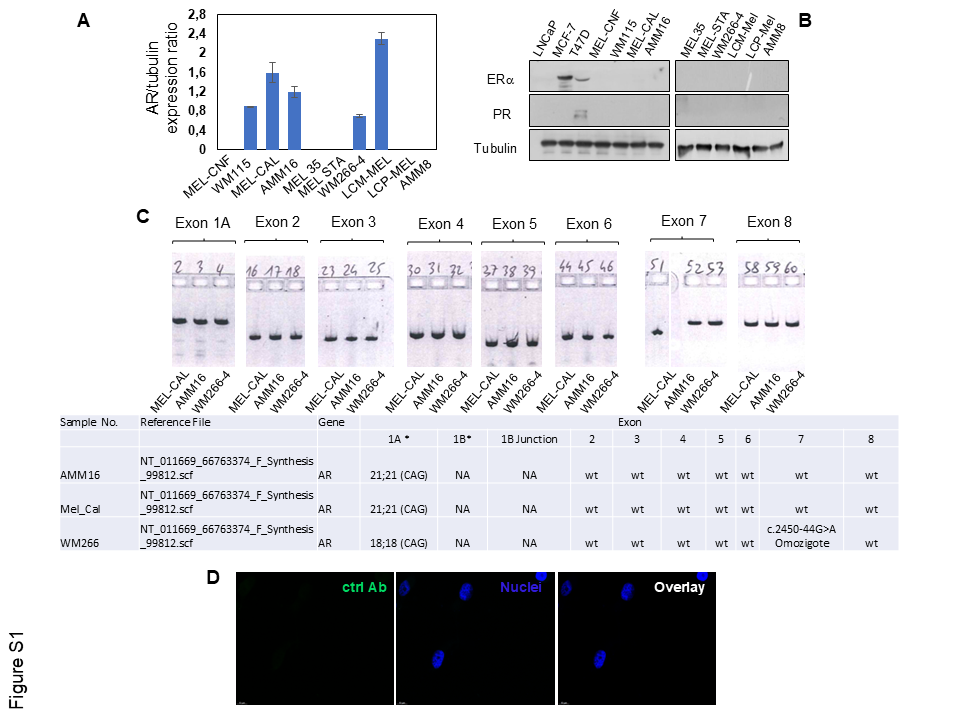


# Figure S1

1. AR protein expression levels normalized over the corresponding tubulin amounts and calculated by NIH Image J software. Means and SDs from three different experiments are shown.
2. WB analysis for ERα and PgR in the indicated cell lines. Tubulin was revealed as a loading control. LNCaP, T47D and MCF-7 cells are used as negative or positive controls.
3. PCR products obtained using the primer’s set indicated in Methods were amplified from MEL- CAL, AMM16 and WM266-4 genomic DNA. A PCR product for each exon corresponding to the size expected from a wild-type sequence was amplified from all genomic DNA samples and analyzed by agarose gel electrophoresis. The Table in the bottom represents the PCR report and the different amplified exons.
4. Untreated AMM16 cells on coverslips were stained with the secondary antibody (ctrl Ab) alone, as negative control. Nuclei were stained with Hoechst. Different fields were visualized by confocal microscopy analysis and representative images from three independent experiments were captured. *Bar*, 10 µm.


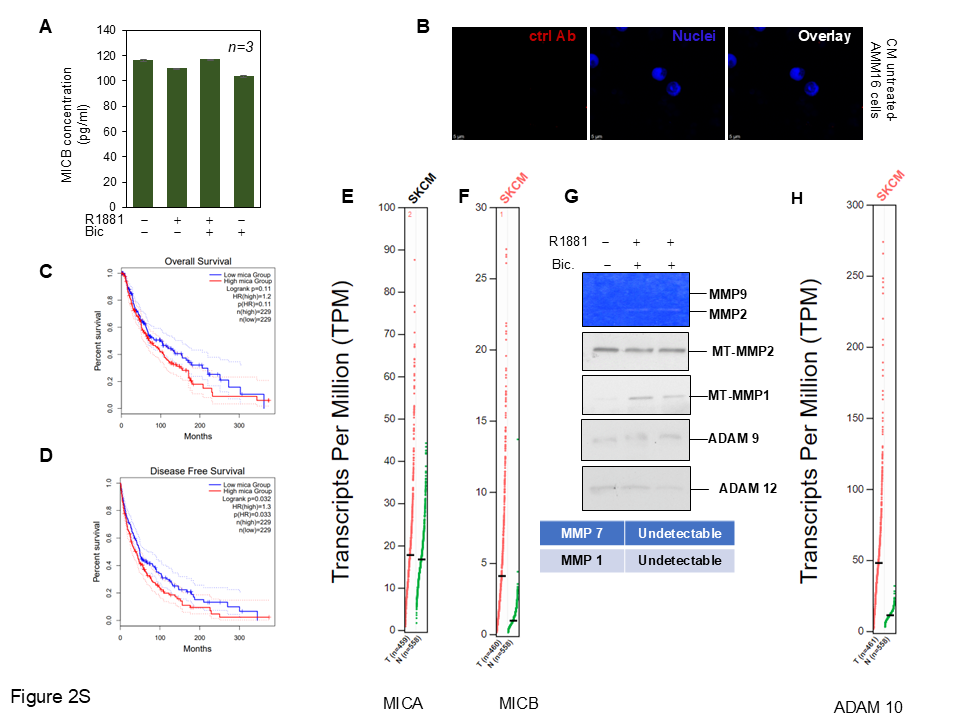


# Figure S2

1. AMM16 cells were unstimulated or stimulated for 6 h with R1881, in the absence or presence of the indicated compounds. sMICB was quantified by ELISA. Data from three (*n*=3) different experiments are expressed as mean ± SD. **(B)** NK cells were challenged for 18h with CM of untreated AMM16 cells and stained with the secondary antibody (ctrl Ab) alone, as negative control. Nuclei were stained with Hoechst. Different fields were visualized by confocal microscopy analysis and representative images from three independent experiments were captured. Bar, 5 µm. (**C** and **D**) High MICA expression levels (red) negatively correlate with overall survival (OS; **C**) and the disease-free survival (**D**) in melanoma patients. Analysis of MICA (**E**) and MICB (**F**) TPM in melanoma samples (n=459 for MICA and n=460 for MICB) *vs* their normal counterparts (n=558). (**G**) Cells were unstimulated or stimulated with R1881, in the absence or presence of Bicalutamide (Bic) for 6 h. The release of MMP-2 in conditioned media was analyzed by zymography, as described in Methods. MMP-9 release was undetectable. Lysate proteins were analyzed for MT-MMP2, MT-MMP1, ADAM 9, ADAM 12, MMP 7 and MMP 1 expression using specific antibodies. (**H**) Analysis of ADAM 10 TPM in melanoma samples (n=461) *vs* their normal counterparts (n=558).


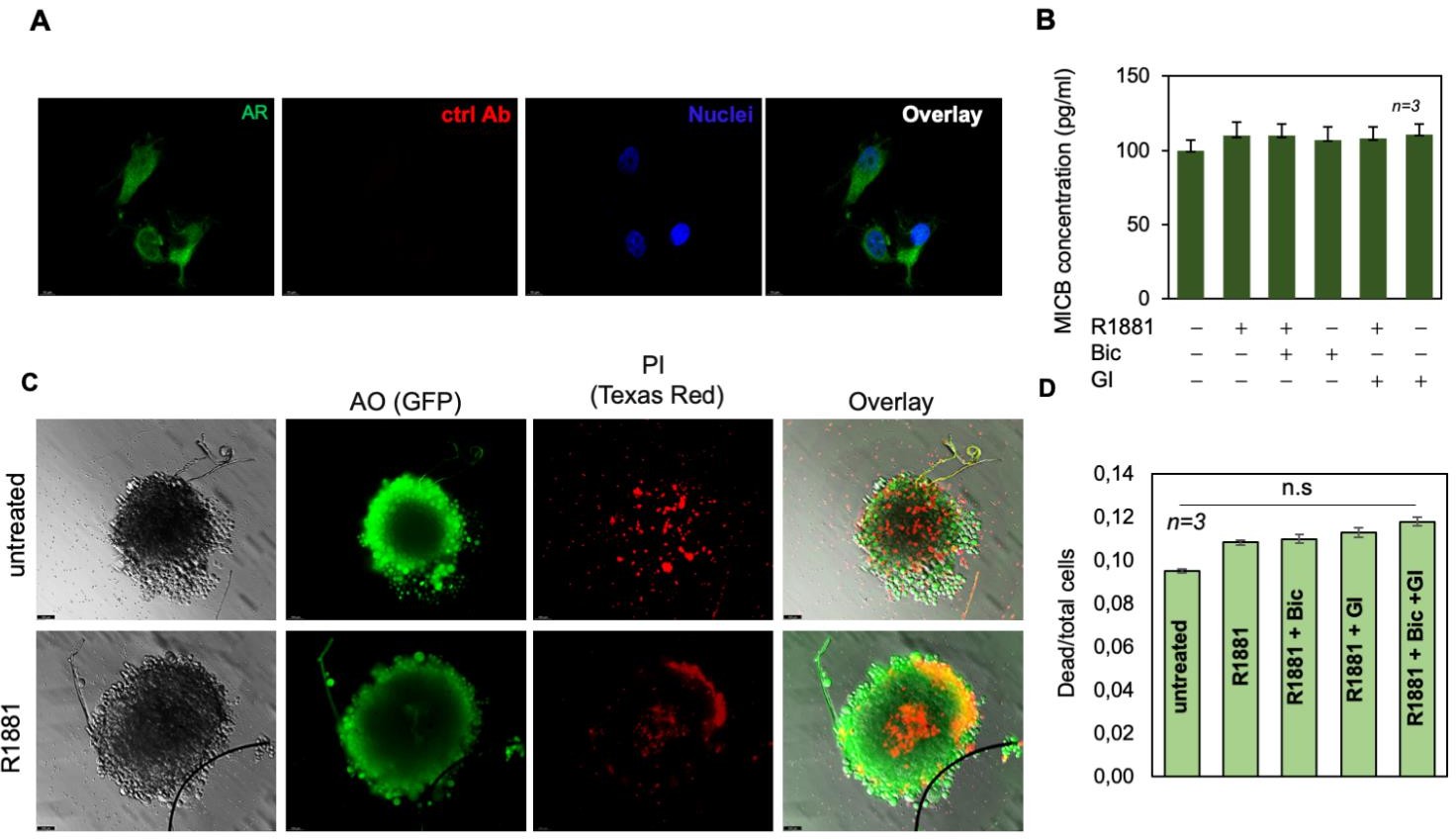


# Figure S3

(**A**) R1881-challenged (20 min) AMM16 cells were stained with the FITC-conjugated anti AR antibody in combination with the Texas-red conjugated secondary antibody (ctrl Ab), as negative control. Cells from three different experiments were analyzed by confocal microscopy and representative images, were captured and shown as control. *Bar*, 10 µm. (**B**) AMM16 cells were unstimulated or stimulated for 6 h with R1881, in the absence or presence of the indicated compounds. sMICB was quantified by ELISA. Data from three (n=3) different experiments are expressed as mean ± SD. (**C**) It shows representative images of AMM16-derived spheroids, untreated or treated as indicated in Figure, stained and co-coltured with NK cells for 10 minutes. The green fluorescence from the acridin orange staining indicates the total cells, while the red fluorescence from the propidium iodide staining indicates the dead cells. Bar, 100 µm. Images are representative of three different experiments. (**D**) The graph represents the dead cells/total cells. Values of dead and total cells were analyzed using NIH Image J software. They derive from red fluorescence mean/green fluorescence mean intensity and are expressed as mean ± SD of 3 different experiments (n=3).


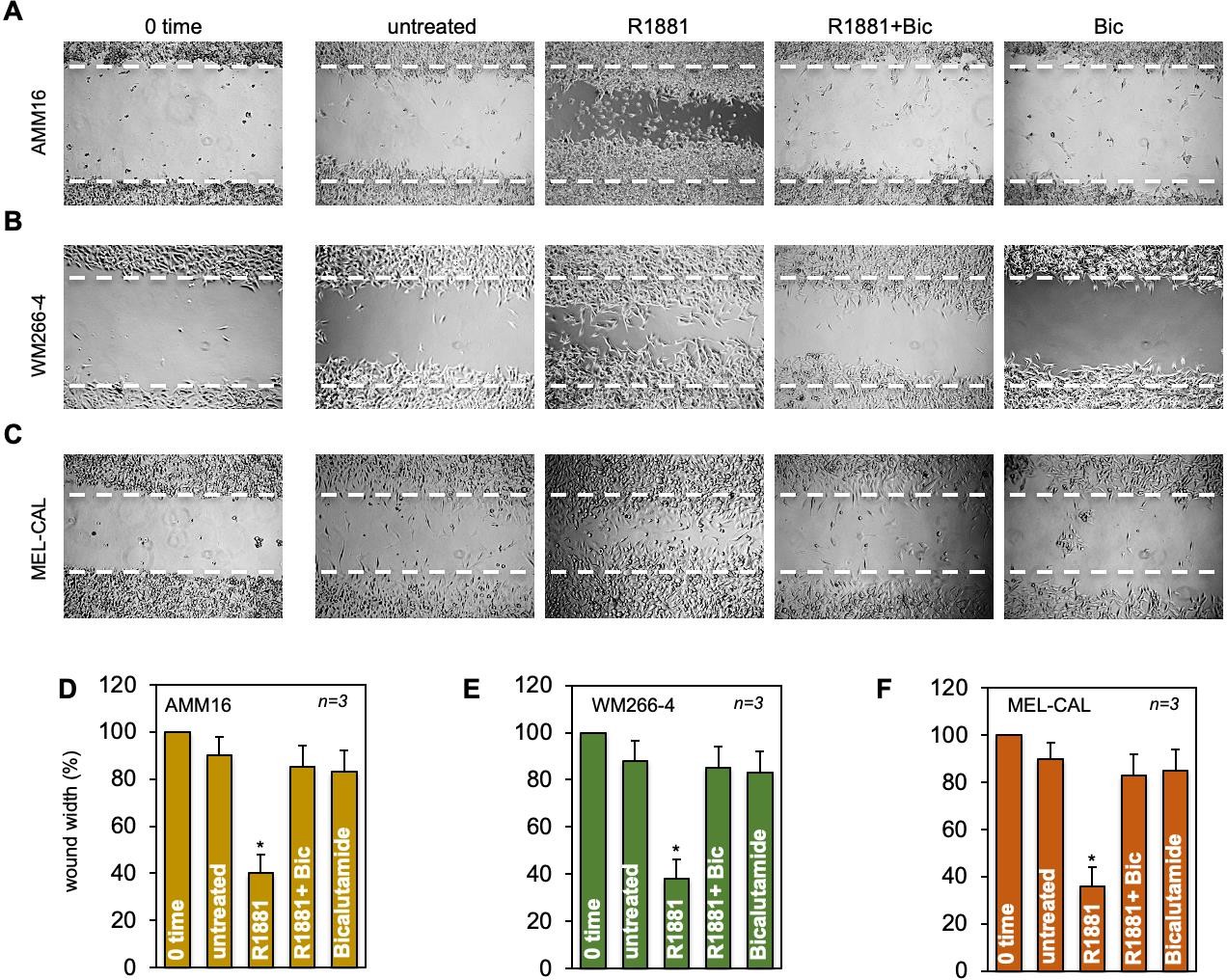


# Figure S4

AMM16 (**A**), WM266-4 (**B**) and MEL-CAL (**C**) cells were wounded and left untreated or treated with R1881 for 14 hours. Bicalutamide (Bic) was added at 10 μM. Phase-contrast images are representative of three different experiments, each in triplicate. In (**D-F**), the wound area was measured using Leica Suite Software and data are presented as % in wound width over the control cells, analyzed at 0 time. Means and SDs are shown. *n*, represents the number of experiments. * p

< 0.05 for the indicated experimental points *vs* the corresponding untreated control.


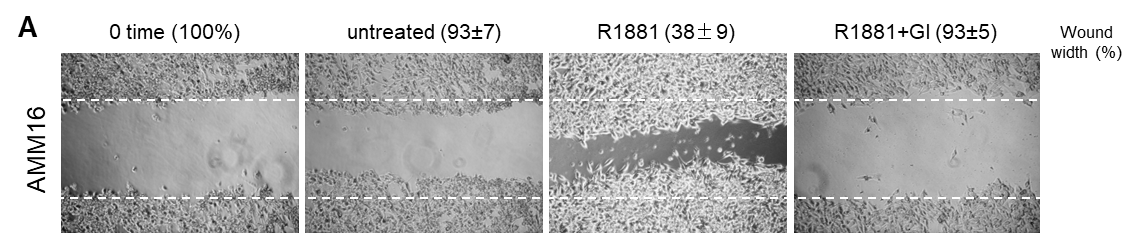


**Figure S5**

AMM16 (**A**) cells were wounded and left untreated or treated with R1881 for 14 hours. GI254023X (GI) was added at 10 μM. Phase-contrast images are representative of three different experiments, each in triplicate. The wound area was measured using Leica Suite Software and data are presented as % in wound width over the control cells, analyzed at 0 time. Means and SDs are shown.
