# Supplementary material for: Role of the androgen receptor in melanoma aggressiveness
Source: Cell Death Dis. 2025 Jan 21;16(1):34. doi: 10.1038/s41419-025-07350-4 (PMC11751086; doi:10.1038/s41419-025-07350-4)
Supplement: Supplementary file 2 — Table 1S [file 41419_2025_7350_MOESM2_ESM.docx]

|  | | UNTR | R1881 | p-value |
| --- | --- | --- | --- | --- |
| MHC-I | Frequency | 94.3 ± 6.8 | 96.8 ± 2.2 | 0.52 |
|  | Expression | 963 ± 224 | 975 ± 367 | 0.91 |
| PD-L1 | Frequency | 36.7 ± 6.9 | 42.0 ± 3.8 | 0.19 |
|  | Expression | 515 ± 14 | 516 ± 5 | 0.90 |
| Galectin-9 | Frequency | 1.60 ± 0.95 | 1.50 ± 0.87 | 0.42 |
|  | Expression | 529 ± 47 | 608 ± 155 | 0.38 |
| ULBP2 | Frequency | 9.9 ± 4.2 | 10.0 ± 4.2 | 0.95 |
|  | Expression | 539 ± 44 | 535 ± 39 | 0.29 |
| PVR | Frequency | 99.9 ± 0.1 | 99.9 ± 0.1 | 0.42 |
|  | Expression | 12580 ± 1703 | 13194 ± 1546 | 0.07 |
| Nectin-2 | Frequency | 92.2 ± 1.5 | 93.4 ± 2.3 | 0.27 |
|  | Expression | 763 ± 68 | 779 ± 31 | 0.54 |
| NKp30-L | Frequency | 3.9 ± 2.9 | 2.1 ± 1.5 | 0.74 |
|  | Expression | 430 ± 138 | 436 ± 117 | 0.81 |
| NKp44-L | Frequency | 5.4 ± 1.9 | 6.7 ± 3.0 | 0.61 |
|  | Expression | 1023 ± 425 | 1039 ± 557 | 0.94 |
| NKp46-L | Frequency | 4.7 ± 1.7 | 4.7 ± 2.5 | 0.99 |
|  | Expression | 647 ± 179 | 678 ± 181 | 0.75 |
| HLA-E | Frequency | 6.3 ± 4.3 | 4.4 ± 3.0 | 0.34 |
|  | Expression | 1557 ± 587 | 1279 ± 223 | 0.32 |
| ICAM-I | Frequency | 97.8 ± 0.8 | 98.1 ± 0.5 | 0.42 |
|  | Expression | 8245 ± 652 | 9269 ± 702 | 0.07 |
| E-cadherin | Frequency | 1.6 ± 0.6 | 1.4 ± 0.1 | 0.58 |
|  | Expression | 553 ± 29 | 552 ± 42 | 0.97 |

**Table 1S**
