# Supplementary material for: Role of the androgen receptor in melanoma aggressiveness
Source: Cell Death Dis. 2025 Jan 21;16(1):34. doi: 10.1038/s41419-025-07350-4 (PMC11751086; doi:10.1038/s41419-025-07350-4)
Supplement: Supplementary file 3 — Supplemental material: Full and uncropped WB [file 41419_2025_7350_MOESM3_ESM.pdf]

full and uncropped original western blots

Supplemental Material

Figure 1, panel b

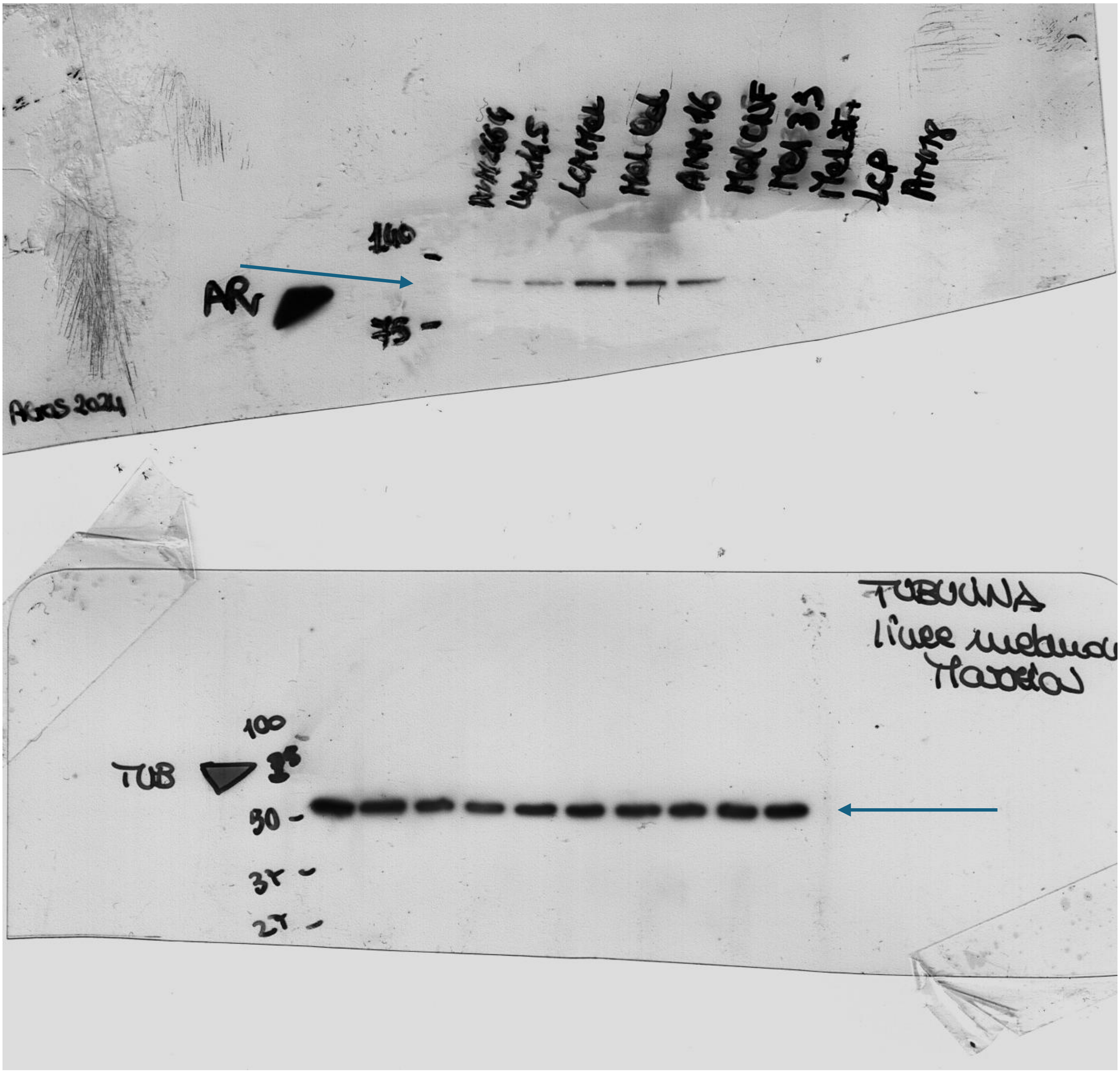

FIGURE 2
panel C

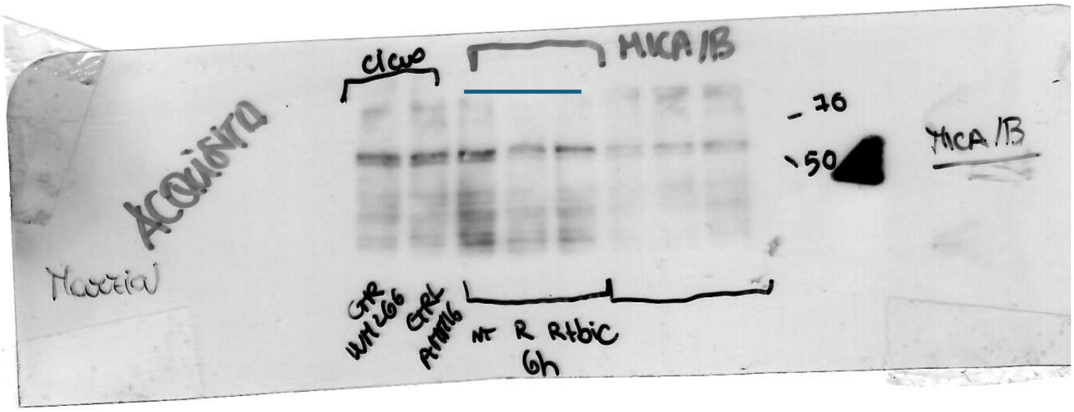

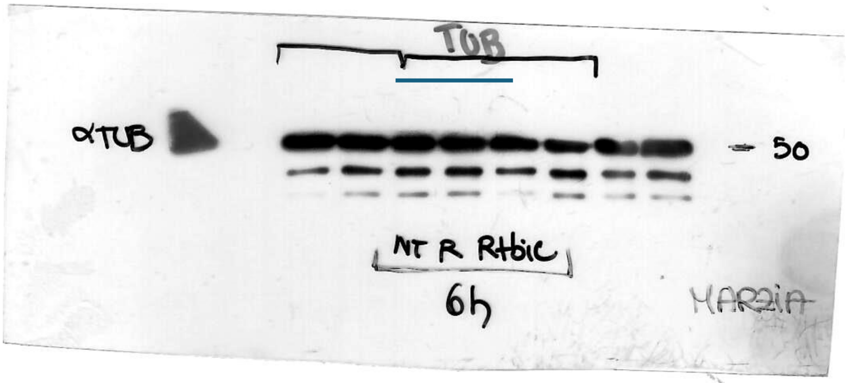

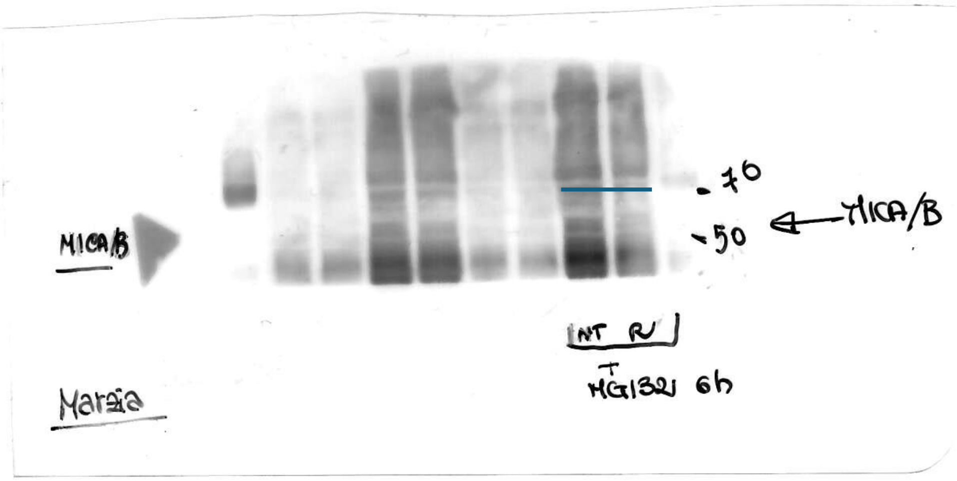

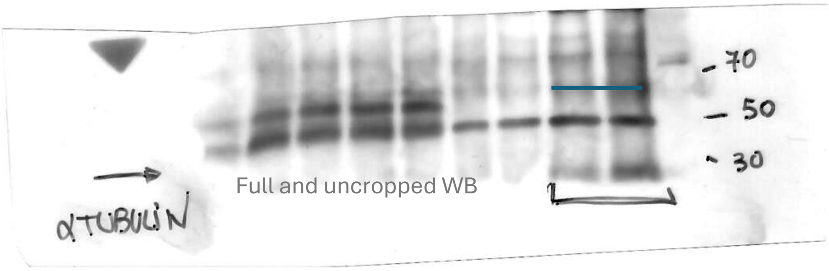

Figure 2F

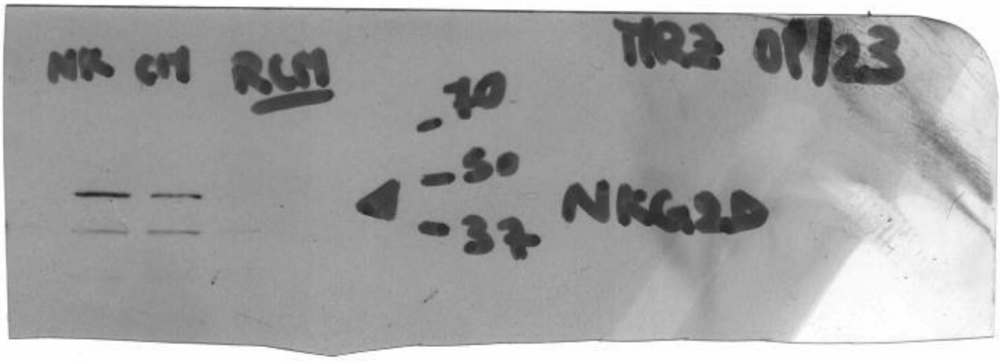
NK CM RCM
TIR2 09/23
70
50
37
NKG2D

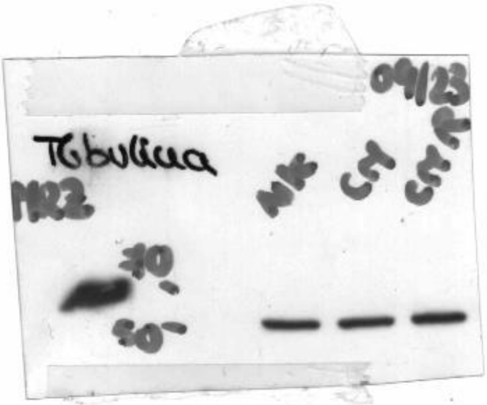
09/23
Tubulina
TIR2
NK CM CM R
70
50

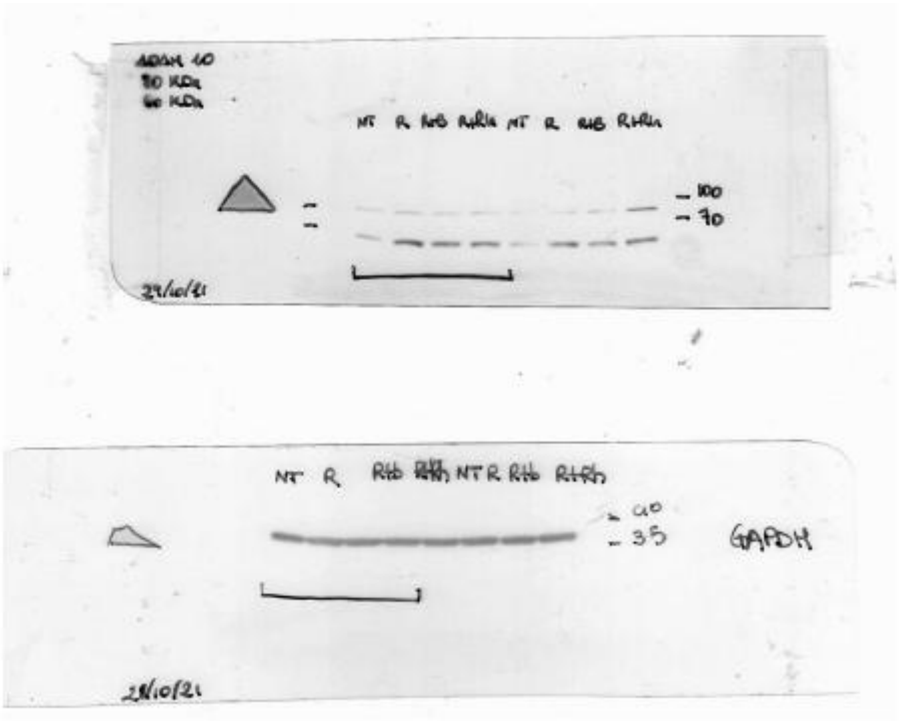

Fig. 3D

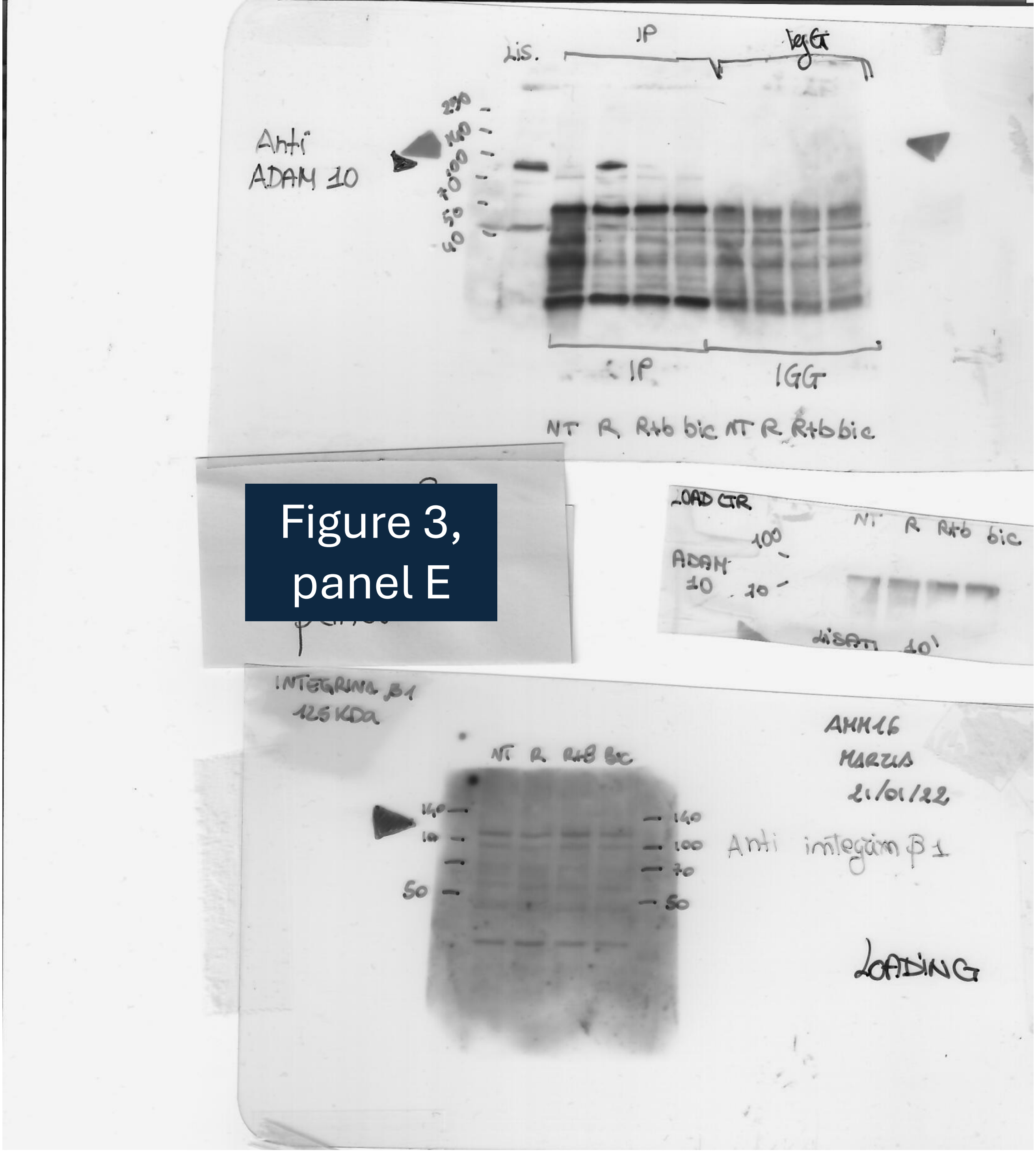
Lis.
IP
IgG
Anti
ADAM 10
IP
IGG
NT R R+b bic NT R R+b bic
Figure 3,
panel E
LOAD CTR
NT R R+b bic
ADAM
10
INTEGRINA β1
125 KDa
NT R R+B Bic
AMM16
MARZIA
21/01/22
Anti integrin β1
LOADING

Figure 3, panel E

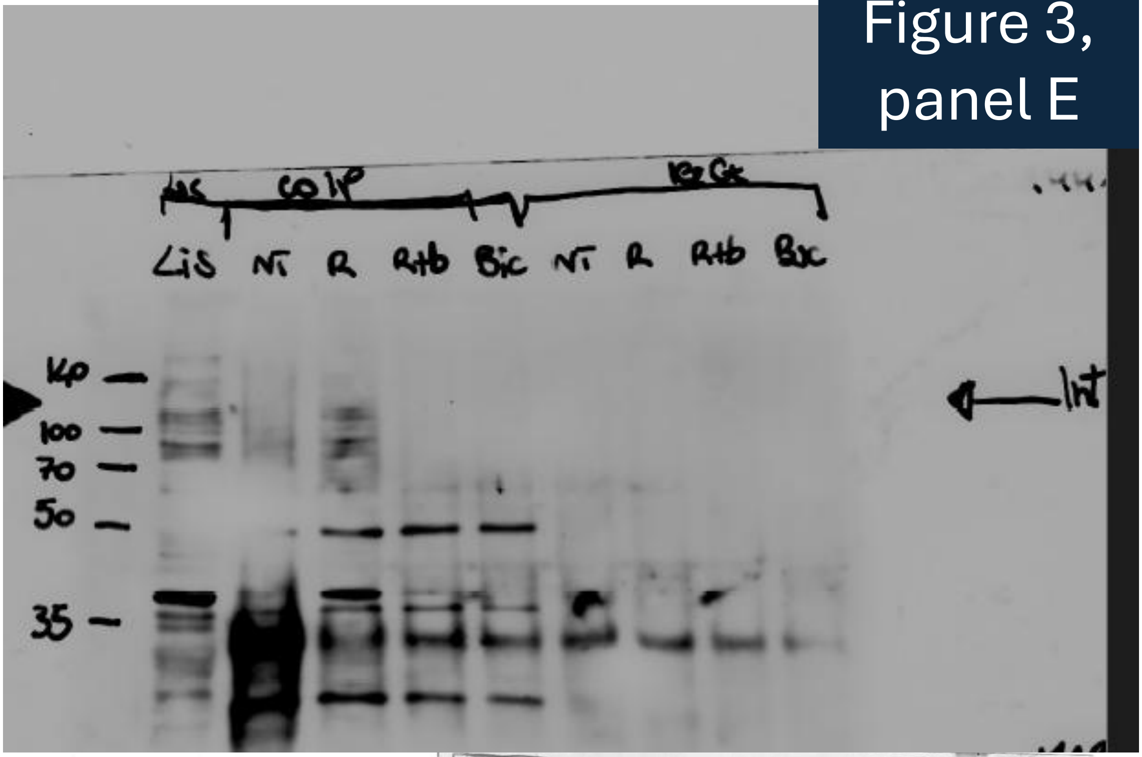

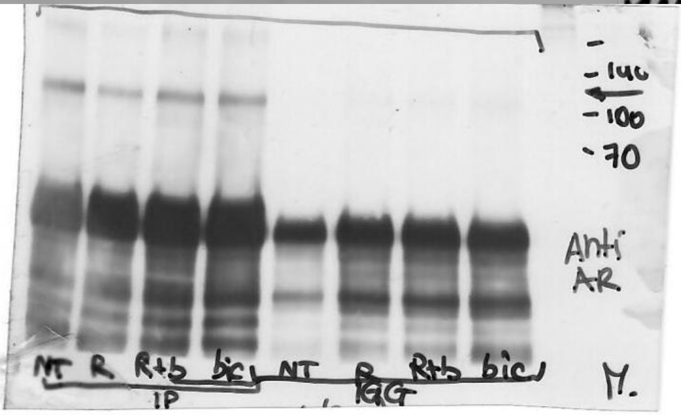

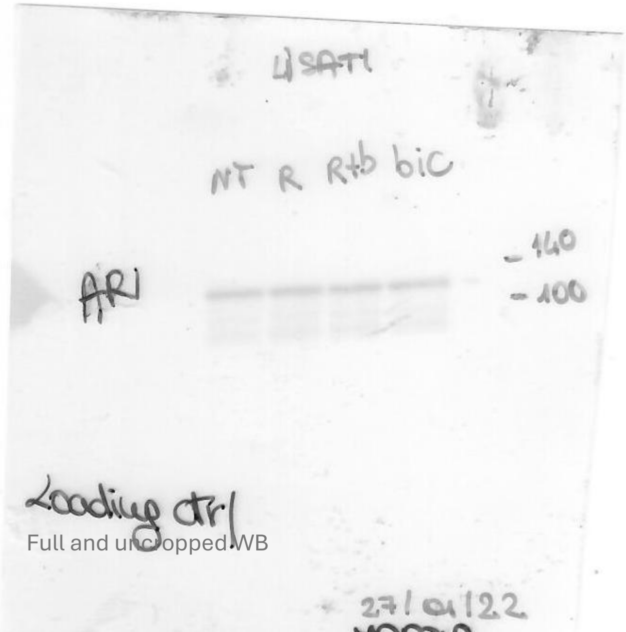

Figure 4A

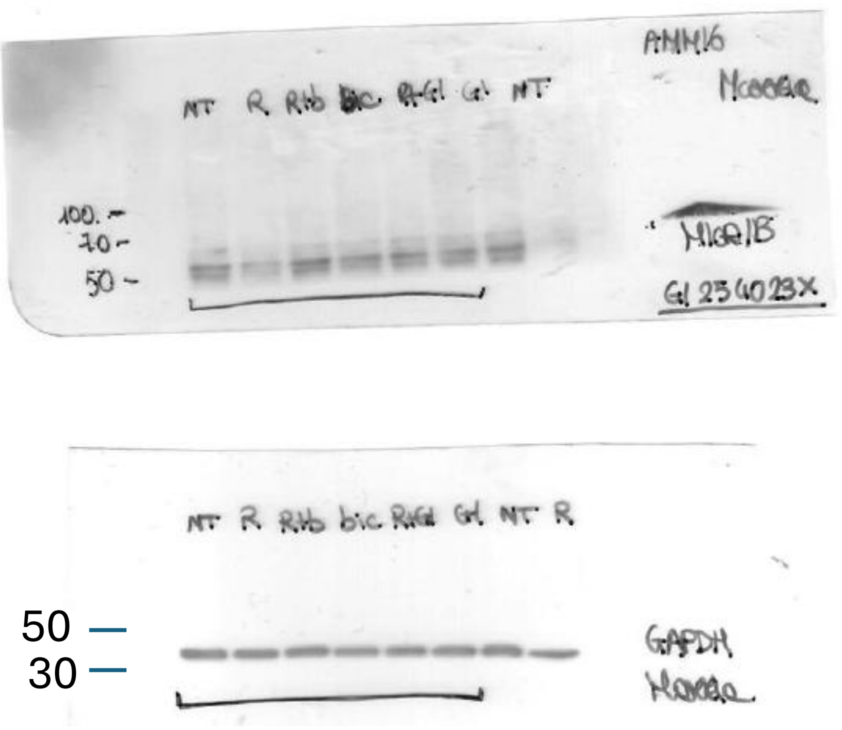

Figure 6 (M, N, O)

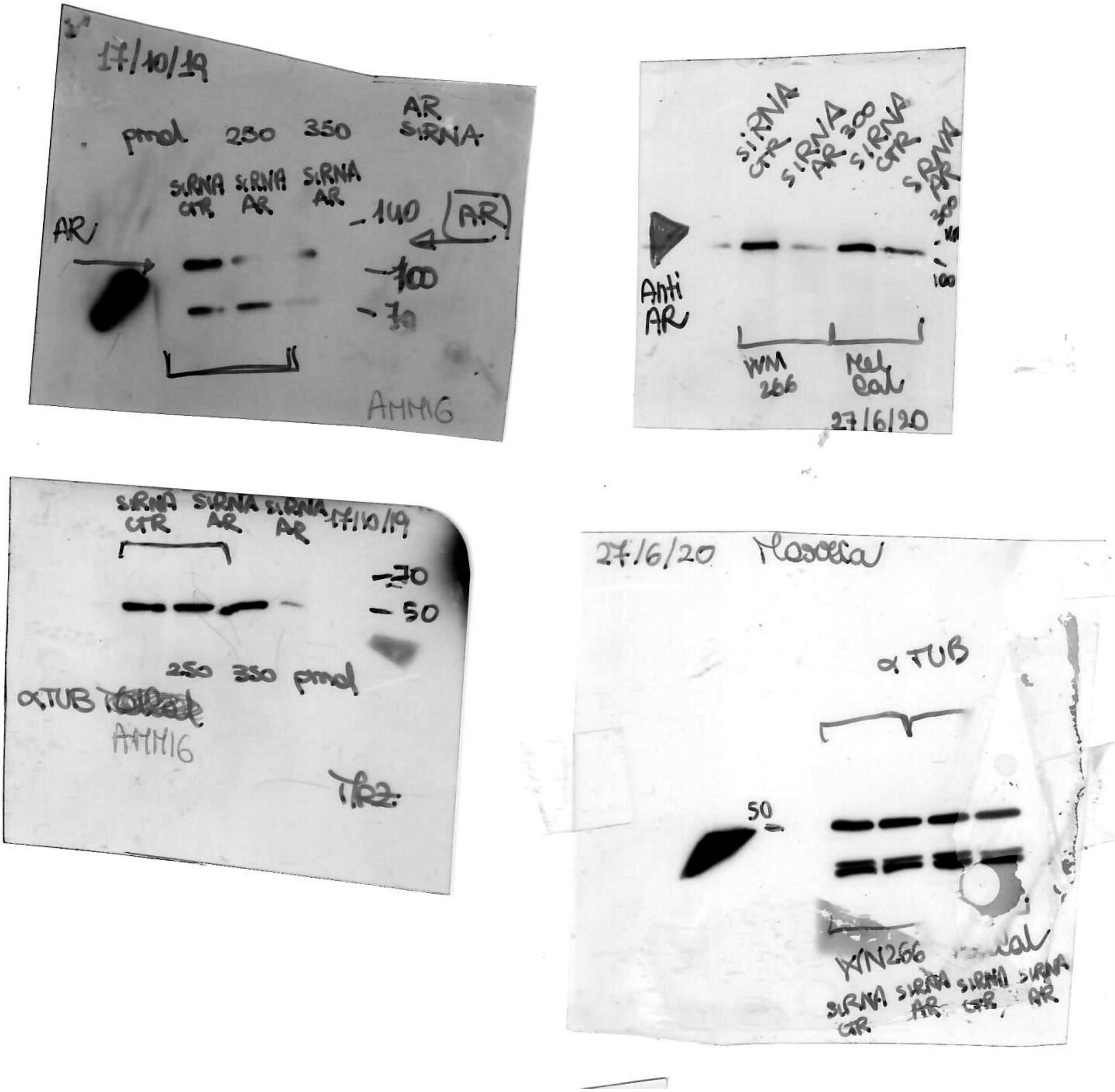
17/10/19
pmol 250 350
AR siRNA
siRNA CTR
siRNA AR
siRNA AR
140
AR
AR
100
70
AMM16
siRNA CTR
siRNA AR
siRNA AR
300
siRNA CTR
siRNA AR
Anti AR
100
WM 266
Mel cal
27/6/20
siRNA CTR siRNA AR siRNA AR 17/10/19
70
50
250 350 pmol
αTUB
AMM16
27/6/20
α TUB
50
WM266
siRNA CTR
siRNA AR
siRNA CTR
siRNA AR

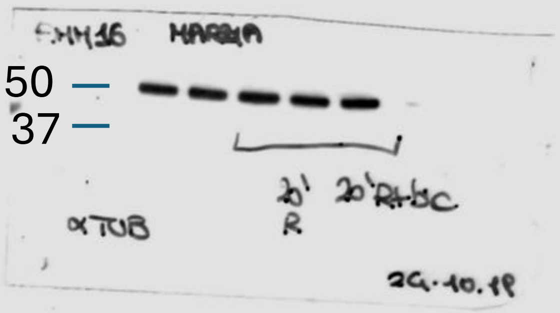

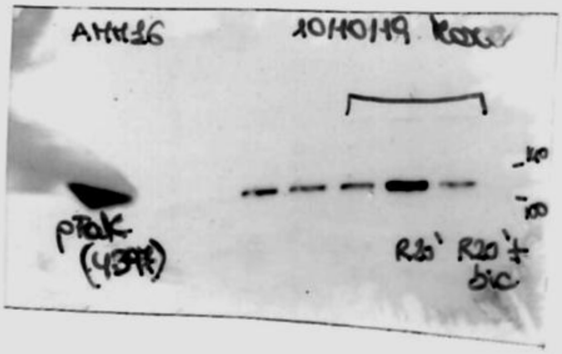

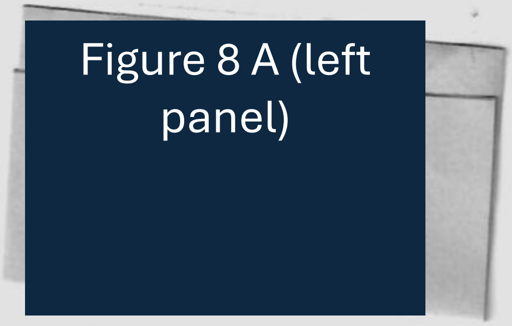

Figure 8 A (left panel)

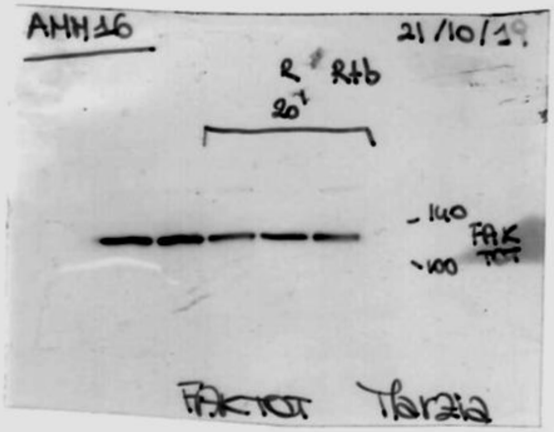

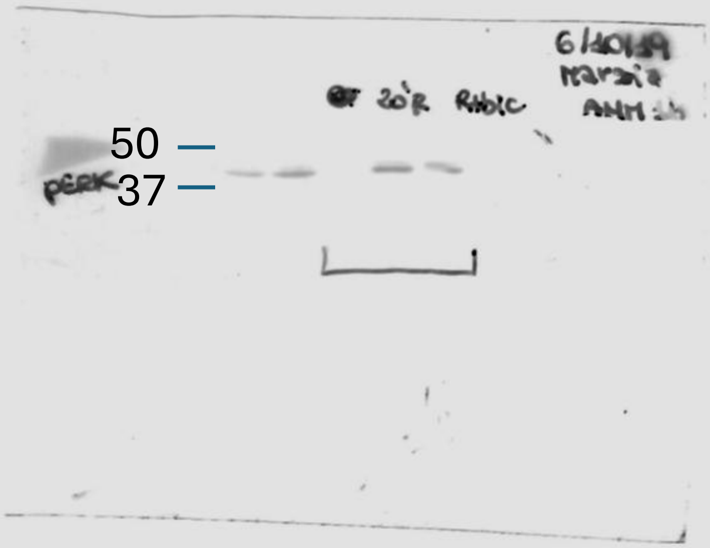

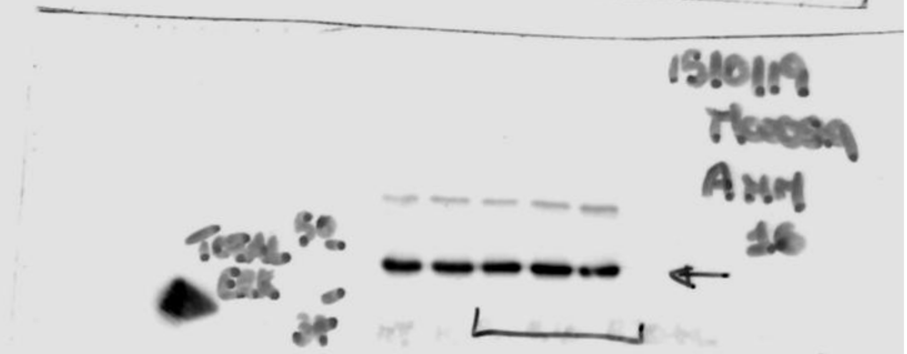

Figure 8 A (right panel)

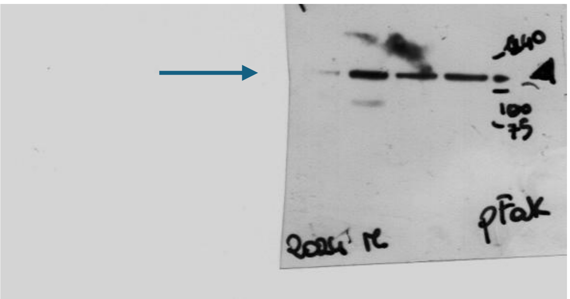

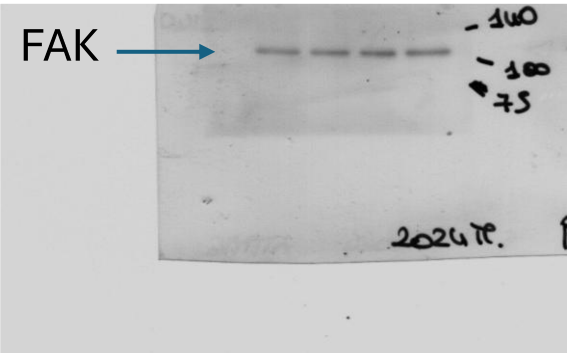

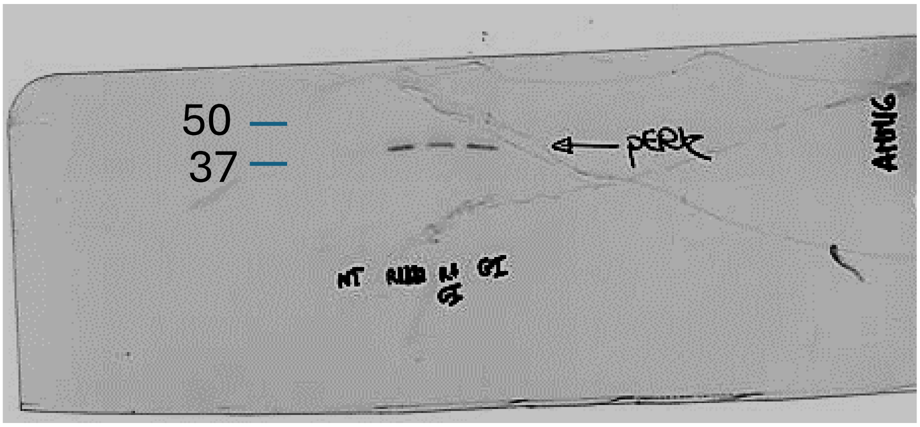

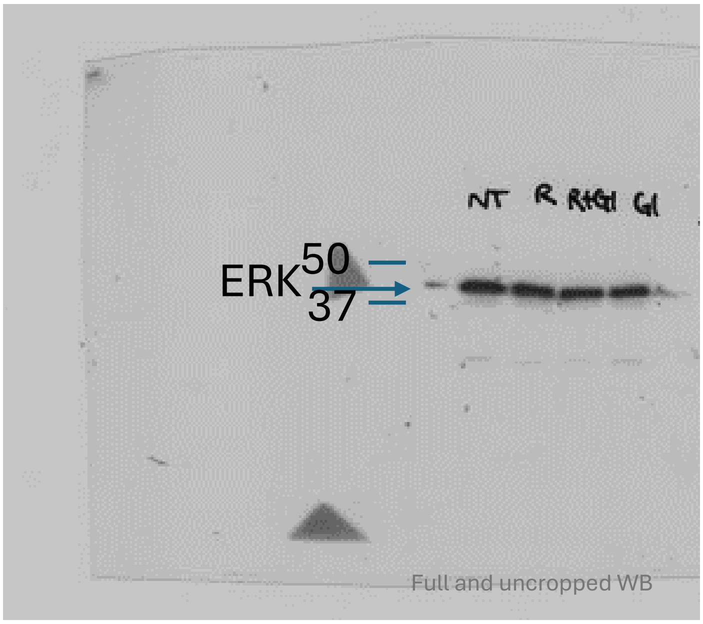

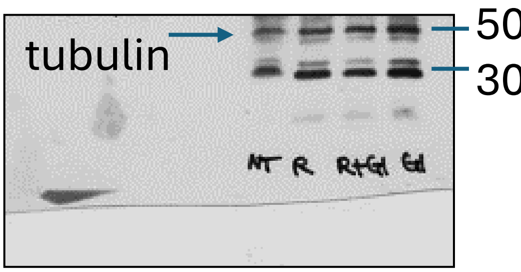

Figure 8 C

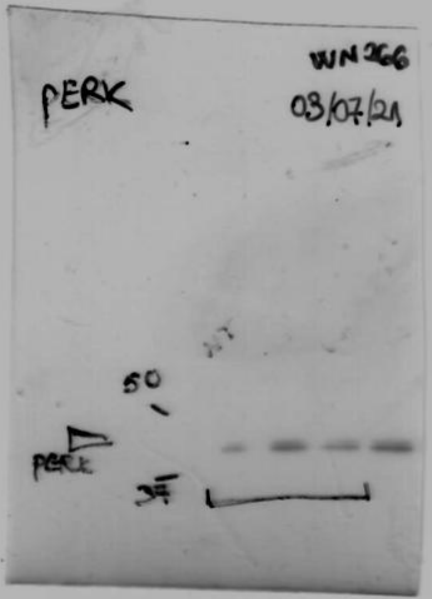
PERK
WN 266
03/07/21
50
pERK
37

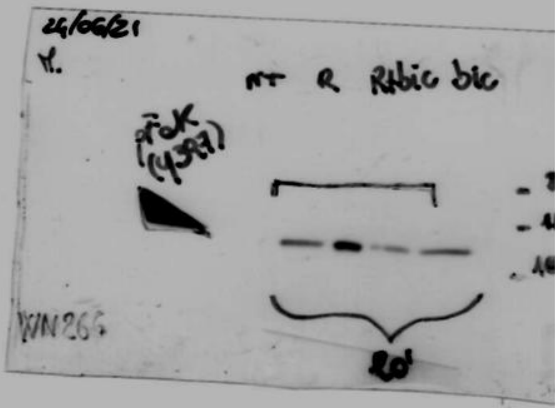
24/06/21
NT R R+bic bic
WN 266
20'

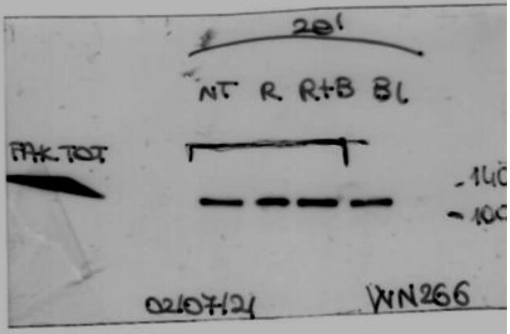
20'
NT R R+B Bi
FAK TOT
02/07/21
WN266

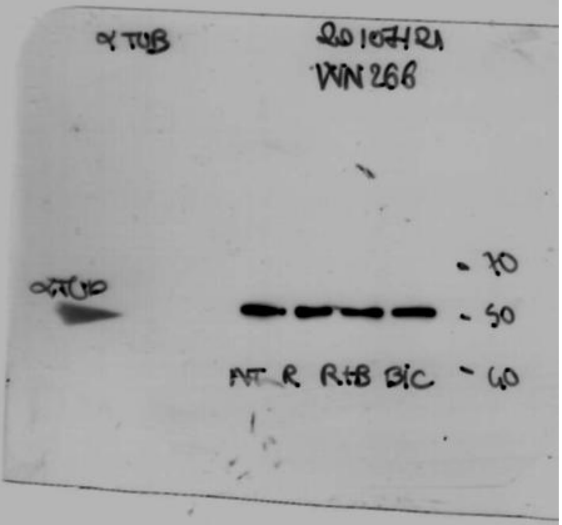
α TUB
20/07/21
WN 266
70
50
NT R R+B Bic
40

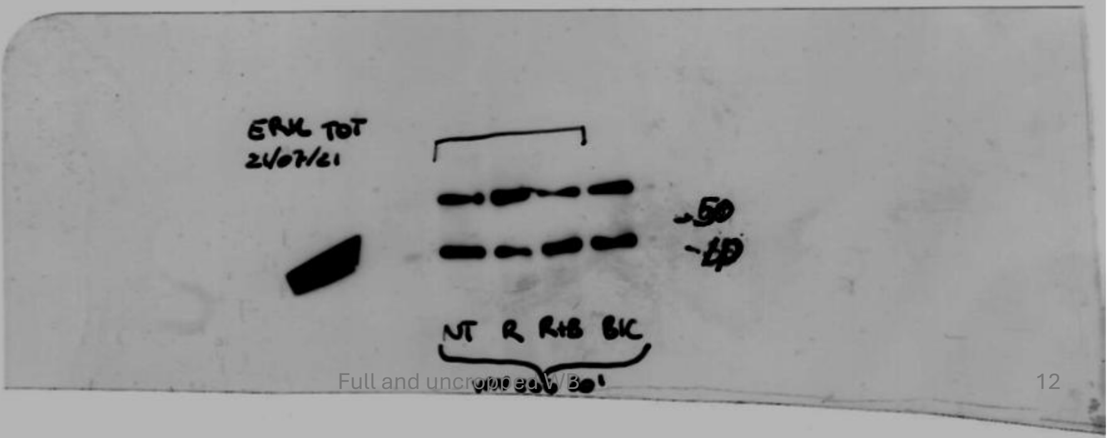
ERK TOT
21/07/21
50
40
NT R R+B BIC

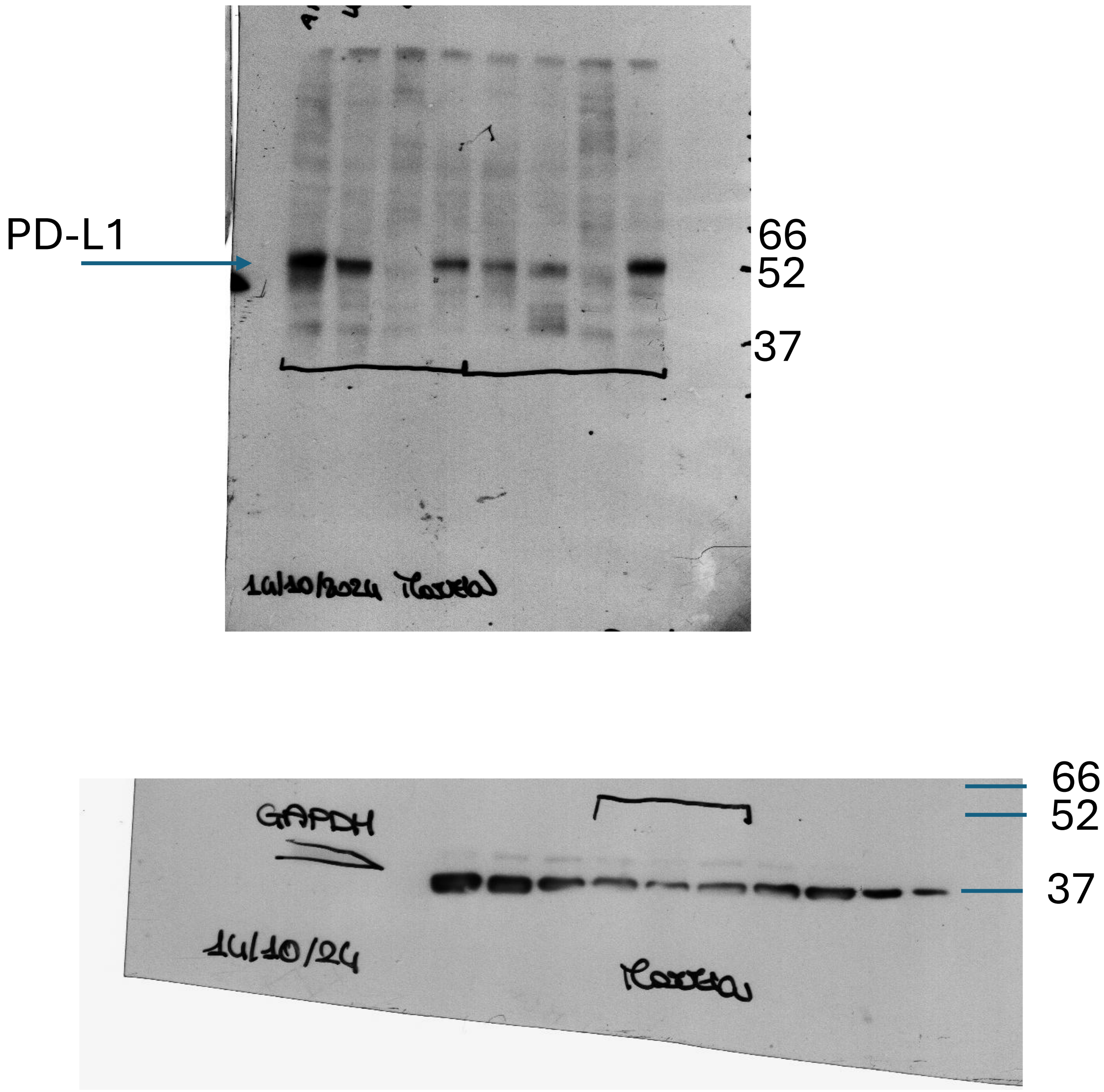

Figure 9 C

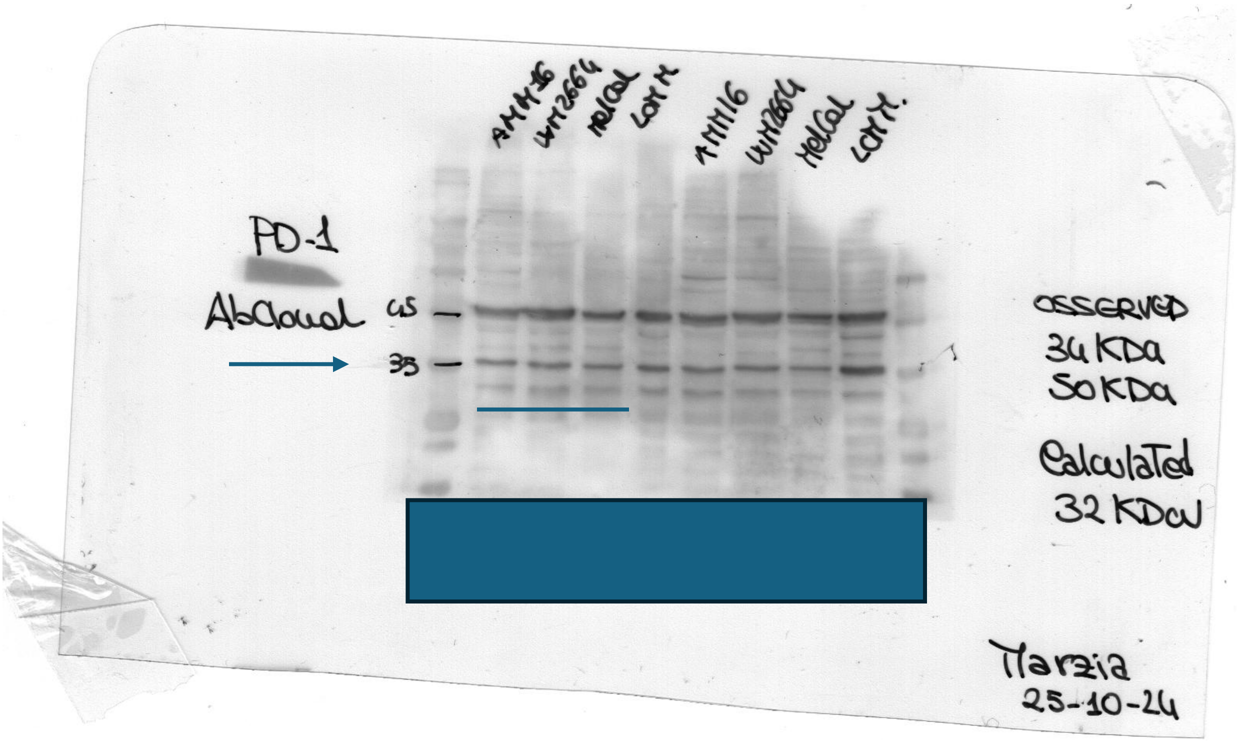
PD-1
Abclonal
45
33
OSSERVED
34 KDa
50 KDa
Calculated
32 KDa
Marzia
25-10-24

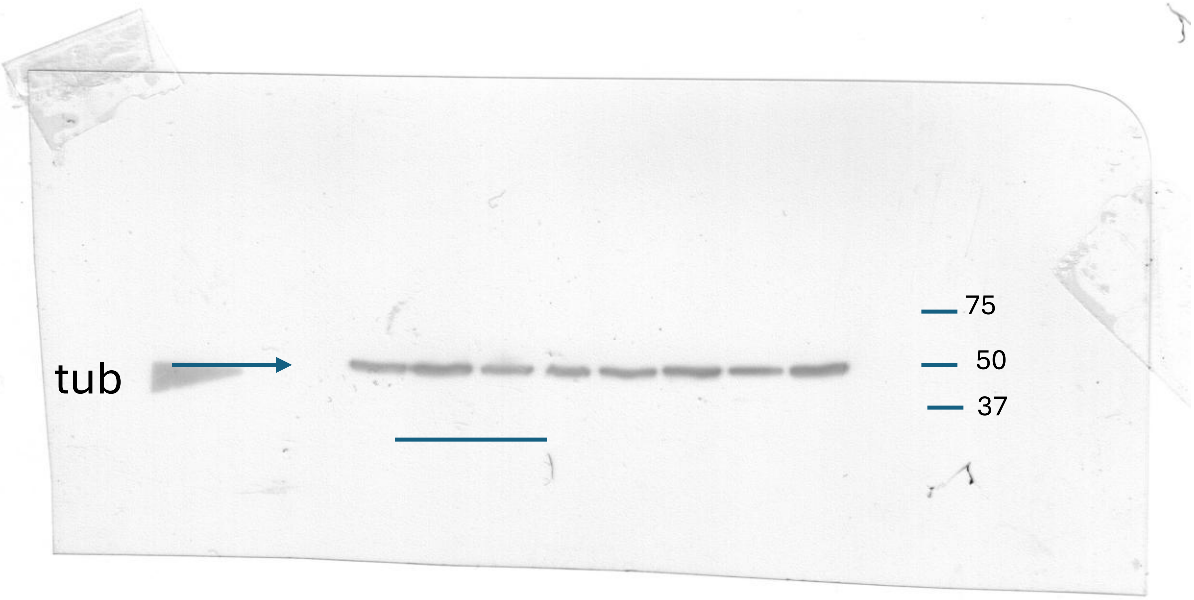
tub
75
50
37

Figure 9 D

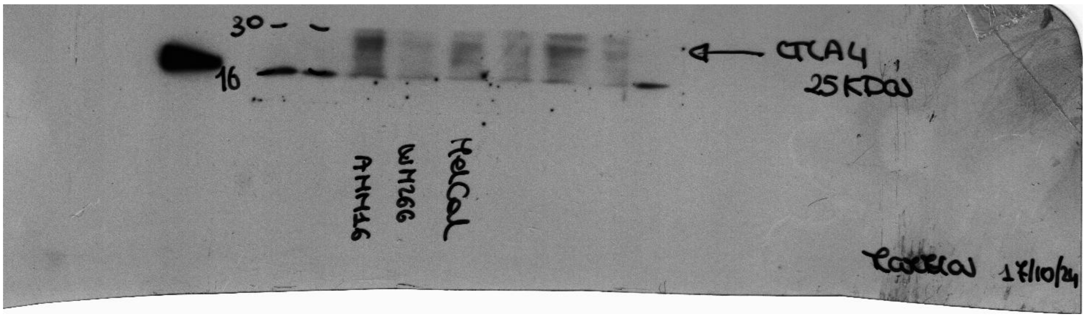

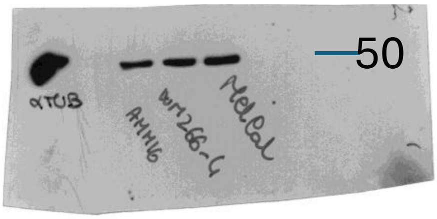

Figure 9 E

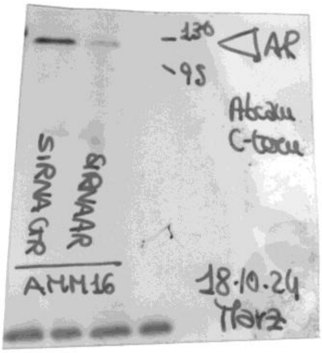

Figure 9 F

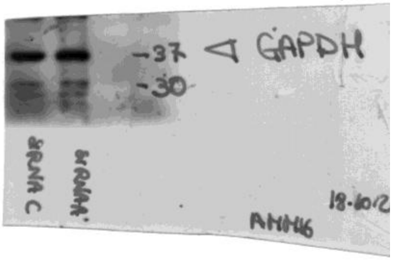

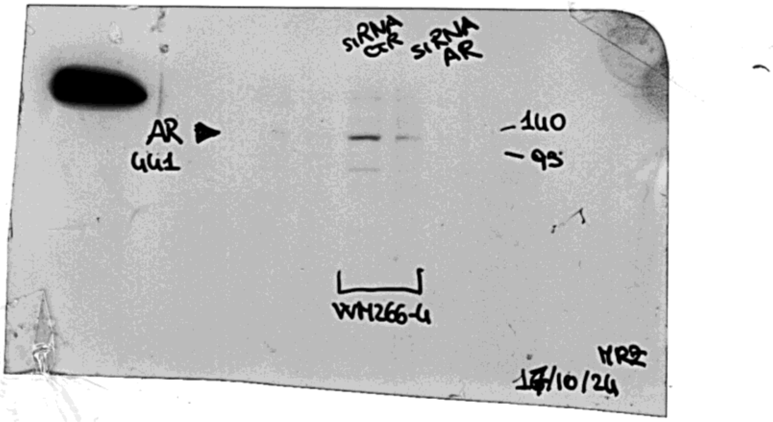

Figure 9 L

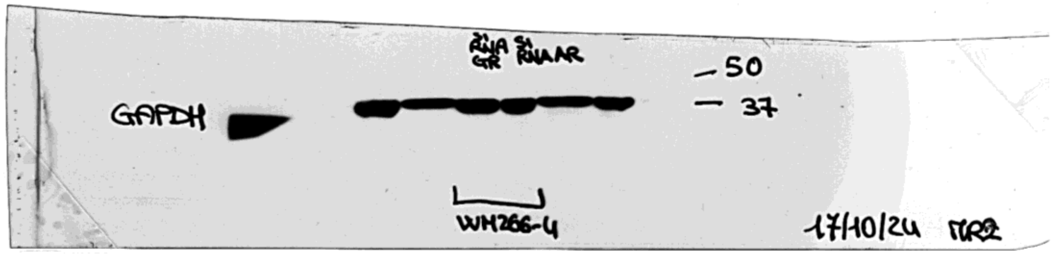

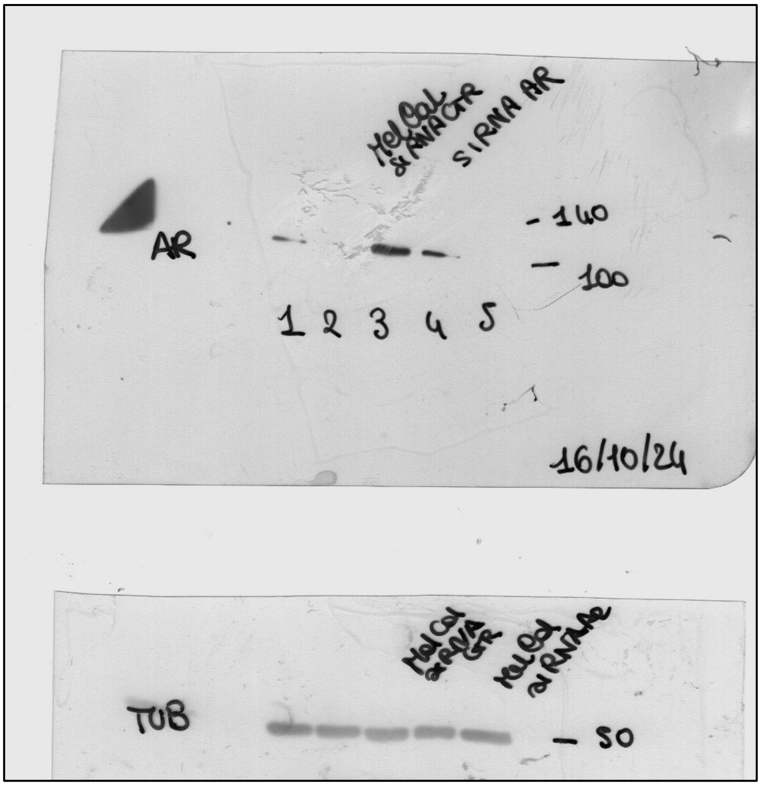

Figure 9 P

Figure S1, panel b

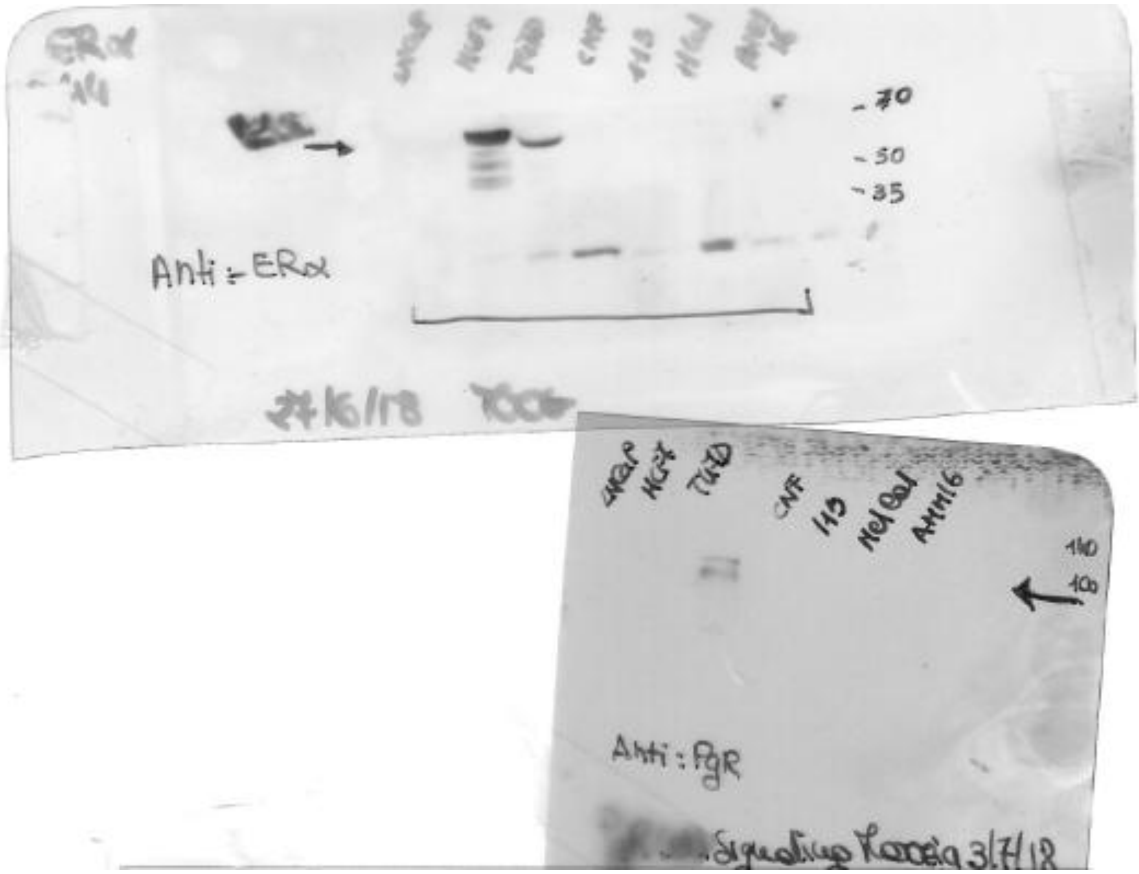

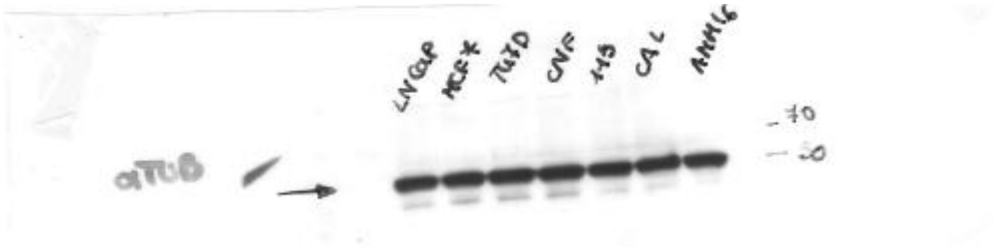

Figure S1, panel b

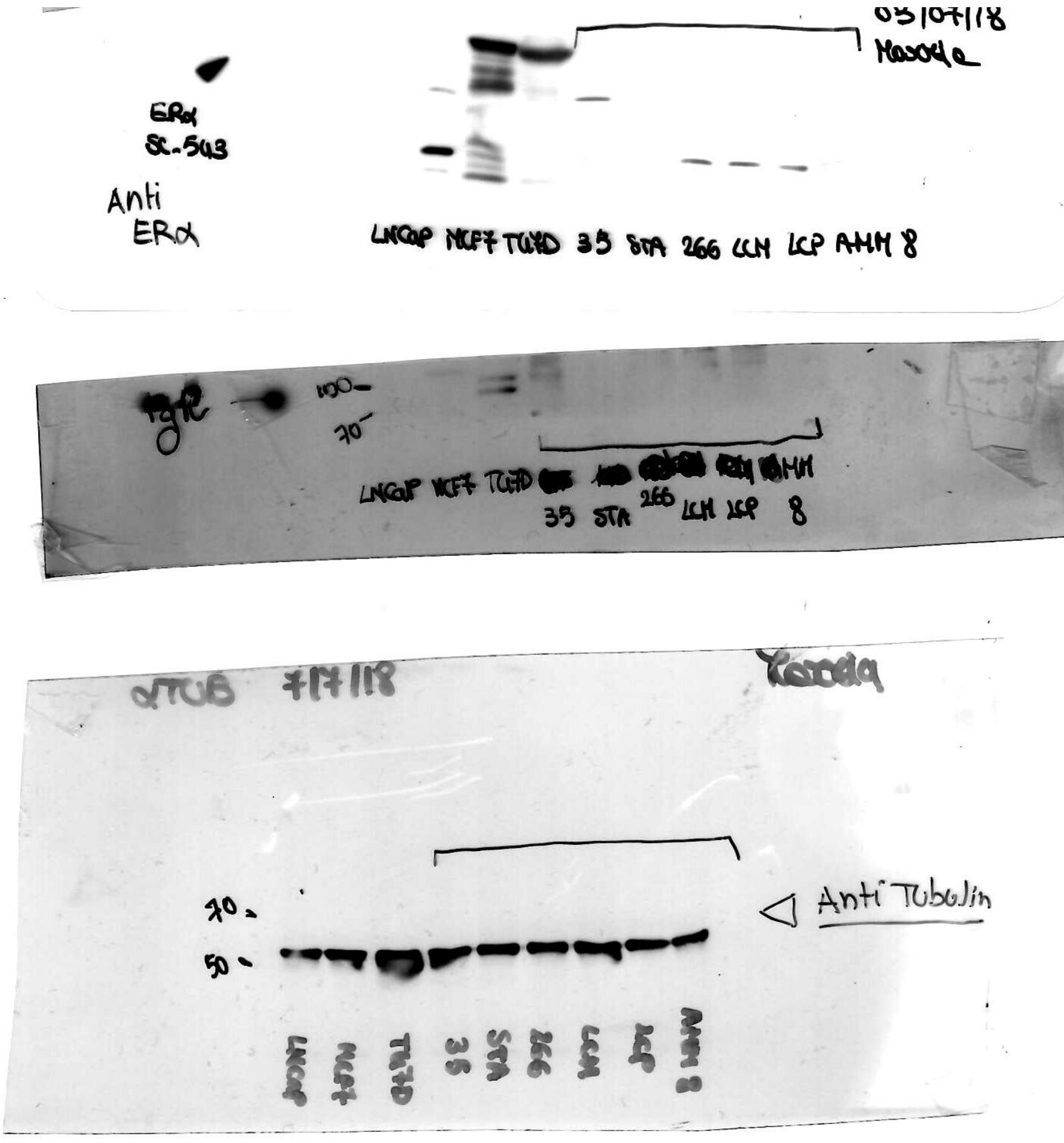

Figure S2

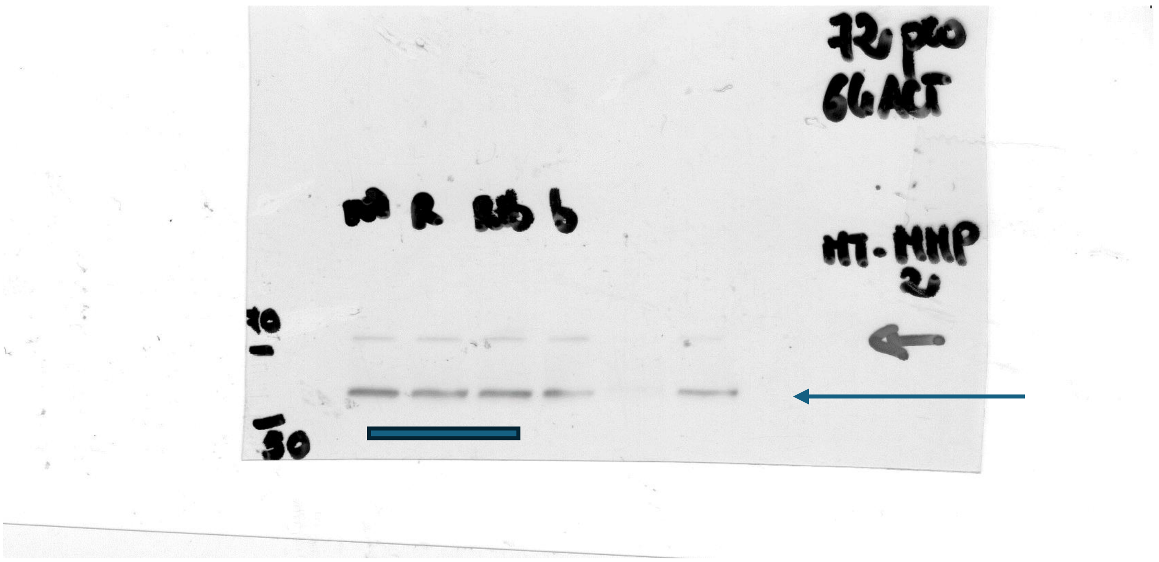

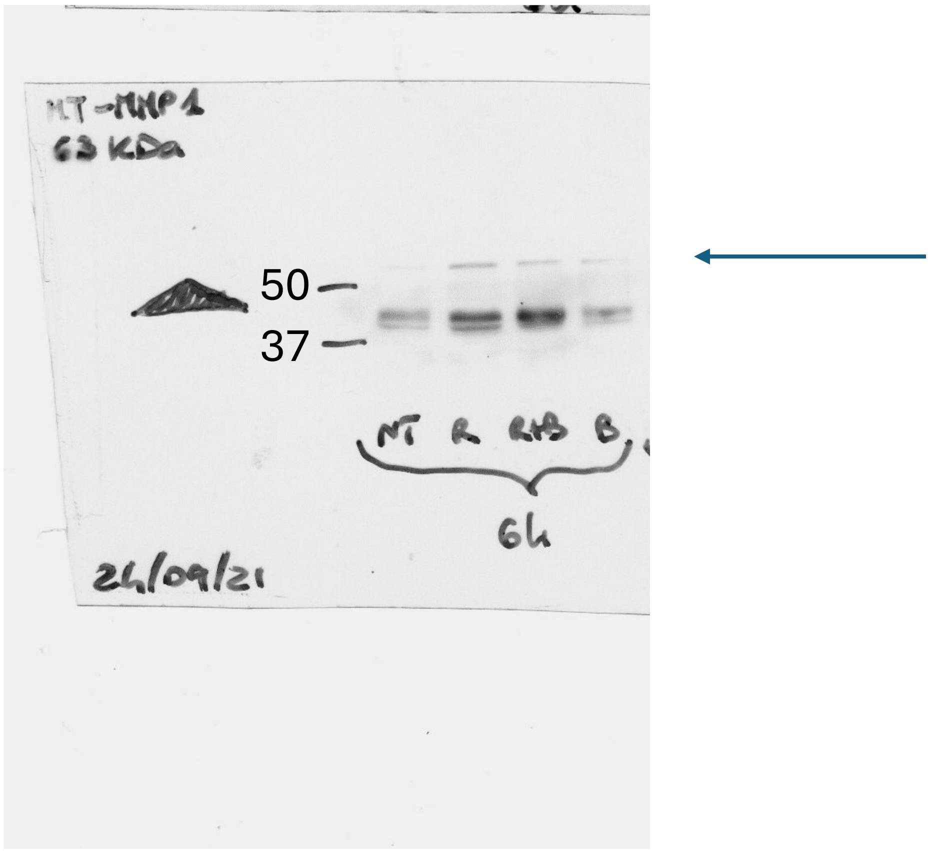

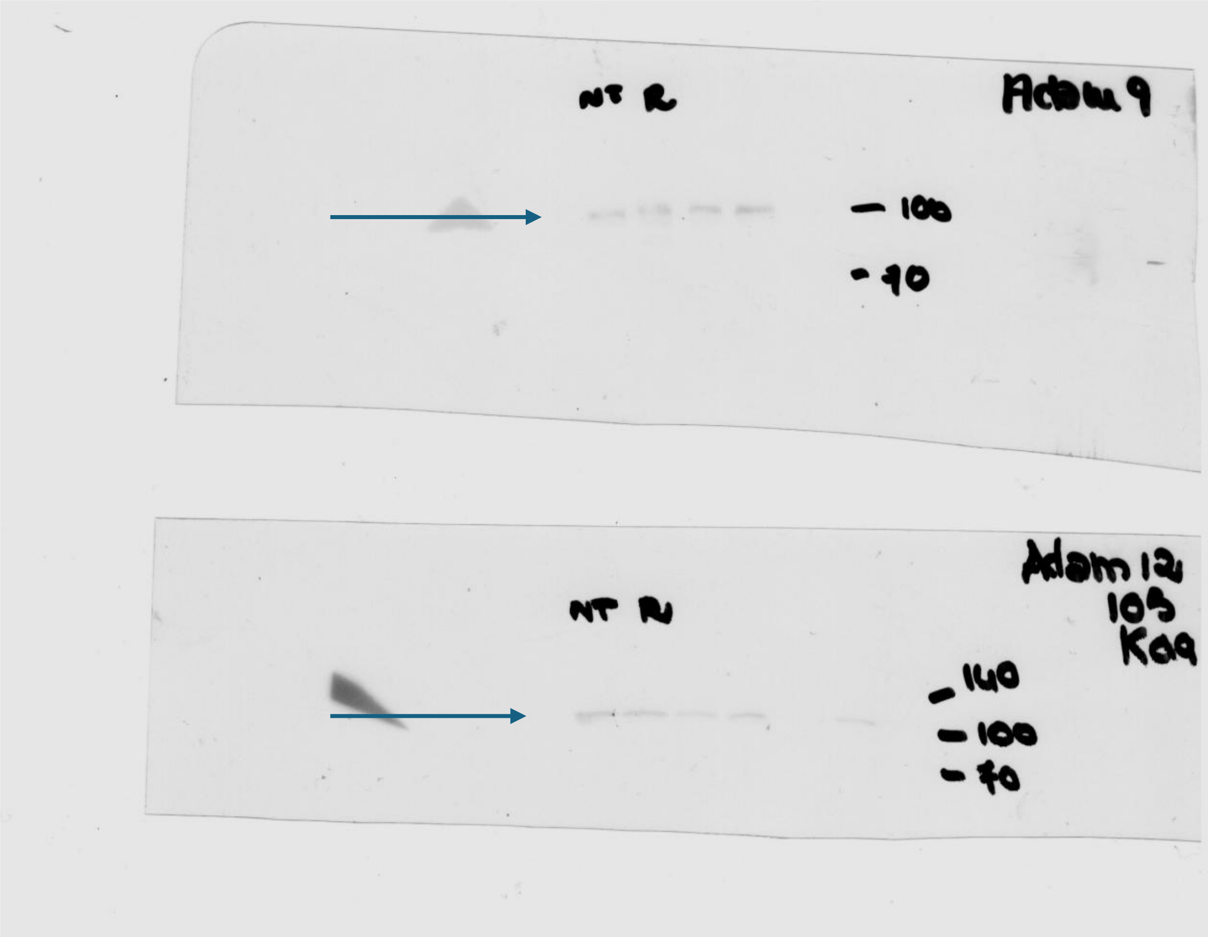
NT R
Adam 9
100
70
NT R
Adam 12
105
Kda
140
100
70
